# Gene expression profiling of *RIP2*-knockdown in HD11 macrophages — elucidation of potential pathways (gene network) when challenged with avian pathogenic *E.coli* (APEC)

**DOI:** 10.1186/s12864-022-08595-5

**Published:** 2022-05-02

**Authors:** Hongyan Sun, Yexin Yang, Yuxuan Cao, Huan Li, Lujiang Qu, Susan J. Lamont

**Affiliations:** 1 College of Animal Science and Technology, Yangzhou University, Yangzhou, 225009 China; 2 Joint International Research Laboratory of Agriculture & Agri-Product Safety, Ministry of Education, Yangzhou University, Yangzhou, 225009 China; 3 School of Biological and Chemical Engineering, Yangzhou Polytechnic College, Yangzhou, 225009 China; 4 College of Animal Science and Technology, China Agricultural University, Beijing, 100091 China; 5 Department of Animal Science, Iowa State University, Ames, Iowa 50011 USA

**Keywords:** *RIP2*-knockdown, APEC, HD11 macrophages, RNA-seq, Gene network

## Abstract

**Background:**

Receptor interacting serine/threonine kinase 2 (RIP2), ubiquitous in many tissue/cell types, is the key regulator of immune and inflammatory responses for many diseases, including avian pathogenic *E. coli* (APEC), which causes a wide variety of localized or systemic infections. However, the molecular mechanisms by which *RIP2* drives its transcriptional program to affect immune and inflammatory response upon APEC infection remains poorly understood.

**Results:**

In this study, RNA-seq and bioinformatics analyses were used to detect gene expression and new direct/indirect *RIP2* targets in the treatments of wild type HD11 cells (WT), *RIP2* knockdown cells (shRIP2), APEC stimulation cells (APEC), and *RIP2* knockdown cells combined with APEC infection (shRIP2 + APEC). The results revealed that a total of 4691 and 2605 differentially expressed genes (DEGs) were screened in shRIP2 + APEC vs. APEC and shRIP2 vs. WT, respectively. Functional annotation analysis showed that apoptosis, MAPK, p53, Toll-like receptor, and Nod-like receptor signaling pathways were involved in APEC-induced *RIP2* knockdown HD11 cells. By analyzing the enriched pathway and gene networks, we identified that several DEGs, including *HSP90AB1*, *BID*, and *CASP9* were targeted by *RIP2* upon APEC infection.

**Conclusion:**

As a whole, this study can not only provide data support for constructing gene networks of *RIP2* knockdown with APEC challenge but also provide new ideas for improving the immune and inflammatory response.

**Supplementary Information:**

The online version contains supplementary material available at 10.1186/s12864-022-08595-5.

## Background

Avian pathogenic *E. coli* (APEC), a type of extraintestinal pathogenic *E. coli*, is the causative agent of avian colibacillosis, which can result in significant economic losses due to the mortality and the reduced productivity of affected birds [1–3]. Although great progress has been made for the treatment of APEC, the disease is still hard to eradicate due to the diversity of APEC serotypes causing disease and the emergence of antimicrobial resistance among APEC [4, 5]. The pathogenesis of APEC is still not well understood but appears to influence apoptosis of immune cells and tissues injury [6–8]. Moreover, it has been demonstrated that APEC shared the similar genomic sequences with uropathogenic *E. coli* responsible for human urinary tract infections [9, 10], indicating the horizontal gene transfer ability and zoonotic potential of APEC. Therefore, it is critical to study the mechanism of the immune response and effectively prevent the excessive inflammation at genomic level.

Receptor interacting serine/threonine kinase 2 (RIP2) is the key molecule in the regulation of immune responses, inflammation, and cell death against viral and bacterial infections [11–14]. The N-terminal CARD of *RIP2* can bind to the CARD domain of procaspase-1 to activate NFκB signal cascade and initiate the pro-inflammatory pathways [15–17]. Moreover, it has been demonstrated that cells deficient of *RIP2* could decrease the activation of NFκB, resulting in impaired expression of IL6, TNFα, IP10 and reduced neutrophil infiltration, which alleviate the excessive inflammatory response [18–20]. The research of Homer et al. [21] discovered that RIP2 tyrosine kinase has a dual function in NOD2-dependent autophagy, that is RIP2 both sends a positive autophagy signal through the activation of p38 MAPK and relieves repression of autophagy mediated by the phosphatase PP2A. Currently, *RIP2* has been found to play an important role in the response of the avian host to an APEC infection [7]. Although it is considered the master regulator of immune and inflammatory response, it is not clear how the ubiquitous *RIP2* can direct the immune or inflammation-specific process.

In this study we attempted to unravel the gene network underlying *RIP2* regulation associated with APEC infection via RNA-seq analysis, using the chicken HD11 macrophage cell line. We studied the expression profiles of complete and knockdown of *RIP2* HD11 cells with or without 24 h APEC post-infection. Gene Ontology (GO) and Kyoto Encyclopedia of Genes and Genomes (KEGG) mechanistic analyses were performed taking into consideration the function of mRNAs, which changed their expression significantly in RNA-seq analysis. Altogether, our results suggest that *HSP90AB1*, *BID*, and *CASP9* were the new targets downstream of *RIP2*, whose depletion directly or indirectly caused the suppression of those genes. Thus, *HSP90AB1*, *BID*, and *CASP9* were involved in the immune and inflammatory response, while precisely *RIP2* could down-regulate those genes during APEC stimulation.

## Results

### *RIP2* inhibition level in chicken HD11 macrophages with or without APEC challenge

**Fig. 1:**
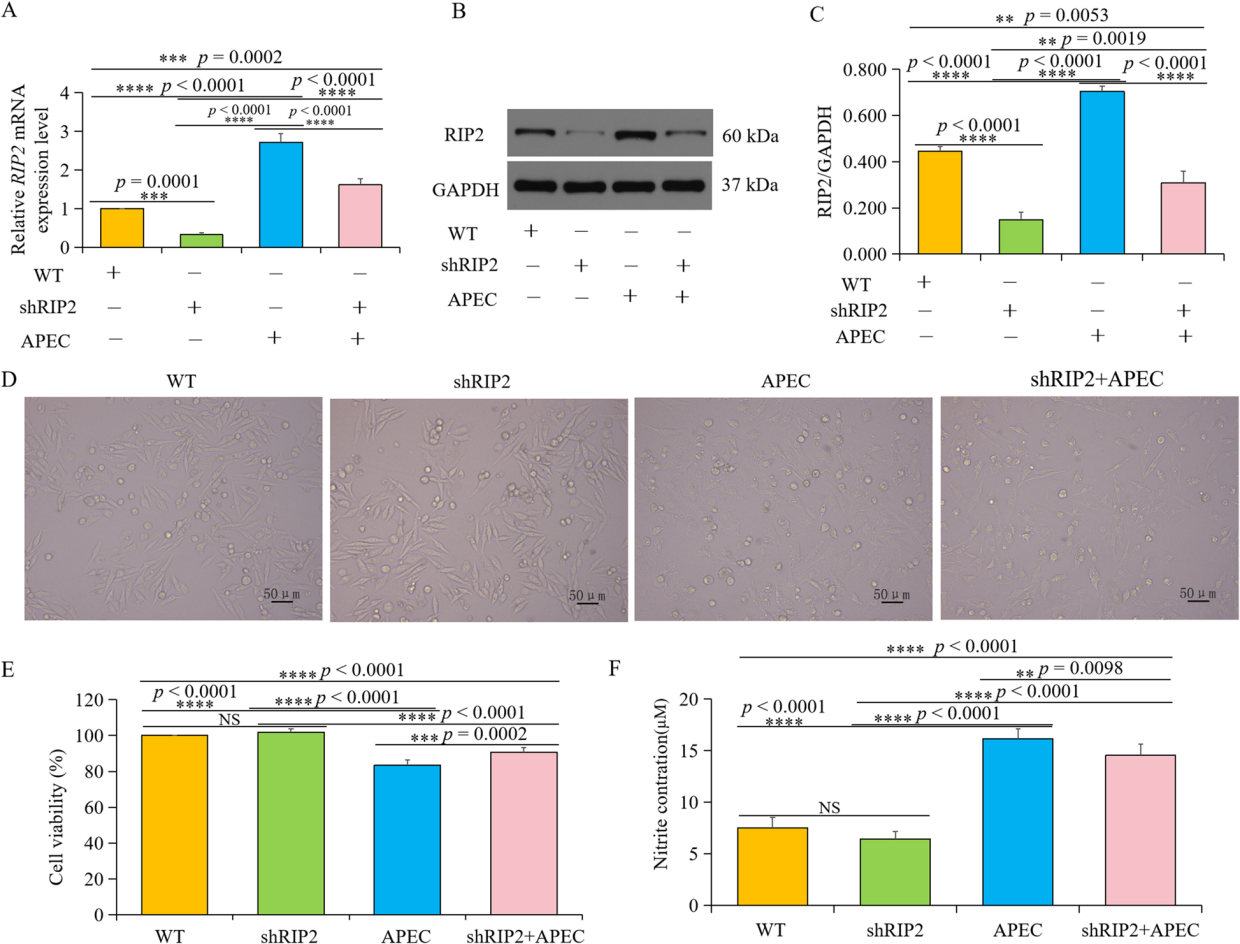
Knockdown of *RIP2* affects chicken HD11 macrophages with or without APEC challenge. **A** The relative *RIP2* mRNA expression level in the group of WT, shRIP2, APEC, and APEC+shRIP2 (data are shown as mean ± SD; *n* = 4 independent experiments; *** *p* < 0.001; **** *p* < 0.0001). **B** The RIP2 protein expression level in the group of WT, shRIP2, APEC, and APEC+shRIP2 was analyzed by western blot. **C** Image J software was used for RIP2 gray-level analysis of western blot results (data are shown as mean ± SD; *n* = 3 independent experiments; ** *p* < 0.01; **** *p* < 0.0001). **D** The morphology of chicken HD11 macrophages in the group of WT, shRIP2, APEC, and APEC+shRIP2. **E** The cell viability of chicken HD11 macrophages in the group of WT, shRIP2, APEC, and APEC+shRIP2 (data are shown as mean ± SD; *n* = 4 independent experiments; *** *p* < 0.001; **** *p* < 0.0001; NS, not significant). **F** The nitric oxide (NO) production of chicken HD11 macrophages in the group of WT, shRIP2, APEC, and APEC+shRIP2 (data are shown as mean ± SD; *n* = 4 independent experiments; ** *p* < 0.01; **** *p* < 0.0001; NS, not significant). Abbreviations: WT, wild type HD11 cells; shRIP2, *RIP2* knockdown HD11 cells; APEC, avian pathogenic *E.coli* challenge group

To identify the inhibition level of *RIP2*, the protein and mRNA levels were first measured in wild type HD11 cells (WT), HD11 cells with knockdown of *RIP2* (shRIP2), APEC-challenged HD11 cells (APEC), and APEC-challenged HD11 cells with knockdown of *RIP2* (shRIP2 + APEC). As shown in Fig. 1A and B, a significantly decreased mRNA and protein expression level of RIP2 were observed in the *RIP2* knockdown group in comparison to WT (*p* < 0.05), indicating the expression of *RIP2* was indeed inhibited. Also, APEC challenge resulted in the significantly increased mRNA and protein abundance of RIP2 compared with WT (*p* < 0.05). However, the mRNA and protein level of RIP2 were found to be significantly decreased in the *RIP2* knockdown HD11 cells with APEC challenge in comparison to wild type HD11 cells or APEC challenge group (Fig. 1A-C).

### Cell viability in knockdown of *RIP2* HD11 macrophages with or without APEC challenge

Morphological changes of APEC-challenged HD11 cells with or without knockdown of *RIP2* were observed. As displayed in Fig. 1D, there was no effect of *RIP2* knockdown on HD11 cells growth and proliferation. After challenge with APEC at 10^8^ cfu/mL for 24 h, cytopathic effects appeared in HD11 cells when compared with the WT, while knockdown of *RIP2* gene could significantly alleviate the APEC-challenged cytopathy. Moreover, the viability of chicken HD11 cells toward APEC challenge with or without knockdown of *RIP2* was measured using an CCK-8 assay. There was no difference between WT and shRIP2 group (*p* > 0.05; Fig. 1E), which was consistent with the results of cells morphology. The APEC-challenged chicken HD11 cells had a significantly lower cell survival rate than those in the *RIP2* knockdown group at 24 h post-infection (*p* < 0.05; Fig. 1E), indicating knockdown of *RIP2* can effectively inhibit the APEC-challenged cell apoptosis in HD11 cells.

### Nitric oxide (NO) production in knockdown of *RIP2* HD11 macrophages with or without APEC challenge

Considering the function of chicken HD11 macrophages, NO production in the cell supernatant from the four different groups (WT, shRIP2, APEC, and shRIP2 + APEC) was determined using the Griess reagent kit. As shown in Fig. 1F, there was no significant difference between WT and *RIP2* knockdown group for NO production. However, APEC challenge and *RIP2* knockdown combined with APEC challenge groups induced significant NO production in HD11 macrophages, with APEC challenged group giving higher levels of NO compared to *RIP2* knockdown combined with APEC challenged group (*p* < 0.05, Fig. 1F). These results suggested that knockdown of *RIP2* had the ability to effectively reduce the APEC-induced NO production, although the level was still significantly higher than WT (*p* < 0.05).

### Overview of RNA sequencing data

A total of twelve cDNA libraries were constructed, respectively, from four groups (WT, shRIP2, APEC, and shRIP2 + APEC) with three biological replicates for each. A total of 71,438,338-95,374,638 sequence reads were obtained, and each sample yielded approximately 80,223,040 clean reads (range from 69,393,540 to 92,952,070, Table S3). The percentages of Q30 for the clean reads was more than 99%, and the average of GC content of clean reads was 52.34% (Table S3). Moreover, 92.81–94.10% of the clean reads were found to successfully map to the chicken reference genome, of which 89.16–91.25% were uniquely mapped to genome (Table S4). Additionally, Table 1 shows the information on the fraction of reads mapping to features for each sample, i.e. any expressed parts of the genome. Interestingly, the percentage of reads mapping to coding sequence (CDS) in WT, shRIP2, and shRIP2 + APEC were 59.78, 59.91, and 60.06%, respectively, while that in APEC was 56.95% (Table 1).

**Table 1:** Read distribution in chicken genome

Sample	TSS_10 kb (%)	TSS_5 kb (%)	TSS_1 kb (%)	5’UTR (%)	CDS (%)	3’UTR (%)	TES_1 kb (%)	TES_5 kb (%)	TES_10 kb (%)	Intergenic (%)	Intron (%)
WT_1	817,934 (1.09)	465,652 (0.62)	264,356 (0.35)	1,709,799 (2.29)	44,927,089 (60.14)	3,539,667 (4.74)	894,691 (1.20)	1,329,951 (1.78)	951,088 (1.27)	5,371,654 (7.19)	14,436,393 (19.32)
WT_2	850,628 (1.16)	481,034 (0.65)	266,795 (0.36)	1,783,041 (2.43)	43,890,294 (59.73)	3,326,499 (4.53)	809,810 (1.10)	1,233,127 (1.68)	961,875 (1.31)	5,646,153 (7.68)	14,229,717 (19.37)
WT_3	854,892 (1.16)	484,649 (0.66)	270,098 (0.37)	1,807,517 (2.45)	43,846,172 (59.48)	3,296,988 (4.47)	796,623 (1.08)	1,220,494 (1.66)	969,557 (1.32)	5,770,236 (7.83)	14,400,640 (19.53)
shRIP2_1	901,795 (1.11)	542,484 (0.67)	298,909 (0.37)	1,883,579 (2.31)	48,547,638 (59.62)	4,049,765 (4.97)	913,946 (1.12)	1,340,140 (1.65)	975,162 (1.20)	6,235,343 (7.66)	15,737,712 (19.33)
shRIP2_2	811,832 (1.09)	499,462 (0.67)	276,288 (0.37)	1,758,489 (2.36)	44,794,360 (60.05)	3,562,251 (4.78)	799,364 (1.07)	1,188,481 (1.59)	903,916 (1.21)	5,775,924 (7.74)	14,220,256 (19.06)
shRIP2_3	712,708 (1.08)	435,538 (0.66)	246,408 (0.37)	1,554,302 (2.36)	39,621,169 (60.05)	3,136,809 (4.75)	702,608 (1.06)	1,046,395 (1.59)	807,534 (1.22)	5,049,179 (7.65)	12,662,782 (19.19)
APEC_1	1,200,954 (1.59)	513,130 (0.68)	267,424 (0.35)	1,719,332 (2.27)	42,694,064 (56.42)	3,344,929 (4.42)	903,188 (1.19)	1,368,388 (1.81)	942,420 (1.25)	6,327,762 (8.36)	16,388,478 (21.66)
APEC_2	1,089,300 (1.53)	477,721 (0.67)	254,628 (0.36)	1,636,220 (2.30)	40,618,885 (57.14)	3,027,199 (4.26)	816,604 (1.15)	1,247,046 (1.75)	902,012 (1.27)	5,917,683 (8.33)	15,094,873 (21.24)
APEC_3	1,108,637 (1.52)	486,009 (0.67)	258,748 (0.35)	1,654,734 (2.27)	41,844,050 (57.28)	3,137,449 (4.29)	858,367 (1.17)	1,294,858 (1.77)	923,393 (1.26)	5,923,740 (8.11)	15,564,581 (21.31)
shRIP2 + APEC_1	913,505 (1.13)	530,763 (0.66)	293,283 (0.36)	1,893,473 (2.34)	48,220,437 (59.62)	4,036,600 (4.99)	878,217 (1.09)	1,280,225 (1.58)	776,896 (0.96)	6,356,734 (7.86)	15,702,773 (19.41)
shRIP2 + APEC_2	1,005,052 (1.15)	580,628 (0.66)	322,426 (0.37)	2,117,408 (2.42)	52,630,365 (60.20)	4,073,560 (4.66)	880,221 (1.01)	1,301,687 (1.49)	853,729 (0.98)	6,930,910 (7.93)	16,725,203 (19.13)
shRIP2 + APEC_3	893,086 (1.13)	514,527 (0.65)	287,291 (0.36)	1,876,382 (2.37)	47,741,694 (60.37)	3,806,973 (4.81)	824,719 (1.04)	1,214,147 (1.54)	761,941 (0.96)	6,150,964 (7.78)	15,008,025 (18.98)

*WT* indicates wild type HD11 cells, *shRIP2* represents knockdown of *RIP2* HD11 cells, *APEC* indicates avian pathogenic *E. coli*; APEC+shRIP2 represents knockdown of *RIP2* HD11 cells combined with APEC infection

**Fig. 2:**
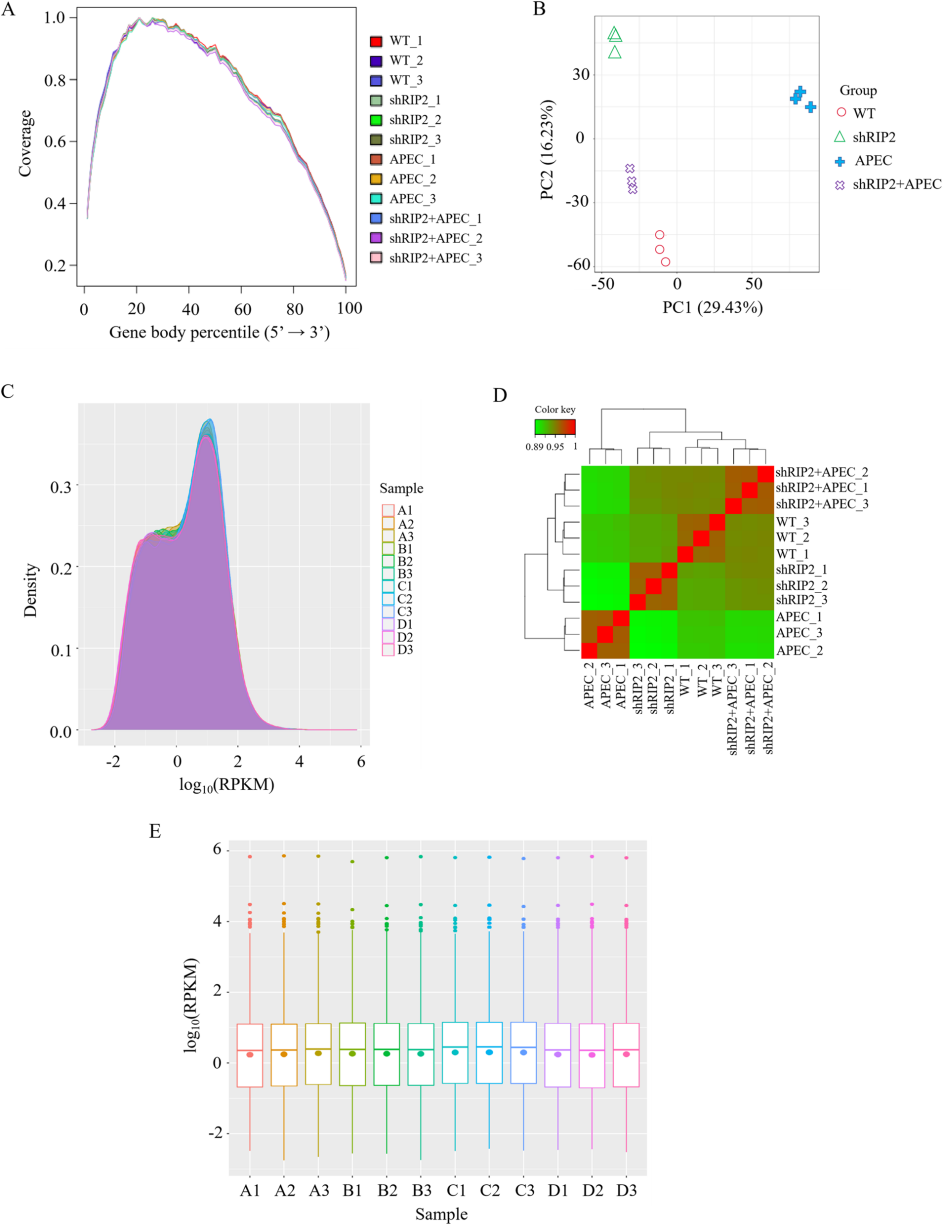
Reads and samples homogeneity, as well as the RPKM distribution for the group of *RIP2* knockdown HD11 macrophages (shRIP2), wild type HD11 macrophages (WT), avian pathogenic *E. coli* infection HD11 cells (APEC), *RIP2* knockdown combined with avian pathogenic *E. coli* infection HD11 cells (shRIP2 + APEC). **A** Comprehensive display of sequence coverage on the 5′ to 3′ region of all genes in the samples. **B** Multidimensional scaling plots for the normalized count data in mRNA samples collected from the group of shRIP2, WT, APEC, and shRIP2 + APEC. **C** Box plot of RPKM distribution with logarithmic values of RPKM on the vertical axis and different samples on the horizontal axis. A1, A2, and A3 indicate *RIP2* knockdown HD11 macrophages (shRIP2). B1, B2, and B3 represent wild type HD11 macrophages (WT). C1, C2, and C3 mean avian pathogenic *E. coli* infection HD11 cells (APEC). D1, D2, and D3 indicate *RIP2* knockdown combined with avian pathogenic *E. coli* infection HD11 cells (shRIP2 + APEC). **D** The heatmap of samples correlation. Abbreviations: WT, wild type HD11 cells; shRIP2, *RIP2* knockdown HD11 cells; APEC, avian pathogenic *E.coli* challenge group. **E** Density plot of expression distribution with density values on the vertical axis and logarithmic values of RPKM on the horizontal axis. A1, A2, and A3 indicate *RIP2* knockdown HD11 macrophages (shRIP2). B1, B2, and B3 represent wild type HD11 macrophages (WT). C1, C2, and C3 mean avian pathogenic *E. coli* infection HD11 cells (APEC). D1, D2, and D3 indicate *RIP2* knockdown combined with avian pathogenic *E. coli* infection HD11 cells(shRIP2 + APEC)

Also, sequencing homogeneity was assessed to evaluate the bias of RNA-seq data. Results showed reads were evenly distributed on the genes without 5′ or 3′ bias (Fig. 2A). Then, a multidimensional scaling analysis was performed on the count data, revealing the biological replicates were relatively concentrated in each of the four groups (Fig. 2B). The WT and shRIP2 HD11 macrophages with or without APEC infection were clearly separated (Fig. 2B). Furthermore, the heatmap of sample correlation showed the shRIP2 + APEC group clustered with the WT, and then the shRIP2, finally the APEC group (Fig. 2D), which were consistent with the results of reads distribution to CDS in Table 1. Then, the dynamic range of the expression values was estimated and exhibited as a box plot of logarithmic transformed RPKM values for each sample separately (Fig. 2C), and the RPKM density distribution is presented in Fig. 2E.

### Identification of differentially expressed genes (DEGs)

**Fig. 3:**
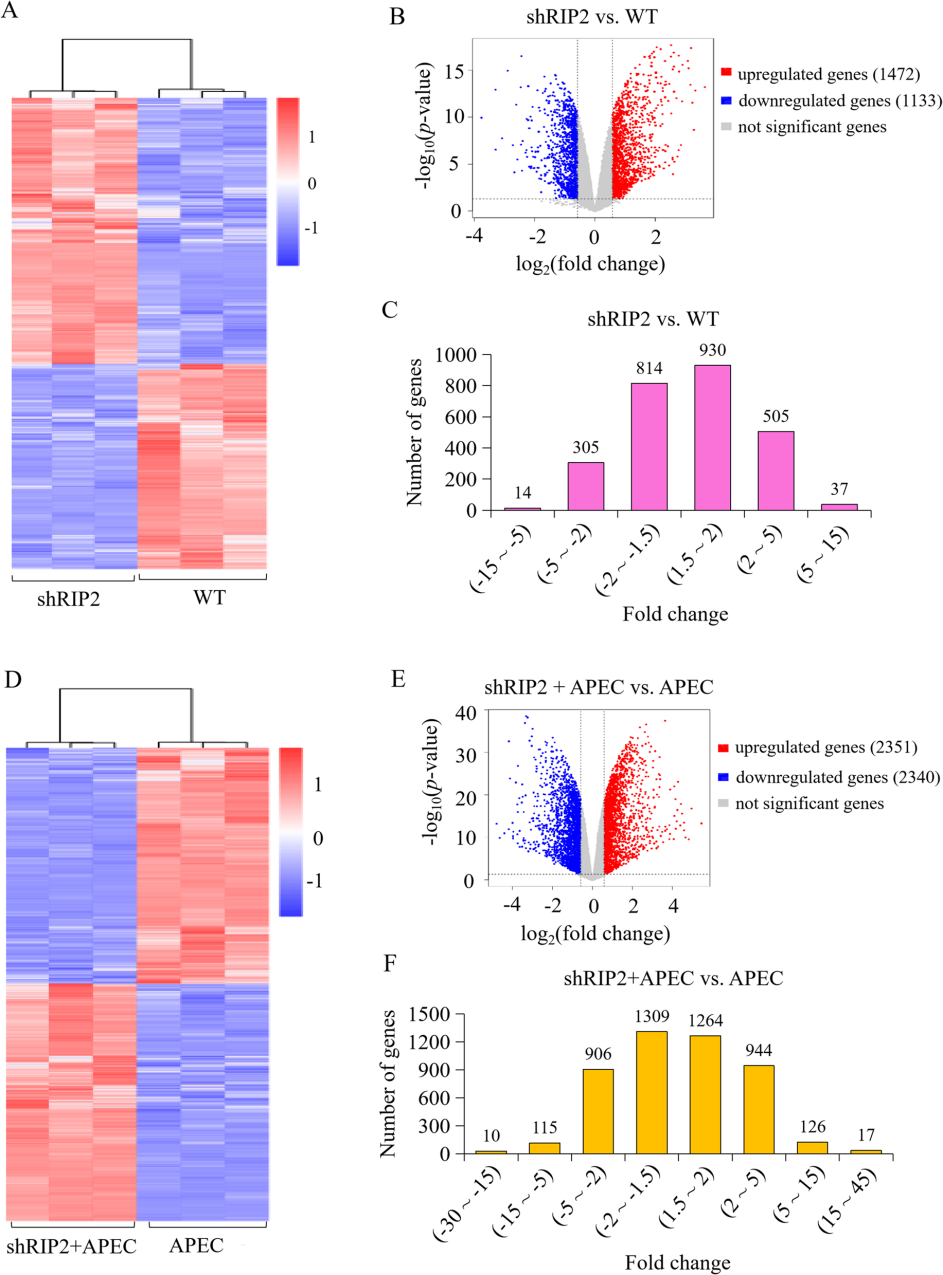
RNA-seq profiling in the comparisons of *RIP2* knockdown HD11 macrophages group (shRIP2) vs. wild type cells group (WT) and *RIP2* knockdown HD11 macrophages combined with APEC infection group (shRIP2 + APEC) vs. APEC infection group (APEC). **A** Heatmap analysis for the transcriptome data from the comparison of shRIP2 vs. WT. Red color indicate upregulation, while bule means downregulation. **B** The expression levels of differentially expressed genes (DEGs) in the comparison of shRIP2 vs. WT. Red spots represent DEGs for upregulation, blue spots for downregulation, and grey spots for unchanged genes in the comparison of shRIP2 vs. WT. **C** The distribution of the differentially expressed genes (DEGs) in the comparison of shRIP2 vs. WT. **D** Heatmap analysis for the transcriptome data from the comparison of shRIP2 + APEC vs. APEC. Red color indicate upregulation, while bule means downregulation. **E** The expression levels of the differentially expressed genes (DEGs) in the comparison of shRIP2 + APEC vs. APEC. Red spots represent DEGs for upregulation, blue spots for downregulation, and grey spots for unchanged genes in the contrast of shRIP2 + APEC vs. APEC. **F** The distribution of the differentially expressed genes (DEGs) in the comparison of shRIP2 + APEC vs. APEC

In order to investigate the specific mechanism that *RIP2* affected cell immune and inflammatory response upon APEC infection, DEGs were identified from WT and shRIP2 HD11 cells with or without APEC challenge. Pairwise comparisons were performed as follows: shRIP2 (knockdown of *RIP2* HD11 cells) vs. WT (complete HD11 macrophages) and shRIP2 + APEC (knockdown of *RIP2* HD11 cells combined with avian pathogenic *E. coli* infection) vs. APEC (avian pathogenic *E. coli* infection HD11 cells). A total of 2605 DEGs were obtained when adjusted *p*-value ≤0.05 and |logFC| ≥ 0.58 were set as the cut-off limits (Fig. 3A, B) in shRIP2 vs. WT. Among them, 1133 down-regulated DEGs and 1472 up-regulated DEGs were identified (Fig. 3C). In shRIP2 + APEC vs. APEC, a total of 4691 DEGs were identified (Fig. 3D, E), of which 2340 DEGs were down-regulated and 2351 DEGs were up-regulated (Fig. 3F).

### Functional annotation of DEGs in different comparisons

**Fig. 4:**
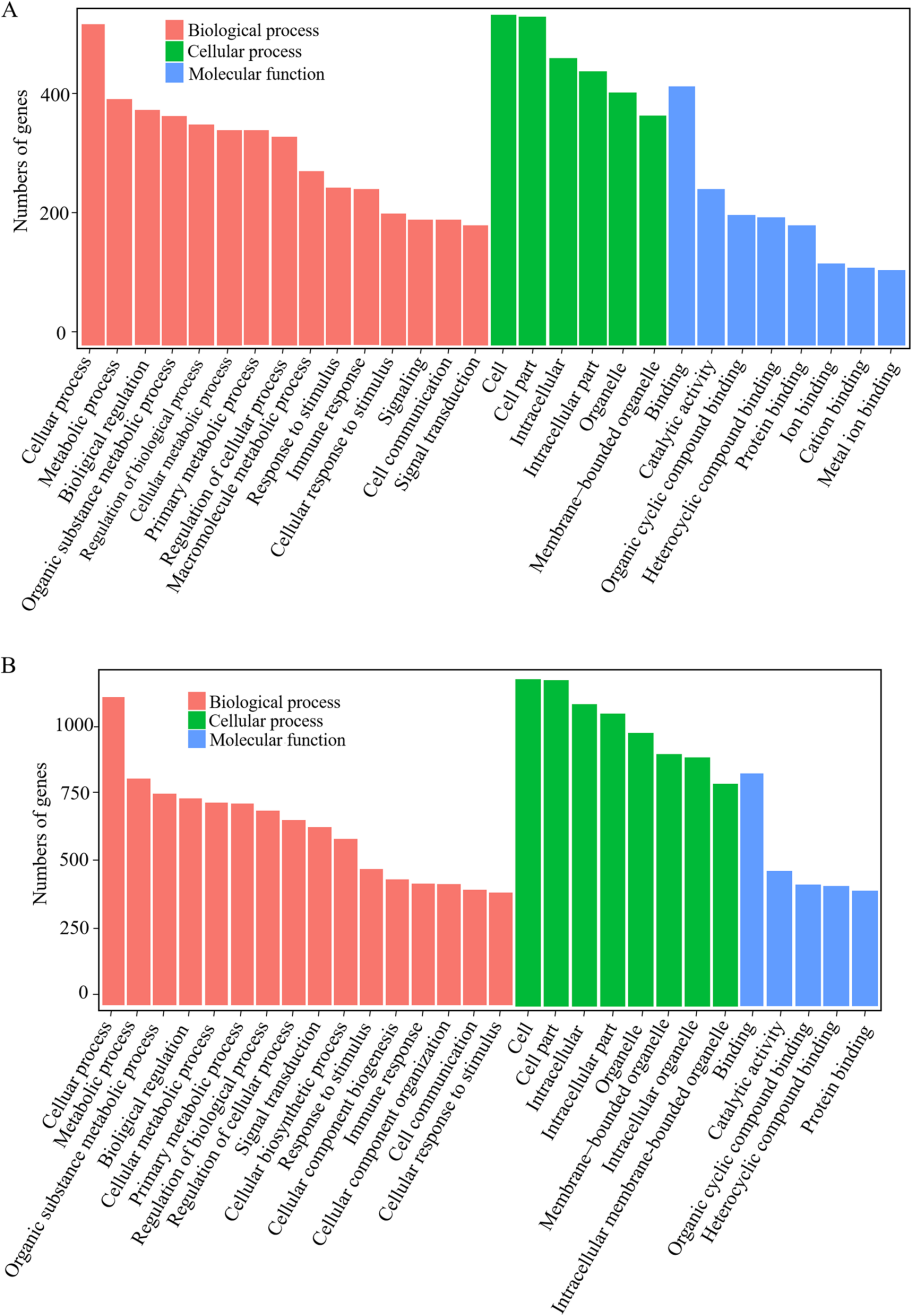
Gene classification was based on Gene Ontology (GO) analysis for differentially expressed genes (DEGs). **A** Different classes are shown for biological processes, cellular components, and molecular functions in the comparison of *RIP2* knockdown HD11 cells (shRIP2) vs. wild type (WT). **B** Different classes are shown for biological processes, cellular components, and molecular functions in the comparison of *RIP2* knockdown HD11 cells combined with avian pathogenic *E. coli* infection (shRIP2 + APEC) vs. avian pathogenic *E. coli* infection cells (APEC)

The gene ontology (GO) classification system was used to classify the possible functions of DEGs in different comparisons. A total of 734 genes (28.18%) and 1476 (31.46%) were successfully assigned to at least one GO term annotation in shRIP2 vs. WT (Table S5) and shRIP2 + APEC vs. APEC (Table S6), respectively. The significantly enriched GO terms in shRIP2 vs. WT and shRIP2 + APEC vs. APEC were similar (Fig. 4). For the molecular function category, the top two largest categories were “binding” and “catalytic activity”. According to biological process, the top categories were “cellular process”, “biological regulation”, and “metabolic process”. More remarkable, the immune related GO terms were also identified, including “cell communication”, “immune response”, “signal transduction”, and “response to stimulus”. These results show that knockdown of *RIP2* significantly affected immune system processes in chicken HD11 macrophages with or without APEC challenge.

**Fig. 5:**
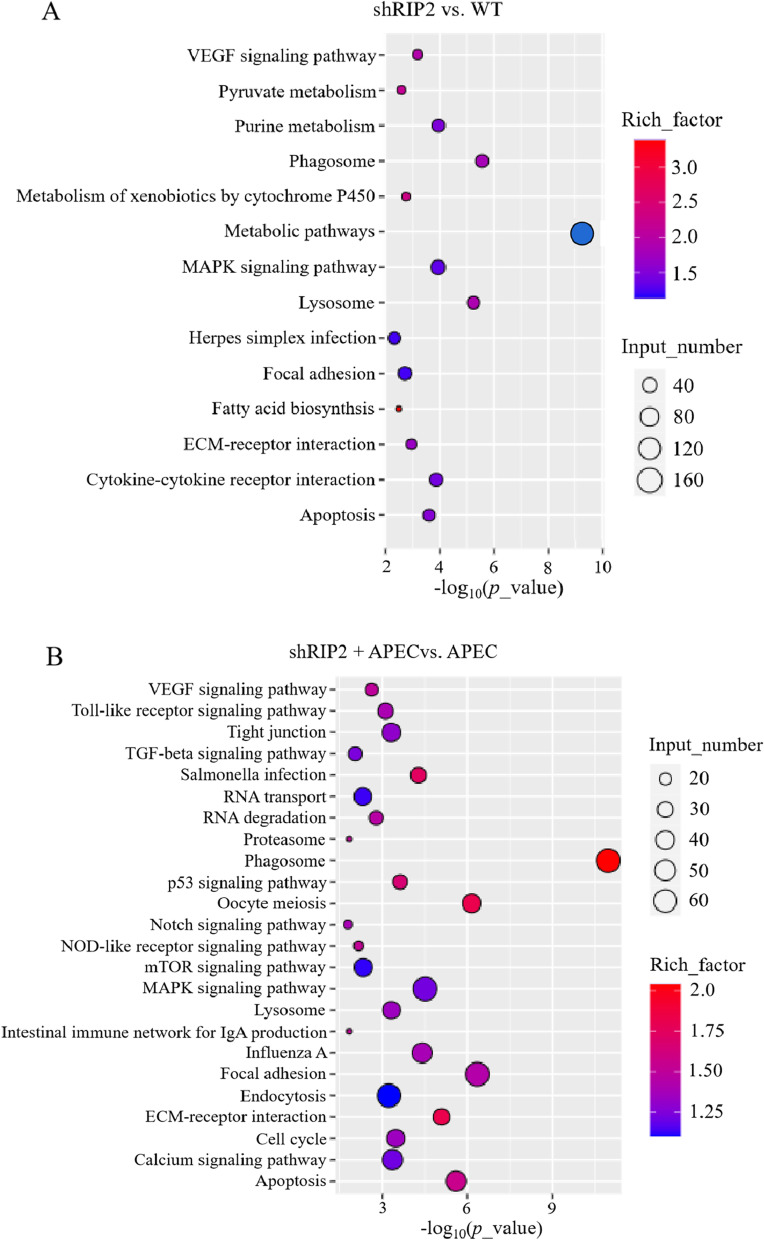
The significantly enriched signaling pathways in the comparison of *RIP2* knockdown HD11 macrophages (shRIP2) vs. wild type HD11 cells (WT) and *RIP2* knockdown HD11 macrophages combined with avian pathogenic *E. coli* (APEC) infection vs. APEC infection. **A** The significantly changed KEGG pathways in the comparison of shRIP2 vs. WT. **B** The significantly changed KEGG pathways in the comparison of shRIP2 + APEC vs. APEC

Then, Kyoto Encyclopedia of Genes and Genomes (KEGG) classification system was also performed to identify the possible functions of DEGs. A total of 15 and 55 significantly changed pathways were detected in shRIP2 vs. WT (Table S7) and shRIP2 + APEC vs. APEC (Table S8), respectively, with an adjusted *p*-value ≤0.05. The significantly enriched pathways were primarily involved in immune system for the two comparisons, which included “Phagosome”, “Lysosome”, “MAPK signaling pathway” (Fig. 5). In addition, the pathways related to cell growth, differentiation, survival, signal transduction were also significantly enriched in the two comparisons, including “Apoptosis”, “ECM-receptor interaction”, “VEGF signaling pathway”, and “Focal adhesion” (Fig. 5). However, the “p53 signaling pathway”, “cell cycle”, “Toll-like receptor signaling pathway”, and “NOD-like receptor signaling pathway” were uniquely identified to be significantly enriched in shRIP2 + APEC vs. APEC (Fig. 5B).

### *RIP2* is a critical regulator for MAPK signaling and apoptosis pathway in chicken HD11 macrophages with or without APEC challenge

**Fig. 6:**
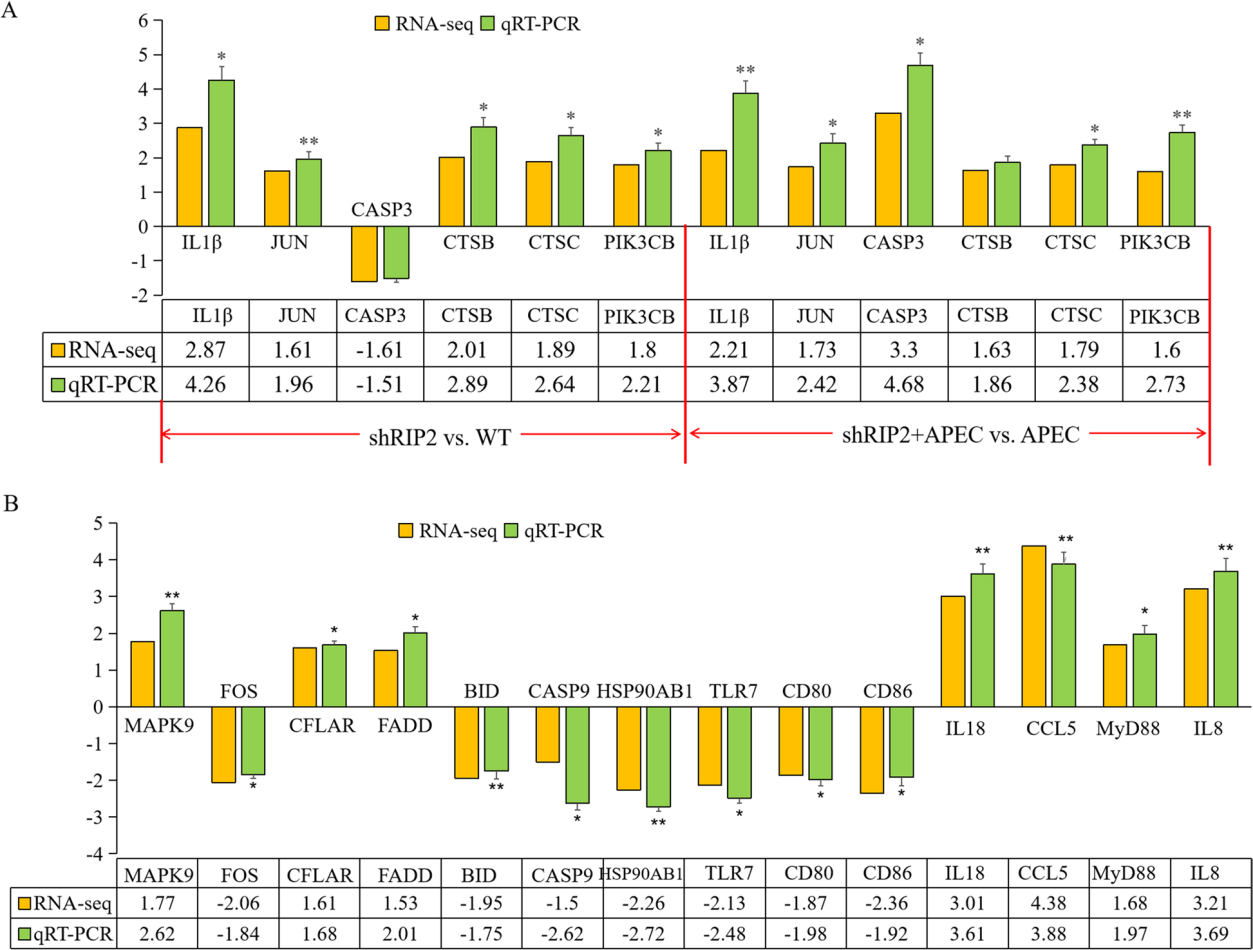
Evaluation and comparison of mRNA expression levels of the selected differentially expressed genes (DEGs) by using RNA-seq and qRT-PCR for different comparisons. **A** The mRNA expression level of the diferentially expressed genes in the comparisons of *RIP2* knockdown HD11 cells (shRIP2) vs. wild type HD11 cells (WT) and *RIP2* knockdown HD11 cells combined with avian pathogenic *E. coli* infection (shRIP2 + APEC) vs. avian pathogenic *E. coli* infection (APEC). (data are shown as mean ± SD; *n* = 3 independent experiments; * *p* < 0.05; ** *p* < 0.01). **B** The mRNA expression level of those candidates uniquely observed in the comparison of *RIP2* knockdown HD11 cells combined with avian pathogenic *E. coli* infection (shRIP2 + APEC) vs. avian pathogenic *E. coli* infection (APEC). (data are shown as mean ± SD; n = 3 independent experiments; * *p* < 0.05; ** *p* < 0.01)

After knockdown of *RIP2*, the MAPK signaling and apoptosis pathway were significantly activated with or without APEC challenge. A total of 39 and 67 DEGs were involved in “MAPK signaling pathway” in shRIP2 vs. WT and shRIP2 + APEC vs. APEC, respectively, as shown in Tables S9 and S10. Nineteen DEGs were commonly involved in the MAPK signaling pathway in both shRIP2 vs. WT and shRIP2 + APEC vs. APEC. To further corroborate the correlation between *RIP2* and MAPK signaling pathway, we used qRT-PCR to detect the expression of the commonly identified DEGs—*IL1β*, *JUN*, and *CASP3* in the two comparisons, as well as the uniquely expressed DEGs—*MAPK9* and *FOS* in shRIP2 + APEC vs. APEC. The qRT-PCR results were in agreement with their transcript abundance changes determined by RNA-seq (Fig. 6).

Moreover, a total of 25 and 48 DEGs were enriched in “apoptosis pathway” in shRIP2 vs. WT (Table S11) and shRIP2 + APEC vs. APEC (Table S12), respectively. Twelve DEGs were enriched in apoptosis pathway in the two comparisons, 3 (*CTSB*, *CTSC*, and *PIK3CB*) of which were selected and used for qRT-PCR validation. Moreover, the uniquely enriched DEGs— *FADD*, *CFLAR*, *BID*, and *CASP9* in the comparison of shRIP2 + APEC vs. APEC were also identified for RNA-seq data reliability. The results showed that knockdown of *RIP2* indeed affected the expression of the the selected genes with or without APEC challenge, which presented 100% consistency between RNA-seq and qRT-PCR (Fig. 6). And, more remarkable, three DEGs (*JUN*, *CASP3*, and *ENSGALG00000031518*) were involved in both MAPK signaling and apoptosis pathway in the two comparisons (Fig. S1), suggesting they had important role in APEC challenge and related with *RIP2*. All these results show that *RIP2* is a critical regulator for MAPK signaling and apoptosis pathway with or without APEC infection.

### Knockdown of *RIP2* impacts the activation of p53 and those genes involved in TLR and NLR signaling pathway upon APEC challenge

**Fig. 7:**
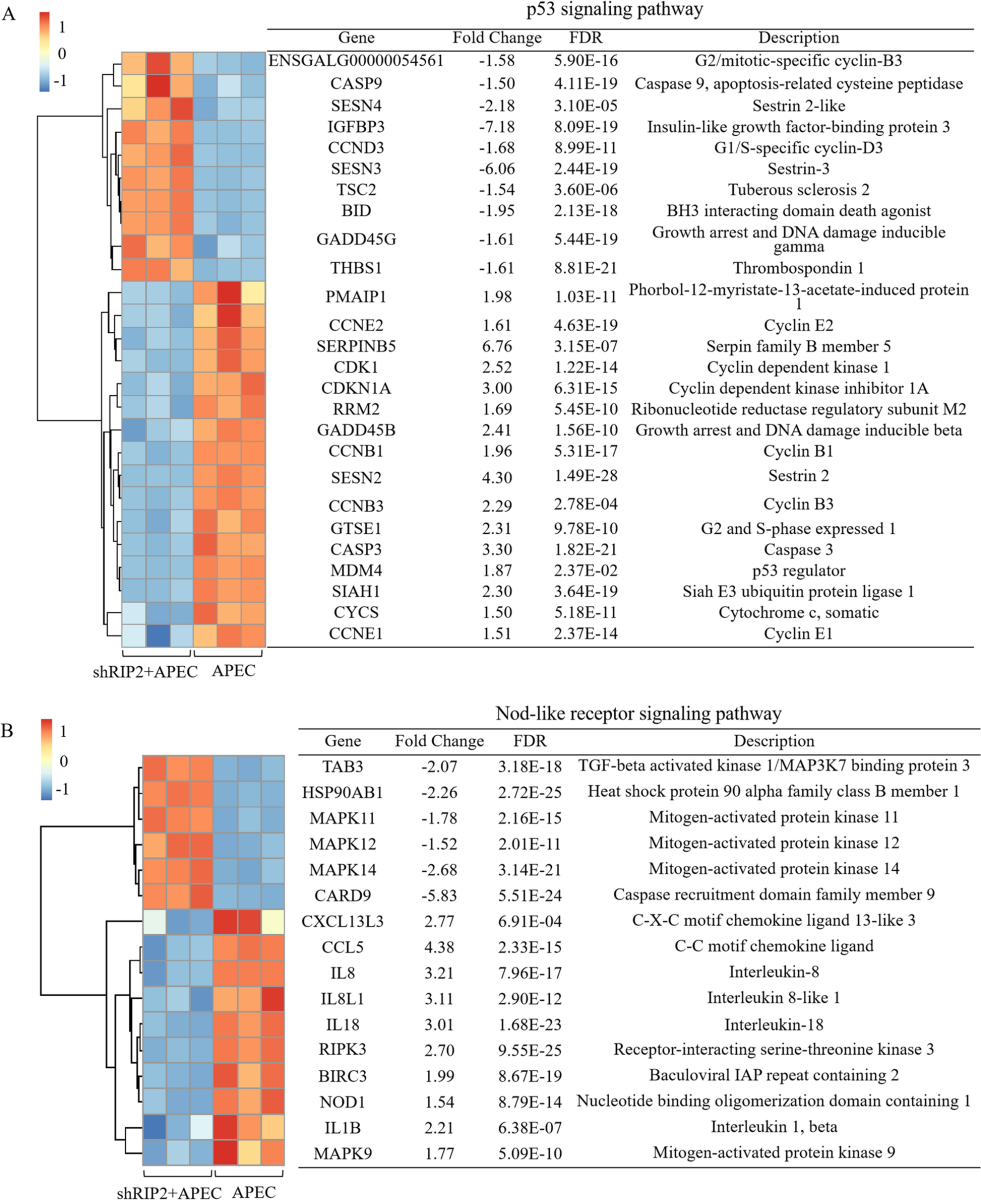
*RIP2* knockdown impacts the activation of p53 and Nod-like receptor (NLR) signaling pathway upon APEC challenge. **A** The gene cluster of p53 signaling pathway in the comparison of *RIP2* knockdown HD11 cells combined with avian pathogenic *E. coli* infection (shRIP2 + APEC) vs. avian pathogenic *E. coli* infection HD11 cells (APEC). **B** The gene cluster of Nod-like receptor signaling pathway in the comparison of *RIP2* knockdown HD11 cells combined with avian pathogenic *E. coli* infection (shRIP2 + APEC) vs. avian pathogenic *E. coli* infection HD11 cells (APEC)

**Fig. 8:**
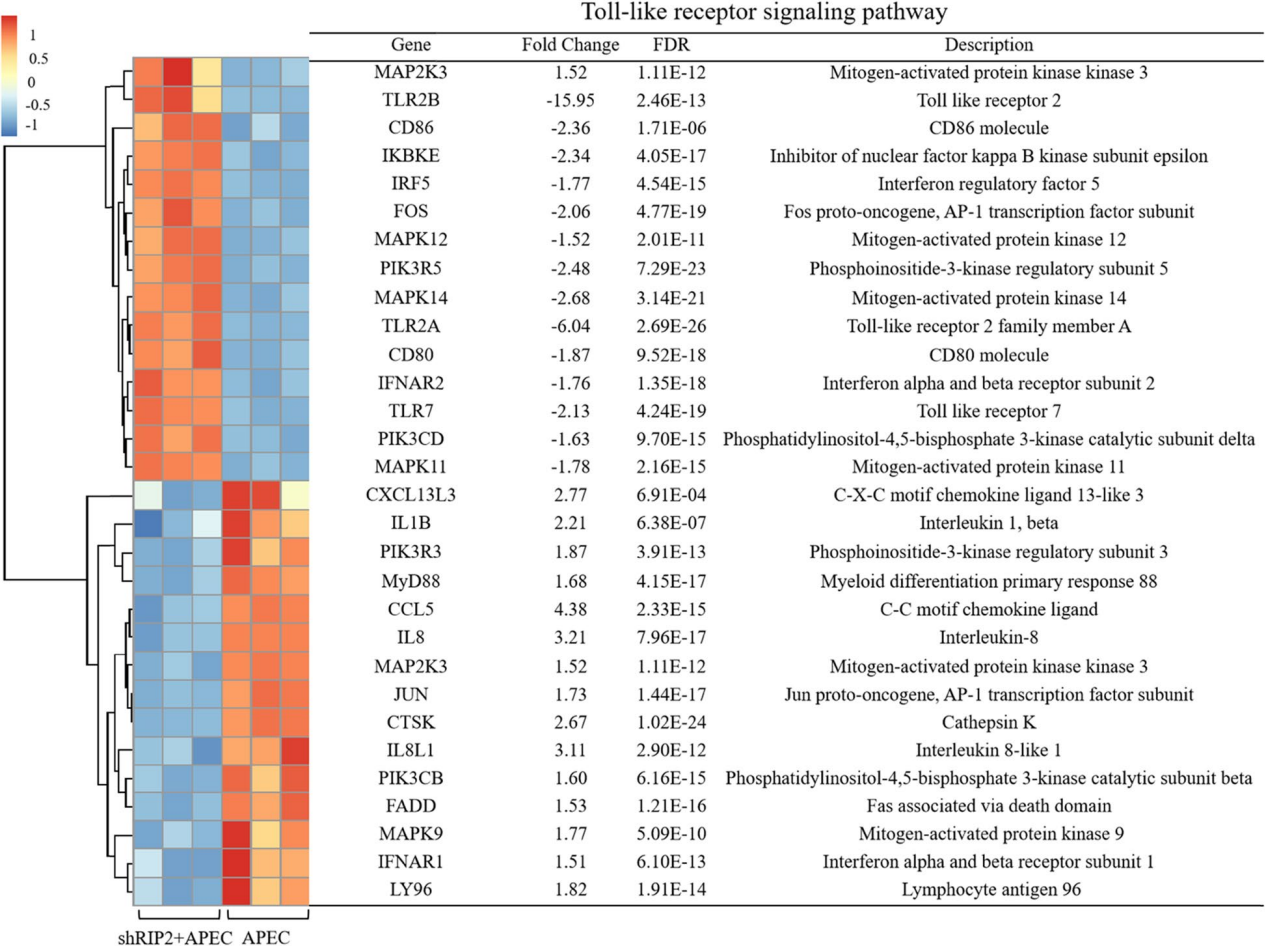
The gene cluster of Toll-like receptor (TLR) signaling pathway and the involved gene cluster in the comparison of *RIP2* knockdown HD11 cells combined with avian pathogenic *E. coli* infection (shRIP2 + APEC) vs. avian pathogenic *E. coli* infection HD11 macrophages (APEC)

Also, we identified the uniquely significantly enriched pathways “p53 signaling pathway”, “Toll-like receptor signaling pathway”, and “NOD-like receptor signaling pathway” in the knockdown of *RIP2* HD11 cells toward APEC challenge (shRIP2 + APEC vs. APEC). A total of 26 DEGs were involved in p53 signaling pathway, of which consisted 16 up-regulated DEGs (FC = 1.5 ~ 6.76) and 9 down-regulated DEGs (FC = − 7.18 ~ − 1.5) (Fig. 7A). Additionally, 16 DEGs (10 up-regulated DEGs with FC of 1.5 ~ 4.38 and 6 down-regulated DEGs with FC of − 5.83 ~ − 1.5) were identified to be involved in NLR signaling pathway, which were the downstream genes of *RIP2* (Fig. 7B). Moreover, there were 30 DEGs enriched in TLR signaling pathway, including 15 up-regulated DEGs (FC = 1.5 ~ 4.38) and 15 down-regulated DEGs (FC = − 15.95 ~ − 1.5) (Fig. 8). Based on the analyses aforementioned, 8 DEGs were randomly selected for validation by qRT-PCR, which includes *CD80*, *CD86*, *IL18*, *CCL5*, *MyD88*, *HSP90AB1*, *TLR7*, and *IL8*. The results showed the significant differences in expression levels of those selected genes determined by qRT-PCR analysis are concordant with the RNA-seq results (Fig. 6), indicating the transcriptome sequencing data are reliable.

### New target genes of *RIP2* upon APEC challenge

**Fig. 9:**
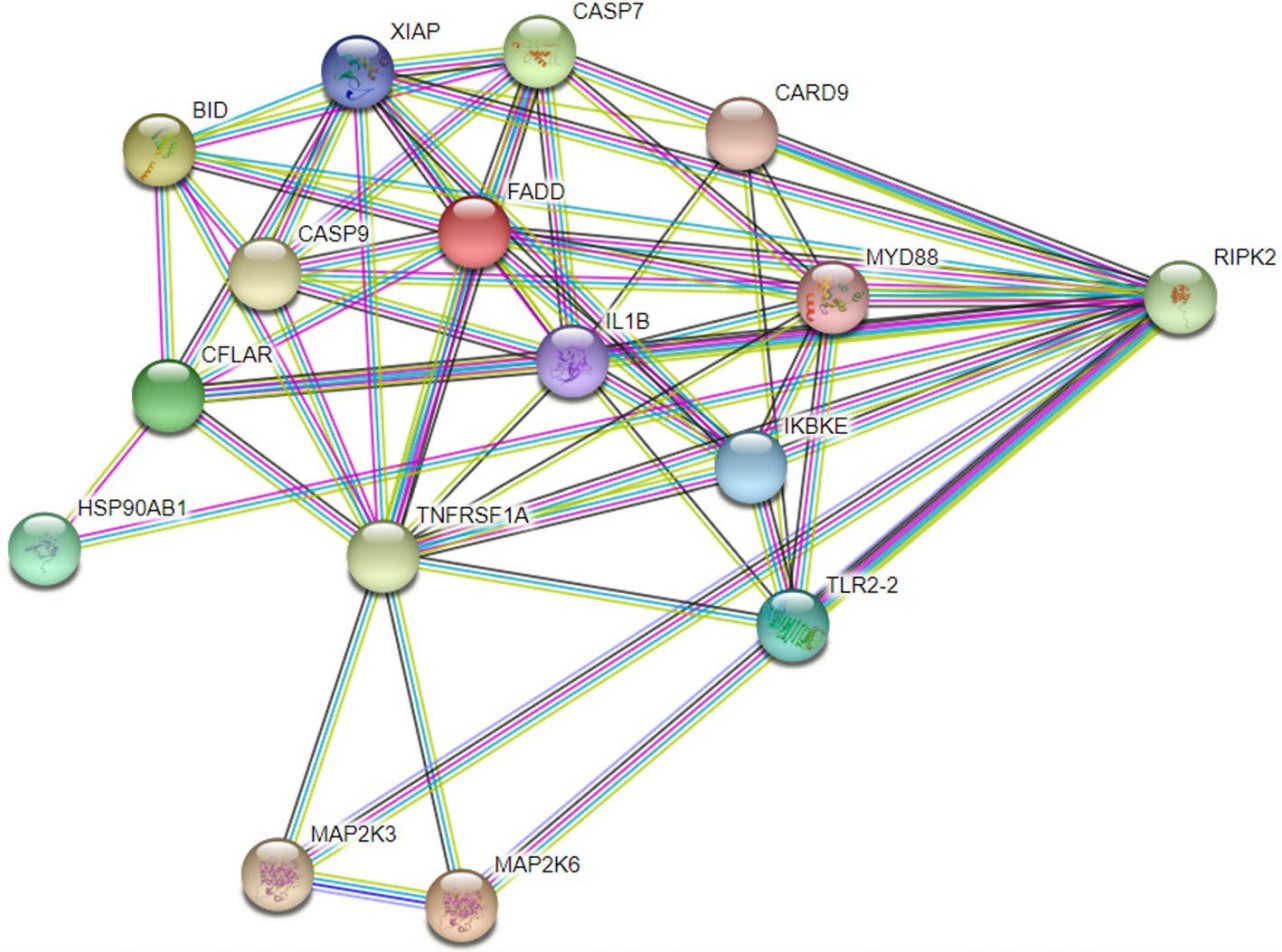
Gene network analysis was used to select the target genes of *RIP2* upon APEC challenge by String. Colored nodes: query proteins and first shell of interactors; white nodes: second shell of interactors; empty nodes: proteins of unknown 3D structure; filled nodes: some 3D structure is known or predicted. The light blue line and purple line represent known interaction. Other color lines indicate predicted interactions

**Fig. 10:**
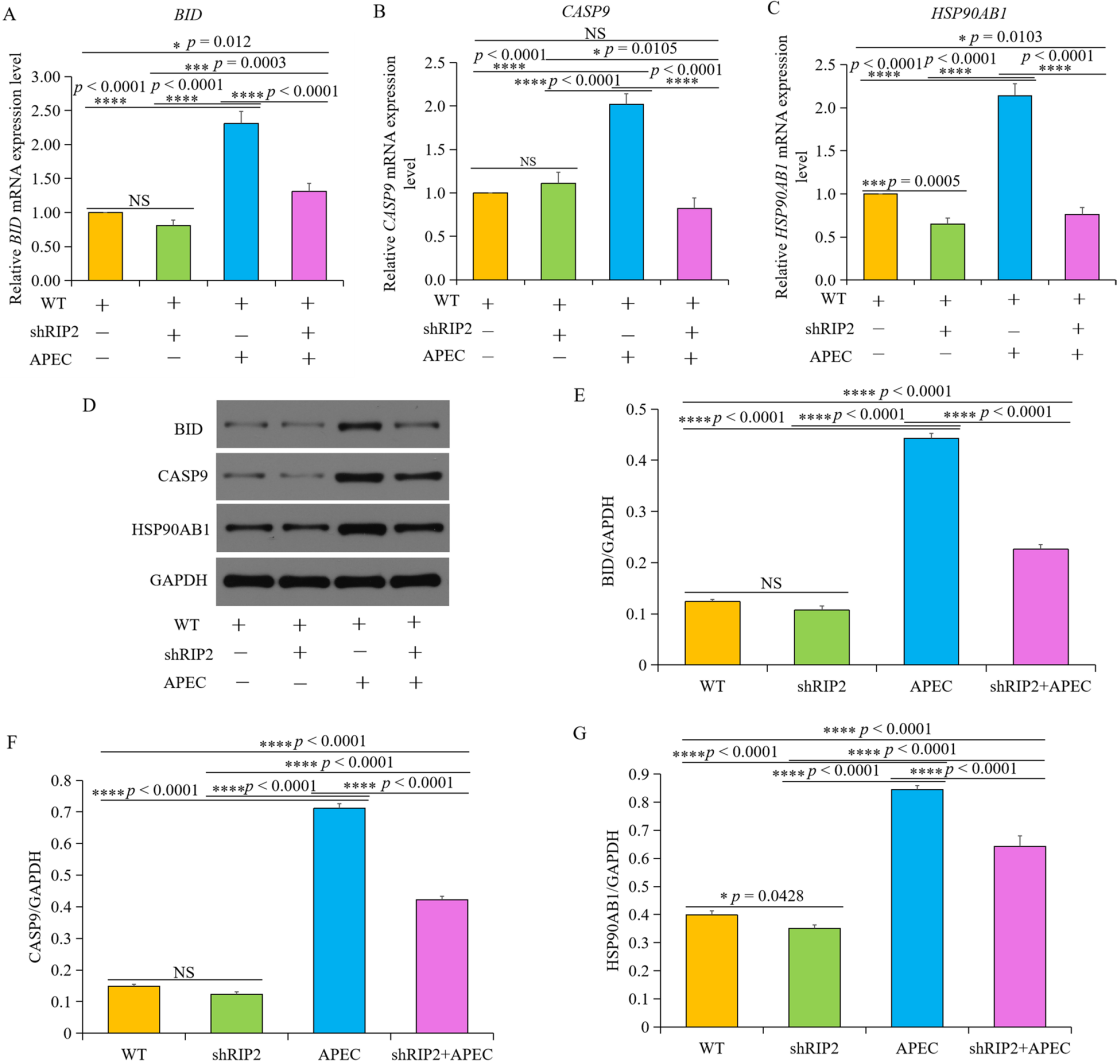
New target genes of *RIP2* upon APEC challenge. **A** The relative *BID* mRNA expression level in the group of wild type HD11 cells (WT), knockdown of *RIP2* HD11 cells (shRIP2), avian pathogenic *E. coli* (APEC), and knockdown of *RIP2* HD11 cells combined with APEC infection (APEC+shRIP2). (data are shown as mean ± SD; *n* = 4 independent experiments; different letters represent a significant difference, *p* < 0.001; same letters indicate no significance, *p* > 0.05). **B** The relative *CASP9* mRNA expression level in group of wild type HD11 cells (WT), knockdown of *RIP2* HD11 cells (shRIP2), avian pathogenic *E. coli* (APEC), and knockdown of *RIP2* HD11 cells combined with APEC infection (APEC+shRIP2). (data are shown as mean ± SD; n = 4 independent experiments; different letters represent a significant difference, *p* < 0.001; same letters indicate no significance, *p* > 0.05). **C** The relative *HSP90AB1* mRNA expression level in the group of wild type HD11 cells (WT), knockdown of *RIP2* HD11 cells (shRIP2), avian pathogenic *E. coli* (APEC), and knockdown of *RIP2* HD11 cells combined with APEC infection (APEC+shRIP2). (data are shown as mean ± SD; n = 4 independent experiments; different letters represent a significant difference, *p* < 0.001; same letters indicate no significance, *p* > 0.05). **D** The protein level of BID, CASP9, and HSP90AB1 in the group of wild type HD11 cells (WT), knockdown of *RIP2* HD11 cells (shRIP2), avian pathogenic *E. coli* (APEC), and knockdown of *RIP2* HD11 cells combined with APEC infection (APEC+shRIP2). **E** Image J software was used for BID gray-level analysis of western blot results (data are shown as mean ± SD; n = 3 independent experiments; different letters represent a significant difference, *p* < 0.05; same letters indicate no significance, *p* > 0.05). **F** Image J software was used for CASP9 gray-level analysis of western blot results (data are shown as mean ± SD; n = 3 independent experiments; different letters represent a significant difference, *p* < 0.05; same letters indicate no significance, *p* > 0.05). **G** Image J software was used for HSP90AB1 gray-level analysis of western blot results (data are shown as mean ± SD; n = 3 independent experiments; different letters represent a significant difference, *p* < 0.05; same letters indicate no significance, *p* > 0.05)

In order to find the new target of *RIP2* toward APEC infection, we collected and analyzed the DEGs enriched in the uniquely significantly changed pathways “p53 signaling pathway”, “Toll-like receptor signaling pathway”, and “NOD-like receptor signaling pathway” in the comparison of shRIP2 + APEC vs. APEC (Fig. 9). Compared to APEC challenge, RNA-seq data showed that *BID*, *CASP9*, and *HSP90AB1* were significantly down-regulated in *RIP2* knockdown HD11 cells toward APEC infection. To evaluate the *RIP2* role in those genes, we studied the expression of above genes in *RIP2* knockdown HD11 cell following APEC challenge. As shown in Fig. 10, the mRNA and protein levels of HSP90AB1 showed remarkable reduction in *RIP2* knockdown HD11 cells (*p* < 0.001) combined APEC challenge compared to the APEC challenge group. Interestingly, compared to the wild type HD11 cells, the mRNA and protein expression level of HSP90AB1 were significantly decreased in the knockdown of *RIP2* HD11 macrophages group (Fig. 10C, D, and G), which is consistent with the results of RNA-seq. These results indicate a strong relationship exists between *RIP2* and *HSP90AB1* with or without APEC challenge. However, interfering *RIP2* did not affect the mRNA and protein expression level of BID and CASP9 (Fig. 10A, B, D, E, and F), which is in agreement with the RNA-seq data. APEC infection had the ability to induce a significant difference in the mRNA and protein expression level of BID and CASP9 between *RIP2* knockdown HD11 cells and wild type HD11 cells, indicating *RIP2* is involved in the regulation of *BID* and *CASP9* in a certain relationship as a result of APEC infection. Taken together, these results showed that the RNA-seq results were considered to be reliable and *RIP2* was involved in the regulation of *BID*, *CASP9*, and *HSP90AB1* in response to APEC infection.

## Discussion

Although *RIP2* has been cloned in chicken [22], the function of *RIP2* was only focused on the activation of NFκB signal pathway [23]. This study first demonstrated the essential genes or pathways, as well as the downstream targets regulated by *RIP2* with or without APEC challenge through transcriptome analysis. We compared the transcriptomes of APEC-induced chicken HD11 cells with knockdown or complete expression of *RIP2*. It was found that in *RIP2* knockdown HD11 cells, many identified DEGs were grouped into the unique functional categories, p53 (*p* = 1.67 × 10^− 3^), TLRs (*p* = 4.14 × 10^− 3^), and NLRs (*p* = 2.54 × 10^− 2^) signaling, toward APEC infection, as expected in cases of impairment of the key regulator for HD11 cells transcriptional program.

p53 signaling pathway is involved in the regulation of a serials normal life activities of cells, such as apoptosis and senescence [24–26]. Normally, p53 has the ability to prevent DNA replication in order to provide time for DNA repair, otherwise it triggers apoptosis, suggesting that p53 plays an important role in monitoring and identifying DNA damage points [27]. Earlier studies have shown that the p53 signaling pathway was significantly suppressed in the comparison of susceptible vs. non-challenged birds at 5 day APEC post-infection in bone marrow and thymus [6, 7], indicating APEC might tend to promote host tumorigenesis. Also, compared to the control group, this pathway was significantly down-regulated in the APEC-induced HD11 cells in the current study, which was consistent with previous studies. However, p53 signaling pathway was significantly up-regulated in the knockdown of *RIP2* HD11 cells combined with APEC infection in comparison to APEC infection in the present study (Table S13). Then, we focused on the p53 related DEGs, since the identification of these genes could help to elucidate the molecular mechanism that underlies the function of *RIP2* in response to APEC infection. Take *SIAH1* as an example, it was up-regulated (Fold changes = 2.3) in shRIP2 + APEC vs. APEC, while down-regulated (Fold changes = − 1.96) in APEC vs. WT. This result was expected, since *SIAH1* was a tumor suppressor and played essential role in regulating cell apoptosis [28]. Another two genes of interest that changed in shRIP2 + APEC vs. APEC were *PMAIP1* and *MDM4*. It has been demonstrated that *PMAIP1* belonged to the pro-apoptotic subfamily and could determine whether a cell commits to apoptosis [29]. Also, this study demonstrated that *MDM4* had the ability to promote cell apoptosis upon genotoxic stress [30]. These two genes were up-regulated in shRIP2 + APEC vs. APEC, indicating knockdown of *RIP2* triggered the activation of p53 signaling pathway and further promote the apoptosis upon APEC challenge. These results coincide with the expectations, since *RIP2* activity correlated with tumor and metastasis et al. [31–33].

Another interesting gene *HSP90AB1* is down-regulated in the comparisons of shRIP2 vs. WT and shRIP2 + APEC vs. APEC. We validated these results finding decreased expression levels of both mRNA and protein in *RIP2* knockdown HD11 cells in any conditions compared to wild type HD11 cells (Fig. 10C, D, and G), suggesting the existence of a positive and cross-regulation between *RIP2* and *HSP90AB1* in chicken HD11 cells with or without APEC challenge. Then, bioinformatics analyses showed that *HSP90AB1* was involved to the GO group of “response to stimulus” and, most importantly, to the KEGG group of “NLR signaling”, closely related to the immune activity of *RIP2*. HSP90AB1 is a member of the large family of HSPs and function as molecular chaperones, which is important to signal transduction, protein folding, apoptosis, inflammation, and cell survival [34, 35]. It has been demonstrated that the expression of *HSP90AB1* mRNA was often expressed on the tissues of heart, liver, brain, and spleen, regulating the heat stress in chickens [36]. In fact, *HSP90AB1* was involved in antiapoptotic and immunostimulatory effects of CpG in both mouse and chicken macrophages [37, 38]. Our transcriptome data showed that the apoptosis and immune related pathways were significantly changed in *RIP2* knockdown HD11 cells with or without APEC challenge, which included the gene of interest *HSP90AB1*. Herein, this study strongly suggests that *HSP90AB1* is a downstream target of RIP2 and positively modulated by *RIP2*.

Moreover, *BID* and *CASP9* were another two important genes that were down-regulated in shRIP2 + APEC vs. APEC, whereas these genes were up-regulated when APEC vs. WT were compared. *BID* is a member of the proapoptotic BCL2 family, which could stimulate mitochondrial outer membrane permeabilization to induce the release of regulators or other cell-death mediators [39]. *CASP9* is the member of cysteine aspartate-specific proteases, serving as an intrinsic initiator of apoptosis [40, 41]. Data obtained by qRT-PCR and western blot showed that the mRNA and protein of BID and CASP9 were both significantly decreased when shRIP2*+* APEC vs APEC were compared (Fig. 10A, B, E and F). It has been demonstrated that *BID* and *CASP9* were both involved in the apoptosis pathway that was downstream of *RIP2* [42, 43]. Accordingly, our data point to *BID* and *CASP9* as the targets of *RIP2*, since *RIP2*-depletion inhibits the expression of *BID* and *CASP9* upon APEC infection. Apoptosis is a safety and important process to remove the damaged DNA, abnormal-proliferated or dedifferentiated cells, which is indispensable for maintaining cellular homeostasis, and normal regulation of the immune system [44, 45]. In the present study, the apoptosis was significantly activated in shRIP2 + APEC vs. APEC, indicating silence of *RIP2* could induce apoptosis to inhibit tumorgenesis. These results were reasonable and expected, since knockdown of *RIP2* could avoid the excessive tissues/cells injury and inflammatory response [42, 46]. In summary, our data suggest that *BID* and *CASP9* could be the two important factors that determine immune and inflammatory response towards HD11 macrophages in the absence of *RIP2* upon APEC challenge, even if the cellular fate in the presence of *BID* and *CASP9* down-regulation is presently unknown. The specific regulation mechanism between *RIP2* and *BID*/*CASP9* involved in response to APEC challenge warrants further study.

## Conclusion

In summary, this study has provided an analysis of the genetic landscape associated with *RIP2* knockdown in HD11 macrophages in the presence or absence of an APEC challenge. In total, 4691 and 2605 DEGs were screened in shRIP2 + APEC vs. APEC and shRIP2 vs. WT, respectively. Functional annotation analysis showed that apoptosis, MAPK, p53, Toll-like receptor, and Nod-like receptor signaling pathways were involved in APEC-induced *RIP2* knockdown HD11 cells. By analyzing the enriched pathway and gene networks, we identified that several key DEGs, like *HSP90AB1*, *BID*, and *CASP9* were targeted by *RIP2* upon APEC infection. As a whole, this study can not only provide data support for constructing gene networks of *RIP2* knockdown associated with APEC challenge but also provide new ideas for improving the immune and inflammatory response.

## Materials and methods

### Cell culture

Chicken HD11 macrophage, an immortalized cell line, was selected as experimental materials as it is similar morphology and function to the in vivo macrophages and helpful to understand the host immune response in the early infection during APEC infection. The chicken HD11 cell line was kindly provided by Dr. Xuming Hu (Yangzhou University). The chicken macrophage-like cell line HD11 was maintained in RPMI1640 (Gibco, Carlsbad, CA, USA) supplemented with 10% fetal bovine serum (FBS, Gibco, Carlsbad, CA, USA) in a humidified incubator with 5% CO_2_ at 37 °C, and cells were passaged before 80–90% confluence.

### Cell transfection

Small hairpin RIP2 (shRIP2) plasmid (Table S1) were synthesized by GenePharma (Shanghai, China). The detailed information for the establishment of shRIP2 HD11 cells can be found in the study of Sun et al. [47]. The Lipofectamine™ 2000 reagent (Invitrogen, Carlsbad, CA, USA) was used for the cell transfection according to the manufacturer’s instructions. After transfection with shRIP2 for 48 h, cells were challenged with or without APEC for 24 h, and collected for further study.

### Quantitative real time PCR (qRT-PCR) analysis

Total RNA was isolated from cells of wild type group (WT), *RIP2* knockdown group (shRIP2), APEC challenge group (APEC), and *RIP2* knockdown with APEC challenge group (shRIP2 + APEC) using Trizol reagent (Invitrogen, Carlsbad, CA, USA) according to the manufacturer’s instructions. Then the RNA was reverse transcribed into cDNA using a Reverse Transcription Kit (Takara, Dalian, China). The One Step SYBR® PrimeScript® PLUS RTRNA PCR Kit (Takara, Dalian, China) was used for cDNA synthesis. qRT-PCR was conducted using a SYBR® Premix Ex Taq™ II kit (Takara, Dalian, China) to evaluate the expression level of *GAPDH*, *RIP2*, *HSP90AB1*, *BID*, and *CASP9*. Primer sequences are displayed in Table S2. qRT-PCR thermal cycling conditions were as follows: denaturation for 3 min at 95 °C, 40 cycles of 10 s at 95 °C, 58 °C for 30 s, and then 72 °C for 30 s. Relative expression of above genes were calculated using the 2^−ΔΔCt^ method and *GAPDH* was utilized as an internal control. The formula of ΔΔCt is (Ct of gene in test group - Ct of *GAPDH* in test group) - (Ct of gene in control group - Ct of *GAPDH* in control group).

### Western blotting

Cells from each of the four groups were lysed on ice using 200 μL RIPA buffer (Beyotime Biotechnology, Shanghai, China) for 30 min. Next, the lysis mixtures were centrifuged and the supernatants were collected. BCA™ Protein Assay Kit (Pierce, Appleton, WI, USA) was used for quantification of proteins. Then, the isolated proteins were subjected to sodium dodecyl sulfate-polyacrylamide gel (SDS-PAGE) and electrophoretically transferred to PVDF membranes. Afterwards, membranes were blocked in 5% BSA for 2 h at room temperature and then probed with the primary antibodies at 4 °C overnight. The primery antibodies included anti-GAPDH (ab181602, Abcam, MA, USA), anti-RIP2 (70R-10,459, Fitzgerald, MA, USA), anti-HSP90AB1 (CBDH1187, Creative Biolabs, NY, USA), anti-BID (AB10002, MilliporeSigma, ON, Canada), and anti-CASP9 (STJ96979, St John’s Laboratory, London, UK) were used at a dilution of 1:1000. Then the membranes were incubated with secondary antibodies tagged with horseradish peroxidas (Sigma-Aldrich, St. Louis, MI, USA) at a 1:10000 dilution at room temperature for 2 h. Then, immunoblots were visualized by enhanced chemiluminescence (ECL kit, Santa Cruz Biotechnology, Dallas, TX, USA). The blots were visualized by using Image Lab™ Software (Bio-Rad, Hercules, CA, USA).

### Nitric oxide (NO) production assay

Chicken HD11 cells from different groups (WT, shRIP2, APEC, and shRIP2 + APEC) were incubated for 24 h, then NO production in the cell supernatant was determined using the Griess reagent kit (Molecular Probes, Carlsbad, CA, USA). The cell supernatant was mixed with Griess reagents and incubated for 30 minutes in the dark, and then measured at 540 nm on a spectrophotometer. The absorbance values were compared to the sodium nitrite standard curve to determine nitrite concentrations (μM).

### Cell viability assay

Cell counting kit-8 (CCK-8) was utilized to determine the viability of cells from different groups (WT, shRIP2, APEC, and shRIP2 + APEC). Cells from each of the four groups were placed in three replicates at a density of 1 × 10^5^ cells per well in a 96-well plate with 100 μL of medium and incubated for 48 h. Then, the cells were incubated for 2 h in 10 μL of CCK-8 solution. Absorbance (optical density, OD) was assessed at 450 nm using a spectrophotometer. The experiment was performed in triplicate.

### RNA-Seq

Total RNA was extracted from HD11 cells of different groups (WT, shRIP2, APEC, and shRIP2 + APEC) using an RNA isolation kit (QIAGEN, Hilden, Germany) according to the manufacturer’s protocol. The quality of RNA was analyzed by agarose gel electrophoresis and a Nanodrop™ OneCspectrophotometer (Thermo Fisher Scientific Inc., MA, USA). The RNA Integrity was confirmed by Qseq (Qseq100, Guangding, Taiwan). A total of 2 μg of RNA was used for stranded RNA sequencing library preparation using Ribo-off rRNA depletion kit (Catalog NO. MRZG12324, Illumina, San Diego, CA, USA) following the manufacturer’s instruction. The library products corresponding to 200–500 bps were enriched, quantified and finally sequenced on NovaSeq 6000 sequencer (Illumina, San Diego, CA, USA) with PE150 sequencing platform.

### Quality control and differentially expressed genes (DEGs) analysis

For quality control, raw data were first filtered by Trimmomatic (version 0.36) (i.e., removing low-quality reads (Q ≤ 10) and repeated and adaptor sequences (10 bp overlap (AGATCGGAAG)). The obtained clean reads were further treated with in-house scripts to eliminate duplication bias introduced in library preparation and sequencing. Deduplicated reads were mapped to the reference genome of the chicken (*Gallus gallus*) from Ensembl (https://asia.ensembl.org/info/data/ftp/index.html) using STRA software (version 2.5.3a) with default parameters. Reads mapped to the exon regions of each gene were counted by featureCounts (Subread-1.5.1; Bioconductor) and then RPKM was calculated. Genes differentially expressed between groups were identified using the edgeR package (version 3.12.1). A *p*-value cutoff of 0.05 and fold-change cutoff of 1.5 were used to judge the statistical significance of gene expression differences.

### GO and KEGG pathway enrichment analyses

GO analysis [48] and KEGG enrichment analysis [49, 50] for DEGs were both implemented by KOBAS software (version: 2.1.1) with a *p*-value cutoff of 0.05 to judge statistically significant enrichment.

### Statistical analyses

Statistical analysis was conducted using a one-way ANOVA and Tukey Honestly Significant differences test (HSD; SAS, 2000; Cary, NC) using JMP statistical software (version 15.2.1, SAS Institute). Data are expressed as the mean ± standard error. The statistical significance was defined at *p* < 0.05. The test results represent the data of four independent experiments.

## Supplementary Information

**Additional file 1: Table S1.** shRNA information for RIP2. **Table S2.** Primers for candidate genes in qRT-PCR validation experiment. **Table S3.** Characteristics of RNA sequencing data before or after quality control. **Table S4.** The reads mapping information for each sample. **Table S5.** The significantly enriched gene ontology (GO) in the comparison of shRIP2 vs. WT with adjusted *p*-value ≤0.05. **Table S6.** The significantly enriched gene ontology (GO) in the comparison of shRIP2 + APEC vs. APEC with adjusted p-value ≤0.05. **Table S**7**.** The significantly enriched pathways in the comparison of shRIP2 vs. WT with adjusted. p_value ≤0.05. **Table S8.** The significantly enriched pathways in the comparison of shRIP2 + APEC vs. APEC with adjusted p-value ≤0.05. **Table S9.** The differentially expressed genes involved in the MAPK signaling pathway in the comparison of shRIP2 vs. WT. **Table S10.** The differentially expressed genes involved in the MAPK signaling pathway in the comparison of shRIP2 + APEC vs. APEC. **Table S11.** The differentially expressed genes involved in the apoptosis pathway in the comparison of shRIP2 vs. WT. **Table S12.** The differentially expressed genes involved in the apoptosis pathway in the comparison of shRIP2 + APEC vs. APEC.

**Additional file 2: Figure S1.** Veen diagram for the MAPK signaling pathway in the contrast of knockdown of RIP2 HD11 cells (shRIP2) vs. wild type HD11 cells (WT) and knockdown of RIP2 combined with avian pathogenic *E. coli* infection HD11 cells (shRIP2 + APEC) vs. avian pathogenic *E. coli* infection HD11 cells (APEC), as well as the apoptosis pathway in the contrast of shRIP2 vs. WT and shRIP2 + APEC vs. APEC. **Figure S2.** Full-length blot of GAPDH protein expression level for Fig. 1B. **Figure S3.** Full-length blot of RIP2 protein expression level for Fig. 1B. **Figure S4.** Full-length blot of BID protein expression level for Fig. 10D. **Figure S5.** Full-length blot of CASP9 protein expression level for Fig. 10D. **Figure S6.** Full-length blot of HSP90AB1 protein expression level for Fig. 10D. **Figure S7.** Full-length blot of GAPDH protein expression level for Fig. 10D.

## Data Availability

All data generated or analyzed for this study are included in this article and its supplementary files. The raw sequence reads were deposited into NCBI SRA database under accession no. PRJNA786725 (https://dataview.ncbi.nlm.nih.gov/object/PRJNA786725).

## Abbreviations

RIP2: Receptor interacting serine/threonine kinase 2
APEC: Avian pathogenic *E. coli*
MAPK: Mitogen-activated protein kinase
TLR: Toll-like receptor
NLR: Nod-like receptor
HSP90AB1: Heat shock protein 90 alpha family class B member 1
BID: BH3 interacting domain death agonist
CASP9: Caspase 9
NFκB: Nuclear factor kappa B
IL6: Interleukin 6
TNFα: Tumor necrosis factor
IP10: C-X-C motif chemokine ligand 10
GO: Gene ontology
KEGG: Kyoto encyclopedia of genes and genomes
NO: Nitric oxide
RPKM: Reads per kilobase per million
CDS: Coding sequence
JUN: Jun proto-oncogene, AP-1 transcription factor subunit
CASP3: Caspase 3
MAP K9: Mitogen-activated protein kinase 9
FOS: Fos proto-oncogene, AP-1 transcription factor subunit
CTSB: Cathepsin B
CTSC: Cathepsin C
PIK3CB: Phosphatidylinositol-4,5-bisphosphate 3-kinase catalytic subunit beta
FADD: Fas associated via death domain
CFLAR: CASP8 and FADD like apoptosis regulator
CD80: CD80 molecule
CD86: CD86 molecule
IL18: Interleukin 18
CCL5: C-C motif chemokine ligand
MyD88: Myeloid differentiation primary response 88
TLR7: Toll like receptor 7
IL8: Interleukin 8
SIAH1: Siah E3 ubiquitin protein ligase 1
PMAIP1: Phorbol-12-myristate-13-acetate-induced protein 1
MDM4: p53 regulator
