# Supplementary material for: Gene expression profiling of RIP2-knockdown in HD11 macrophages — elucidation of potential pathways (gene network) when challenged with avian pathogenic E.coli (APEC)
Source: BMC Genomics. 2022 May 2;23:341. doi: 10.1186/s12864-022-08595-5 (PMC9063279; doi:10.1186/s12864-022-08595-5)
Supplement: Supplementary file 1 — Additional file 1: Table S1. shRNA information for RIP2. Table S2. Primers for candidate genes in qRT-PCR validation experiment. Table S3. Characteristics of RNA sequencing data before or after quality control. Table S4. The reads mapping information for each sample. Table S5. The significantly enriched gene ontology (GO) in the comparison of shRIP2 vs. WT with adjusted p-value ≤0.05. Table S6. The significantly enriched gene ontology (GO) in the comparison of shRIP2 + APEC vs. APEC with adjusted p-value ≤0.05. Table S7. The significantly enriched pathways in the comparison of shRIP2 vs. WT with adjusted. p_value ≤0.05. Table S8. The significantly enriched pathways in the comparison of shRIP2 + APEC vs. APEC with adjusted p-value ≤0.05. Table S9. The differentially expressed genes involved in the MAPK signaling pathway in the comparison of shRIP2 vs. WT. Table S10. The differentially expressed genes involved in the MAPK signaling pathway in the comparison of shRIP2 + APEC vs. APEC. Table S11. The differentially expressed genes involved in the apoptosis pathway in the comparison of shRIP2 vs. WT. Table S12. The differentially expressed genes involved in the apoptosis pathway in the comparison of shRIP2 + APEC vs. APEC. [file 12864_2022_8595_MOESM1_ESM.pdf]

Legends for the supplementary tables

Table S1 shRNA information for RIP2

Table S2 Primers for candidate genes in qRT-PCR validation experiment

Table S3 Characteristics of RNA sequencing data before or after quality control

Table S4 The reads mapping information for each sample

Table S5 The significantly enriched gene ontology (GO) in the comparison of shRIP2 vs. WT with adjusted p-value ≤ 0.05

Table S6 The significantly enriched gene ontology (GO) in the comparison of shRIP2+APEC vs. APEC with adjusted p-value ≤ 0.05

Table S7 The significantly enriched pathways in the comparison of shRIP2 vs. WT with adjusted p_value ≤0.05

Table S8 The significantly enriched pathways in the comparison of shRIP2+APEC vs. APEC with adjusted p-value ≤ 0.05

Table S9 The differentially expressed genes involved in the MAPK signaling pathway in the comparison of shRIP2 vs. WT

Table S10 The differentially expressed genes involved in the MAPK signaling pathway in the comparison of shRIP2+APEC vs. APEC

Table S11 The differentially expressed genes involved in the apoptosis pathway in the comparison of shRIP2 vs. WT

Table S12 The differentially expressed genes involved in the apoptosis pathway in the comparison of shRIP2+APEC vs. APEC

Table S1 shRNA information for RIP2

**ID**	**5'**	**stem**	**loop**	**stem**	**3'**
NC-F	GATCC	GACGAAAGTCAAGACTTCTATTCAA	TTCAAGAGA	TTGAATAGAAGTCTTGACTTTCGTC	TTTTTTG
NC-R	AATTCAAAAAA	GACGAAAGTCAAGACTTCTATTCAA	TCTCTTGAA	TTGAATAGAAGTCTTGACTTTCGTC	G
shRNA1-F	GATCC	ACAGTGATAGAAACCACCTTCTAAA	TTCAAGAGA	TTTAGAAGGTGGTTTCTATCACTGT	TTTTTTG
shRNA1-R	AATTCAAAAAA	ACAGTGATAGAAACCACCTTCTAAA	TCTCTTGAA	TTTAGAAGGTGGTTTCTATCACTGT	G
shRNA2-F	GATCC	GACTTAAGTGAAACCAGCTTATCAA	TTCAAGAGA	TTGATAAGCTGGTTTCACTTAAGTC	TTTTTTG
shRNA2-R	AATTCAAAAAA	GACTTAAGTGAAACCAGCTTATCAA	TCTCTTGAA	TTGATAAGCTGGTTTCACTTAAGTC	G
shRNA3-F	GATCC	ACAAACAGCTAGGACTTCAACCTTA	TTCAAGAGA	TAAGGTTGAAGTCCTAGCTGTTTGT	TTTTTTG
shRNA3-R	AATTCAAAAAA	ACAAACAGCTAGGACTTCAACCTTA	TCTCTTGAA	TAAGGTTGAAGTCCTAGCTGTTTGT	G

Table S2 Primers for candidate genes in qRT-PCR validation experiment

Gene Name	Gene ID	Sequence (5'-3')	
GAPDH	374193	sense	GTCGGAGTCAACGGATTTGG
		antisense	GTTCTCAGCCTTGACAGTGCC
RIP2	420215	sense	CTCGAACCAGTCCTGAGAACG
		antisense	AAGCGGATGTTTCCTCTTGG
MAPK9	395983	sense	TTACAGAGCGCCAGAGGTTA
		antisense	TCTCCCATGATGCAACCAAC
FOS	396512	sense	GCCGACATGATGTACCAGGG
		antisense	GACGGGTAGTAGGTGAGGCT
CASP3	395476	sense	CGCTCAGGGGAAGATGTATCA
		antisense	CCAGAGTCCACAGACTTGCTA
CTSB	396329	sense	CTGCTCACTCTGCTCCATTAT
		antisense	GCCCTTCCCTAGGATCAATAAC
HSP90AB1	396188	sense	GGTGGTTGTCATTACCAAGCAC
		antisense	CCACATAAAGTGTGATGGGGTAG
BID	395236	sense	TTACGATAGTGATGGGGAACTTC
		antisense	GCACCGTGTTATCTCCTCTCTAG
TLR7	418638	sense	AGTGGTTGCTGCTGTTGTCT
		antisense	CGTCCTTGCATGATGTACCATT
CASP9	426970	sense	CCAATATTCCTCTCCATCCTG
		antisense	CTCTTGTCAACAGCTGAACCCT
IL1β	395196	sense	GTGGCCATGACCAAACTGCT
		antisense	GAAGGACTGTGAGCGGGTGT
JUN	424673	sense	CCTCCCCTGTCCCCTATTGA
		antisense	CCTTTTCCGGCATTTGGACG
FADD	423146	sense	TTGGGAAAAGGAAGTTGGAGT
		antisense	CGTTCTCGCATATAACTTCGAT
CFLAR	424080	sense	TACTGCTGGCCTTAGAGAGCTT
		antisense	TTTCATTCGAGGAGCAAAATCT
PIK3CB	424826	sense	GCCCACTGGAATCTACATCAA
		antisense	AGGAGATGGAAGAGTGGATAGG
CD80	768950	sense	CAGCAAGCCGAACATAGAAAGA
		antisense	AGCAAACTGGTGGACCTGAGA
CD86	427944	sense	TGTAGGGATTGTGGATGAGGG
		antisense	CGCTGGAAGAGCAGGAAAGAT
IL18	395312	sense	CGTGGCAGCTTTTGAAGATGTA
		antisense	CTGAATGCAACAGGCATCCC
CCL5	417465	sense	CTGCCCCAGAATCATGTGAA
		antisense	CAGCTCCAGGAAGTTGATGTA
MyD88	420420	sense	ACTTACCAGAGGTTGCTGCC
		antisense	GTCACCTTGGTACCATGCCA

IL8	396495	sense	CCCCACTGCAAGAATGTTGAAA
		antisense	GTGCCTTTACGATCAGCTGTAC
CTSC	419014	sense	CAGTCCCCAGCAGGTTGTA
		antisense	GGCTCCATAGAAACCACCA

Table S3 Characteristics of RNA sequencing data before or after quality control

Sample	Raw bases (G)	Raw reads	Clean reads	Barcoded reads	UID reads	Raw Q20 (%)	Clean Q20 (%)	Raw Q30 (%)	Clean Q30 (%)	Clean unique Percentage	UID unique Percentage	Clean GC (%)	UID GC (%)	Clean to raw read ratio (%)	Barcoded to clean read ratio (%)	UID to barcoded ratio (%)	Dedup ratio (%)
WT 1	11.98	79898294	77586290	74670816	55232248	99.95	100	97.3	99.2	42.34	63.28	51.52	51.23	97.11	96.24	73.97	26.03
WT 2	11.71	78068340	75776170	72942218	54688864	99.95	100	97.4	99.3	43.05	64.08	52.42	52.18	97.06	96.26	74.98	25.02
WT 3	11.7	77977654	75414976	72679902	55110018	99.95	100	96.95	99.1	44.42	64.04	52.74	52.48	96.71	96.37	75.83	24.17
shRIP2 1	13.5	89995934	87645300	84359058	60443170	99.95	100	97.25	99.15	39.63	55.31	52.29	52.03	97.39	96.25	71.65	28.35
shRIP2 2	12.21	81387452	79241946	76218648	55397950	99.95	100	97.25	99.15	40.75	61.7	52.79	52.56	97.36	96.18	72.68	27.32
shRIP2 3	10.72	71438338	69393540	66598758	49039816	99.95	100	97.25	99.2	42.09	67.6	52.87	52.68	97.14	95.97	73.63	26.37
APEC 1	12.74	84964948	82496206	78927676	57401552	99.95	100	97.05	99.1	42.21	61.38	52.25	51.97	97.09	95.67	72.73	27.27
APEC 2	11.59	77283432	75293620	72401464	53680588	99.95	100	97.3	99.25	42.9	65.27	52.72	52.48	97.43	96.16	74.14	25.86
APEC 3	12.2	81346714	79136728	75983904	55185622	99.95	100	97.15	99.2	42.23	63.56	52.53	52.28	97.28	96.02	72.63	27.37
shRIP2+APEC 1	13.16	87741412	85491752	82265564	59235236	99.95	100	97.3	99.2	40.52	58.07	51.6	51.3	97.44	96.23	72	28
shRIP2+APEC 2	14.31	95374638	92952070	89504242	64145408	99.95	100	97.35	99.2	39.87	55.4	52.43	52.14	97.46	96.29	71.67	28.33
shRIP2+APEC 3	12.66	84413690	82247886	78867018	57758260	99.95	100	97.25	99.15	40.85	59.34	51.86	51.58	97.43	95.89	73.23	26.77

Table S4 The reads mapping information for each sample

Sample	total_reads	total mapped (%)	nonunique (%)	unique (%)	unmapped reads (%)	read 1 (%)	read 2 (%)	reads map plus (%)	reads map minus (%)
WT_1	55232248	51589617 (93.40)	4512427 (8.75)	47077190 (91.25)	3642631 (6.60)	23518840 (49.96)	23558350 (50.04)	23537675 (50.00)	23539515 (50.00)
WT_2	54688864	50974341 (93.21)	5295239 (10.39)	45679102 (89.61)	3714523 (6.79)	22817542 (49.95)	22861560 (50.05)	22837832 (50.00)	22841270 (50.00)
WT_3	55110018	51275004 (93.04)	5560473 (10.84)	45714531 (89.16)	3835014 (6.96)	22836558 (49.95)	22877973 (50.05)	22855568 (50.00)	22858963 (50.00)
shRIP2_1	60443170	56229954 (93.03)	5568658 (9.90)	50661296 (90.10)	4213216 (6.97)	25306048 (49.95)	25355248 (50.05)	25328762 (50.00)	25332534 (50.00)
shRIP2_2	55397950	51447994 (92.87)	5360132 (10.42)	46087862 (89.58)	3949956 (7.13)	23022406 (49.95)	23065456 (50.05)	23042034 (50.00)	23045828 (50.00)
shRIP2_3	49039816	45515322 (92.81)	4758486 (10.45)	40756836 (89.55)	3524494 (7.19)	20360130 (49.96)	20396706 (50.04)	20377195 (50.00)	20379641 (50.00)
APEC_1	57401552	53393248 (93.02)	5117638 (9.58)	48275610 (90.42)	4008304 (6.98)	24114829 (49.95)	24160781 (50.05)	24135701 (50.00)	24139909 (50.00)
APEC_2	53680588	49970523 (93.09)	4942818 (9.89)	45027705 (90.11)	3710065 (6.91)	22493130 (49.95)	22534575 (50.05)	22511978 (50.00)	22515727 (50.00)
APEC_3	55185622	51327487 (93.01)	4854328 (9.46)	46473159 (90.54)	3858135 (6.99)	23215343 (49.95)	23257816 (50.05)	23234623 (50.00)	23238536 (50.00)
APEC + shRIP2_1	59235236	55738361 (94.10)	5303951 (9.52)	50434410 (90.48)	3496875 (5.90)	25195607 (49.96)	25238803 (50.04)	25216171 (50.00)	25218239 (50.00)
APEC + shRIP2_2	64145408	60160874 (93.79)	6248070 (10.39)	53912804 (89.61)	3984534 (6.21)	26929080 (49.95)	26983724 (50.05)	26954773 (50.00)	26958031 (50.00)

APEC + shRIP2_3	57758260	54295939 (94.01)	5233836 (9.64)	49062103 (90.36)	3462321 (5.99)	24508335 (49.95)	24553768 (50.05)	24530019 (50.00)	24532084 (50.00)

Table S5 The significantly enriched gene ontology (GO) in the comparison of shRIP2 vs. WT with adjusted *p* -value ≤ 0.05

Term	Database	ID	Input number	Background number	Rich factor	*p* value	Corrected *p* value
single-organism process	Undef	GO:0044699	482	3374	1.05	3.56E-23	1.64E-19
cellular process	biological process	GO:0009987	543	4001	1	7.74E-22	1.78E-18
single-organism cellular process	Undef	GO:0044763	417	2902	1.05	3.04E-20	4.68E-17
cell	cellular component	GO:0005623	559	4280	0.96	3.07E-19	2.86E-16
cell part	cellular component	GO:0044464	557	4261	0.96	3.11E-19	2.86E-16
biological regulation	biological process	GO:0065007	398	2799	1.04	1.38E-18	1.06E-15
binding	molecular function	GO:0005488	445	3243	1.01	2.41E-18	1.59E-15
metabolic process	biological process	GO:0008152	416	3005	1.02	1.15E-17	6.61E-15
regulation of biological process	biological process	GO:0050789	373	2619	1.04	1.76E-17	9.03E-15
cytoplasm	cellular component	GO:0005737	374	2632	1.04	2.17E-17	1.00E-14
membrane	cellular component	GO:0016020	338	2310	1.07	2.52E-17	1.06E-14
regulation of cellular process	biological process	GO:0050794	353	2483	1.04	2.00E-16	7.70E-14
catalytic activity	molecular function	GO:0003824	268	1736	1.13	3.02E-16	1.07E-13
organic substance metabolic process	biological process	GO:0071704	388	2817	1.01	3.95E-16	1.30E-13
single-organism metabolic process	Undef	GO:0044710	179	1017	1.29	1.67E-15	5.12E-13
intracellular	cellular component	GO:0005622	486	3785	0.94	2.61E-15	7.52E-13
membrane part	cellular component	GO:0044425	261	1743	1.1	1.87E-14	5.06E-12
primary metabolic process	biological process	GO:0044238	363	2671	1	2.48E-14	6.34E-12
intracellular part	cellular component	GO:0044424	464	3636	0.94	3.53E-14	8.57E-12
cellular metabolic process	biological process	GO:0044237	364	2694	0.99	4.71E-14	1.09E-11
signal transduction	biological process	GO:0007165	203	1272	1.17	1.11E-13	2.44E-11
single organism signaling	Undef	GO:0044700	212	1364	1.14	3.03E-13	6.34E-11
signaling	biological process	GO:0023052	212	1367	1.14	3.65E-13	7.32E-11
cell communication	biological process	GO:0007154	212	1375	1.13	6.01E-13	1.16E-10
response to stimulus	biological process	GO:0050896	266	1859	1.05	9.98E-13	1.84E-10

intrinsic component of membrane	cellular component	GO:0031224	222	1483	1.1	2.15E-12	3.82E-10
integral component of membrane	cellular component	GO:0016021	218	1455	1.1	3.22E-12	5.50E-10
phosphate-containing compound metabolic process	biological process	GO:0006796	137	782	1.29	5.31E-12	8.75E-10
phosphorus metabolic process	biological process	GO:0006793	137	783	1.28	5.75E-12	9.14E-10
organelle	cellular component	GO:0043226	428	3422	0.92	6.89E-12	1.06E-09
extracellular region part	cellular component	GO:0044421	157	949	1.21	7.44E-12	1.11E-09
cytoplasmic part	cellular component	GO:0044444	253	1785	1.04	9.26E-12	1.33E-09
membrane-bounded organelle	cellular component	GO:0043227	389	3072	0.93	1.92E-11	2.69E-09
oxidation-reduction process	biological process	GO:0055114	67	282	1.74	4.91E-11	6.66E-09
cellular response to stimulus	biological process	GO:0051716	223	1553	1.05	6.33E-11	8.34E-09
extracellular region	cellular component	GO:0005576	168	1081	1.14	1.05E-10	1.34E-08
cell periphery	cellular component	GO:0071944	158	1005	1.15	1.85E-10	2.31E-08
plasma membrane	cellular component	GO:0005886	154	975	1.16	2.37E-10	2.88E-08
oxidoreductase activity	molecular function	GO:0016491	57	235	1.78	6.94E-10	8.21E-08
membrane-bounded vesicle	Undef	GO:0031988	132	822	1.18	1.93E-09	2.21E-07
intracellular membrane-bounded organelle	cellular component	GO:0043231	342	2742	0.92	1.97E-09	2.21E-07
intracellular organelle	cellular component	GO:0043229	379	3106	0.9	2.31E-09	2.54E-07
regulation of catalytic activity	biological process	GO:0050790	91	495	1.35	2.72E-09	2.91E-07
phosphorylation	biological process	GO:0016310	99	564	1.29	4.51E-09	4.72E-07
intracellular signal transduction	biological process	GO:0035556	105	613	1.26	4.84E-09	4.96E-07
vesicle	cellular component	GO:0031982	133	847	1.15	5.68E-09	5.70E-07
extracellular vesicle	cellular component	GO:1903561	111	670	1.22	8.80E-09	8.64E-07
extracellular organelle	cellular component	GO:0043230	111	671	1.21	9.45E-09	9.08E-07
organic cyclic compound binding	molecular function	GO:0097159	224	1672	0.98	1.26E-08	1.18E-06
positive regulation of biological process	biological process	GO:0048518	169	1179	1.05	1.70E-08	1.57E-06

single-organism developmental process	Undef	GO:0044767	190	1369	1.02	1.83E-08	1.65E-06
extracellular exosome	cellular component	GO:0070062	109	667	1.2	2.26E-08	2.01E-06
hydrolase activity	molecular function	GO:0016787	114	710	1.18	2.49E-08	2.17E-06
heterocyclic compound binding	molecular function	GO:1901363	220	1652	0.98	2.64E-08	2.25E-06
regulation of molecular function	biological process	GO:0065009	101	606	1.22	3.17E-08	2.66E-06
protein binding	molecular function	GO:0005515	206	1528	0.99	3.32E-08	2.73E-06
macromolecule metabolic process	biological process	GO:0043170	294	2359	0.91	3.51E-08	2.77E-06
regulation of metabolic process	biological process	GO:0019222	199	1465	1	3.52E-08	2.77E-06
developmental process	biological process	GO:0032502	190	1383	1.01	3.55E-08	2.77E-06
cell surface receptor signaling pathway	biological process	GO:0007166	99	596	1.22	5.00E-08	3.85E-06
response to chemical	biological process	GO:0042221	118	756	1.15	5.53E-08	4.18E-06
cell proliferation	biological process	GO:0008283	76	416	1.34	6.80E-08	5.05E-06
regulation of response to stimulus	biological process	GO:0048583	119	769	1.14	7.32E-08	5.36E-06
protein metabolic process	biological process	GO:0019538	192	1418	0.99	7.63E-08	5.50E-06
regulation of primary metabolic process	biological process	GO:0080090	186	1375	0.99	1.31E-07	9.28E-06
ion binding	molecular function	GO:0043167	140	962	1.07	1.37E-07	9.59E-06
anatomical structure development	biological process	GO:0048856	176	1285	1	1.39E-07	9.59E-06
small molecule binding	molecular function	GO:0036094	118	773	1.12	1.57E-07	1.06E-05
macromolecule modification	biological process	GO:0043412	145	1010	1.05	1.68E-07	1.12E-05
small molecule metabolic process	biological process	GO:0044281	79	452	1.28	1.88E-07	1.24E-05
single-multicellular organism	Undef	GO:0044707	184	1369	0.99	2.26E-07	1.47E-05
regulation of cellular metabolic process	biological process	GO:0031323	186	1388	0.98	2.32E-07	1.47E-05
positive regulation of catalytic	biological process	GO:0043085	57	285	1.47	2.33E-07	1.47E-05
negative regulation of biological process	biological process	GO:0048519	153	1099	1.02	4.01E-07	2.50E-05

cation binding	molecular function	GO:0043169	133	921	1.06	4.14E-07	2.54E-05
localization	biological process	GO:0051179	187	1413	0.97	4.57E-07	2.77E-05
cellular developmental process	biological process	GO:0048869	137	959	1.05	4.72E-07	2.83E-05
positive regulation of molecular function	biological process	GO:0044093	64	346	1.36	4.95E-07	2.92E-05
protein phosphorylation	biological process	GO:0006468	79	465	1.25	5.08E-07	2.93E-05
regulation of protein metabolic process	biological process	GO:0051246	98	622	1.16	5.08E-07	2.93E-05
protein modification process	biological process	GO:0036211	137	963	1.04	5.79E-07	3.25E-05
cellular protein modification process	biological process	GO:0006464	137	963	1.04	5.79E-07	3.25E-05
nucleotide binding	molecular function	GO:0000166	109	720	1.11	6.61E-07	3.63E-05
nucleoside phosphate binding	molecular function	GO:1901265	109	720	1.11	6.61E-07	3.63E-05
regulation of macromolecule metabolic process	biological process	GO:0060255	183	1385	0.97	6.70E-07	3.63E-05
nucleosome	cellular component	GO:0000786	19	46	3.03	6.86E-07	3.68E-05
cellular protein metabolic process	biological process	GO:0044267	171	1276	0.98	7.26E-07	3.85E-05
response to external stimulus	biological process	GO:0009605	73	424	1.26	8.84E-07	4.63E-05
metal ion binding	molecular function	GO:0046872	129	901	1.05	9.22E-07	4.78E-05
regulation of cellular protein metabolic process	biological process	GO:0032268	91	575	1.16	1.09E-06	5.59E-05
DNA packaging complex	cellular component	GO:0044815	19	48	2.9	1.15E-06	5.77E-05
positive regulation of cellular	biological process	GO:0048522	147	1067	1.01	1.15E-06	5.77E-05
regulation of cell proliferation	biological process	GO:0042127	61	333	1.34	1.20E-06	5.94E-05
organelle part	cellular component	GO:0044422	243	1970	0.9	1.27E-06	6.22E-05
response to organic substance	biological process	GO:0010033	89	561	1.16	1.31E-06	6.34E-05
negative regulation of cellular process	biological process	GO:0048523	141	1016	1.02	1.34E-06	6.45E-05
cellular response to chemical	biological process	GO:0070887	85	532	1.17	1.75E-06	8.33E-05

cellular macromolecule metabolic process	biological process	GO:0044260	264	2184	0.89	1.80E-06	8.45E-05
system development	biological process	GO:0048731	139	1007	1.01	2.06E-06	9.61E-05
carbohydrate derivative binding	molecular function	GO:0097367	100	663	1.11	2.19E-06	0.000100952
cellular component organization	biological process	GO:0016043	188	1462	0.94	2.27E-06	0.000102841
organonitrogen compound metabolic process	biological process	GO:1901564	85	536	1.16	2.28E-06	0.000102841
multicellular organismal process	biological process	GO:0032501	192	1500	0.94	2.30E-06	0.000102899
biosynthetic process	biological process	GO:0009058	193	1511	0.94	2.44E-06	0.000108171
intracellular organelle part	cellular component	GO:0044446	236	1925	0.9	2.73E-06	0.000119657
multicellular organism development	biological process	GO:0007275	152	1136	0.98	3.29E-06	0.000143214
locomotion	biological process	GO:0040011	61	347	1.29	3.82E-06	0.000164713
cell differentiation	biological process	GO:0030154	122	868	1.03	4.05E-06	0.000172756
organophosphate metabolic process	biological process	GO:0019637	47	240	1.44	4.20E-06	0.000177689
enzyme linked receptor protein signaling pathway	biological process	GO:0007167	46	234	1.44	4.85E-06	0.000203165
immune system process	biological process	GO:0002376	76	475	1.17	5.80E-06	0.000239499
cellular biosynthetic process	biological process	GO:0044249	184	1449	0.93	5.82E-06	0.000239499
organic substance biosynthetic process	biological process	GO:1901576	187	1482	0.93	6.89E-06	0.000280951
blood vessel development	biological process	GO:0001568	34	152	1.64	8.10E-06	0.000327485
response to stress	biological process	GO:0006950	104	723	1.06	8.70E-06	0.000348902
NAD binding	molecular function	GO:0051287	11	18	4.48	9.65E-06	0.000383464
organelle membrane	cellular component	GO:0031090	79	510	1.14	1.08E-05	0.000425537
angiogenesis	biological process	GO:0001525	27	107	1.85	1.09E-05	0.000425537
regulation of signal transduction	biological process	GO:0009966	89	598	1.09	1.23E-05	0.000476194
cellular component organization or biogenesis	biological process	GO:0071840	189	1523	0.91	1.50E-05	0.00057812
regulation of hydrolase activity	biological process	GO:0051336	48	262	1.34	1.57E-05	0.000596307

blood vessel morphogenesis	biological process	GO:0048514	30	130	1.69	1.62E-05	0.000610965
positive regulation of hydrolase activity	biological process	GO:0051345	34	158	1.58	1.63E-05	0.000611376
regulation of protein modification process	biological process	GO:0031399	65	400	1.19	1.75E-05	0.000650316
organic substance catabolic process	biological process	GO:1901575	67	418	1.18	1.92E-05	0.000708791
protein-DNA complex	cellular component	GO:0032993	20	67	2.19	2.00E-05	0.000730434
vasculature development	biological process	GO:0001944	34	160	1.56	2.04E-05	0.000741117
cellular response to organic substance	biological process	GO:0071310	71	453	1.15	2.11E-05	0.000761254
regulation of protein	biological process	GO:0001932	55	322	1.25	2.28E-05	0.000813492
catabolic process	biological process	GO:0009056	67	422	1.16	2.50E-05	0.000887017
movement of cell or subcellular component	biological process	GO:0006928	63	389	1.19	2.56E-05	0.000899447
extrinsic component of membrane	cellular component	GO:0019898	21	75	2.05	2.79E-05	0.000972684
molecular function regulator	molecular function	GO:0098772	49	277	1.3	2.85E-05	0.000972684
regulation of phosphorus metabolic process	biological process	GO:0051174	64	399	1.18	2.86E-05	0.000972684
regulation of phosphate metabolic process	biological process	GO:0019220	64	399	1.18	2.86E-05	0.000972684
lipid metabolic process	biological process	GO:0006629	50	285	1.29	2.87E-05	0.000972684
transmembrane receptor protein tyrosine kinase signaling pathway	biological process	GO:0007169	32	149	1.58	2.95E-05	0.000992134
cellular lipid metabolic process	biological process	GO:0044255	40	209	1.4	3.31E-05	0.001105617
nitrogen compound metabolic	biological process	GO:0006807	203	1683	0.88	3.44E-05	0.001140102
positive regulation of GTPase	biological process	GO:0043547	24	96	1.83	3.73E-05	0.001227479
immune response	biological process	GO:0006955	42	227	1.36	4.23E-05	0.00138365
endomembrane system	cellular component	GO:0012505	114	848	0.99	4.63E-05	0.001502778

response to oxygen-containing compound	biological process	GO:1901700	43	236	1.34	4.72E-05	0.001521335
single-organism biosynthetic	Undef	GO:0044711	54	326	1.22	5.55E-05	0.001776706
regulation of phosphorylation	biological process	GO:0042325	56	343	1.2	5.78E-05	0.001836068
cell motility	biological process	GO:0048870	51	303	1.23	6.17E-05	0.001947028
regulation of intracellular signal transduction	biological process	GO:1902531	58	361	1.18	6.37E-05	0.001996675
regulation of signaling	biological process	GO:0023051	93	665	1.03	6.49E-05	0.002020061
localization of cell	biological process	GO:0051674	51	304	1.23	6.64E-05	0.002053357
regulation of cell communication	biological process	GO:0010646	92	657	1.03	6.80E-05	0.002088339
extrinsic component of plasma membrane	cellular component	GO:0019897	14	39	2.63	6.87E-05	0.002097675
nervous system development	biological process	GO:0007399	73	490	1.09	6.98E-05	0.002116857
chemotaxis	biological process	GO:0006935	27	122	1.62	7.74E-05	0.002331436
regulation of kinase activity	biological process	GO:0043549	36	188	1.4	7.90E-05	0.002366335
regulation of biological quality	biological process	GO:0065008	108	806	0.98	8.15E-05	0.002424248
taxis	biological process	GO:0042330	27	123	1.61	8.71E-05	0.002568161
purine ribonucleoside binding	molecular function	GO:0032550	82	573	1.05	8.75E-05	0.002568161
extracellular space	cellular component	GO:0005615	51	308	1.21	8.86E-05	0.002586374
programmed cell death	biological process	GO:0012501	60	383	1.15	8.97E-05	0.002600386
purine nucleoside binding	molecular function	GO:0001883	82	574	1.05	9.21E-05	0.002653107
positive regulation of metabolic process	biological process	GO:0009893	96	700	1.01	9.47E-05	0.002711197
cell development	biological process	GO:0048468	72	487	1.08	9.61E-05	0.002724523
response to cytokine	biological process	GO:0034097	28	131	1.57	9.67E-05	0.002724523
ribonucleoside binding	molecular function	GO:0032549	82	575	1.05	9.69E-05	0.002724523
ribonucleotide binding	molecular function	GO:0032553	83	585	1.04	0.00010261	0.002866971
reactive oxygen species metabolic process	biological process	GO:0072593	14	41	2.51	0.00010646	0.002956568

nucleoside binding	molecular function	GO:0001882	82	577	1.04	0.00010732	0.002962501
establishment of localization	biological process	GO:0051234	143	1141	0.92	0.00011106	0.003047452
carbohydrate derivative metabolic process	biological process	GO:1901135	48	287	1.23	0.00011408	0.003095132
plasma membrane part	cellular component	GO:0044459	78	543	1.05	0.00011548	0.003095132
cell adhesion	biological process	GO:0007155	52	320	1.19	0.00011548	0.003095132
biological adhesion	biological process	GO:0022610	52	320	1.19	0.00011548	0.003095132
purine ribonucleoside triphosphate binding	molecular function	GO:0035639	81	571	1.04	0.0001242	0.003297052
monocarboxylic acid metabolic process	biological process	GO:0032787	26	119	1.6	0.00012444	0.003297052
cytosol	cellular component	GO:0005829	67	449	1.09	0.00013013	0.003428055
transport	biological process	GO:0006810	139	1108	0.92	0.00013279	0.003478278
purine ribonucleotide binding	molecular function	GO:0032555	82	582	1.03	0.00013793	0.003592467
regulation of GTPase activity	biological process	GO:0043087	25	113	1.62	0.00014061	0.003641655
positive regulation of response to stimulus	biological process	GO:0048584	60	391	1.13	0.00014755	0.00379995
lysosome	cellular component	GO:0005764	22	93	1.74	0.00015308	0.003898884
lytic vacuole	cellular component	GO:0000323	22	93	1.74	0.00015308	0.003898884
enzyme regulator activity	molecular function	GO:0030234	39	219	1.31	0.00015718	0.003981197
inflammatory response	biological process	GO:0006954	24	107	1.65	0.00015836	0.003989163
purine nucleotide binding	molecular function	GO:0017076	82	585	1.03	0.00015994	0.004007294
regulation of protein kinase activity	biological process	GO:0045859	33	174	1.39	0.0001792	0.004465573
receptor binding	molecular function	GO:0005102	55	353	1.14	0.00019439	0.004818058
cytoskeletal protein binding	molecular function	GO:0008092	38	214	1.3	0.00019925	0.004912079
transferase activity, transferring phosphorus-containing groups	molecular function	GO:0016772	46	279	1.21	0.0002081	0.005102962
generation of precursor metabolites and energy	biological process	GO:0006091	20	82	1.79	0.00021227	0.005165364

regulation of transferase activity	biological process	GO:0051338	37	207	1.31	0.00021418	0.005165364
ribonucleoside monophosphate metabolic process	biological process	GO:0009161	18	69	1.91	0.00021498	0.005165364
single organism cell adhesion	Undef	GO:0098602	33	176	1.38	0.00021525	0.005165364
cell death	biological process	GO:0008219	61	406	1.1	0.00021625	0.005165364
purine-containing compound metabolic process	biological process	GO:0072521	26	124	1.54	0.00021909	0.005206217
nucleobase-containing small molecule metabolic process	biological process	GO:0055086	32	169	1.39	0.00022831	0.00539745
cell-cell adhesion	biological process	GO:0098609	36	200	1.32	0.00022991	0.005407535
fatty acid metabolic process	biological process	GO:0006631	19	76	1.83	0.00023142	0.005415537
organic acid metabolic process	biological process	GO:0006082	38	216	1.29	0.00023451	0.005460051
positive regulation of phosphate metabolic process	biological process	GO:0045937	41	241	1.25	0.00025359	0.005845246
positive regulation of phosphorus metabolic process	biological process	GO:0010562	41	241	1.25	0.00025359	0.005845246
mitochondrion	cellular component	GO:0005739	66	453	1.07	0.0002637	0.006048103
cell migration	biological process	GO:0016477	46	283	1.19	0.00027543	0.006244028
anatomical structure morphogenesis	biological process	GO:0009653	89	660	0.99	0.00027631	0.006244028
positive regulation of macromolecule metabolic process	biological process	GO:0010604	89	660	0.99	0.00027631	0.006244028
positive regulation of cell differentiation	biological process	GO:0045597	35	195	1.32	0.0002919	0.006564282
regulation of developmental process	biological process	GO:0050793	73	517	1.04	0.00029482	0.006597756
apoptotic process	biological process	GO:0006915	57	377	1.11	0.00029708	0.006616156
cell surface	cellular component	GO:0009986	30	157	1.4	0.00030794	0.006824937
ribose phosphate metabolic process	biological process	GO:0019693	25	120	1.53	0.0003118	0.006877438
regulation of angiogenesis	biological process	GO:0045765	13	41	2.33	0.00033826	0.007425648
regulation of localization	biological process	GO:0032879	73	520	1.03	0.00034226	0.007477777

cell chemotaxis	biological process	GO:0060326	14	47	2.19	0.00034516	0.007505694
small GTPase mediated signal transduction	biological process	GO:0007264	29	151	1.41	0.00035742	0.007735752
purine ribonucleoside monophosphate metabolic process	biological process	GO:0009167	17	66	1.89	0.00036096	0.007739739
purine nucleoside monophosphate metabolic process	biological process	GO:0009126	17	66	1.89	0.00036096	0.007739739
proteolysis	biological process	GO:0006508	60	407	1.08	0.00037482	0.007999539
negative regulation of cellular metabolic process	biological process	GO:0031324	76	549	1.02	0.00037887	0.008048746
cellular response to organonitrogen compound	biological process	GO:0071417	18	73	1.81	0.0003852	0.008145754
nucleotide metabolic process	biological process	GO:0009117	29	152	1.4	0.0003928	0.008268502
regulation of cellular component organization	biological process	GO:0051128	73	523	1.02	0.0003965	0.008308383
cofactor metabolic process	biological process	GO:0051186	19	80	1.74	0.00040163	0.008377855
cellular nitrogen compound metabolic process	biological process	GO:0034641	184	1580	0.85	0.0004065	0.008441295
coenzyme binding	molecular function	GO:0050662	14	48	2.14	0.00041252	0.00852798
wound healing	biological process	GO:0042060	21	94	1.64	0.00041455	0.008531664
regulation of programmed cell death	biological process	GO:0043067	50	323	1.14	0.00042161	0.008638298
chromatin	cellular component	GO:0000785	27	138	1.44	0.00043617	0.008897106
nucleoside monophosphate metabolic process	biological process	GO:0009123	18	74	1.78	0.00044265	0.008989569
positive regulation of immune system process	biological process	GO:0002684	29	154	1.38	0.00047295	0.009467354
nucleoside phosphate metabolic process	biological process	GO:0006753	29	154	1.38	0.00047295	0.009467354

positive regulation of protein metabolic process	biological process	GO:0051247	52	342	1.12	0.00047501	0.009467354
vacuolar membrane	cellular component	GO:0005774	25	124	1.48	0.00047627	0.009467354
positive regulation of cellular metabolic process	biological process	GO:0031325	86	645	0.98	0.00047645	0.009467354
whole membrane	cellular component	GO:0098805	44	275	1.17	0.0004948	0.009789861
cellular catabolic process	biological process	GO:0044248	53	352	1.1	0.00051653	0.010176118
regulation of cell death	biological process	GO:0010941	51	335	1.12	0.00052173	0.010234713
ribonucleotide metabolic process	biological process	GO:0009259	24	118	1.49	0.00054868	0.010717934
cysteine-type peptidase activity	molecular function	GO:0008234	15	56	1.97	0.00055615	0.010817979
negative regulation of metabolic process	biological process	GO:0009892	80	594	0.99	0.00056592	0.010936784
response to wounding	biological process	GO:0009611	23	111	1.52	0.000567	0.010936784
transferase activity	molecular function	GO:0016740	90	687	0.96	0.00059043	0.011318864
G-protein coupled receptor signaling pathway	biological process	GO:0007186	42	261	1.18	0.00059172	0.011318864
response to lipid	biological process	GO:0033993	28	149	1.38	0.0006033	0.011492627
response to organonitrogen compound	biological process	GO:0010243	23	112	1.51	0.00063123	0.011964209
mitochondrial envelope	cellular component	GO:0005740	30	165	1.33	0.00063325	0.011964209
carbohydrate metabolic process	biological process	GO:0005975	32	181	1.3	0.0006491	0.012213714
negative regulation of protein metabolic process	biological process	GO:0051248	40	246	1.19	0.00065959	0.012360609
positive regulation of multicellular organismal process	biological process	GO:0051240	47	305	1.13	0.00067122	0.012527598
cellular modified amino acid metabolic process	biological process	GO:0006575	11	33	2.45	0.000677	0.01258463
bounding membrane of organelle	cellular component	GO:0098588	50	331	1.11	0.00068559	0.01269308
mitochondrial part	cellular component	GO:0044429	36	214	1.23	0.00069779	0.012787686

organonitrogen compound catabolic process	biological process	GO:1901565	16	64	1.83	0.00070023	0.012787686
purine ribonucleotide metabolic process	biological process	GO:0009150	23	113	1.49	0.00070169	0.012787686
signal transduction by protein phosphorylation	biological process	GO:0023014	33	190	1.27	0.00070658	0.012787686
oxidoreductase activity, acting on paired donors, with incorporation or reduction of molecular oxygen	molecular function	GO:0016705	13	45	2.12	0.00070723	0.012787686
oxidoreductase activity, acting on CH-OH group of donors	molecular function	GO:0016614	12	39	2.26	0.00070734	0.012787686
positive regulation of kinase activity	biological process	GO:0033674	22	106	1.52	0.00072502	0.013056071
NADH dehydrogenase (quinone) activity	molecular function	GO:0050136	7	13	3.95	0.00074417	0.013297011
NADH dehydrogenase (ubiquinone) activity	molecular function	GO:0008137	7	13	3.95	0.00074417	0.013297011
single organismal cell-cell adhesion	Undef	GO:0016337	30	167	1.32	0.00075129	0.01337232
regulation of cell differentiation	biological process	GO:0045595	53	359	1.08	0.00077199	0.013687889
positive regulation of apoptotic process	biological process	GO:0043065	23	114	1.48	0.00077888	0.013757168
mitochondrial membrane	cellular component	GO:0031966	28	152	1.35	0.00079051	0.01390943
protein complex assembly	Undef	GO:0006461	47	308	1.12	0.00080671	0.014086781
protein complex biogenesis	biological process	GO:0070271	47	308	1.12	0.00080671	0.014086781
positive regulation of developmental process	biological process	GO:0051094	42	266	1.16	0.00082586	0.014350446
regulation of apoptotic process	biological process	GO:0042981	48	317	1.11	0.00082803	0.014350446
regulation of vasculature development	biological process	GO:1901342	13	46	2.07	0.00083979	0.014454115
transition metal ion binding	molecular function	GO:0046914	55	378	1.07	0.00084028	0.014454115

positive regulation of programmed cell death	biological process	GO:0043068	23	115	1.47	0.00086331	0.01474027
purine nucleotide metabolic process	biological process	GO:0006163	23	115	1.47	0.00086331	0.01474027
response to endogenous stimulus	biological process	GO:0009719	49	327	1.1	0.00089884	0.015290176
regulation of biosynthetic process	biological process	GO:0009889	118	963	0.9	0.0009051	0.015340111
neurogenesis	biological process	GO:0022008	51	345	1.08	0.00093435	0.015777873
negative regulation of cell proliferation	biological process	GO:0008285	25	131	1.4	0.0009507	0.015878735
single-organism localization	Undef	GO:1902578	87	671	0.95	0.00095418	0.015878735
positive regulation of cell death	biological process	GO:0010942	23	116	1.45	0.00095555	0.015878735
cofactor binding	molecular function	GO:0048037	17	73	1.71	0.00095622	0.015878735
carboxylic acid metabolic process	biological process	GO:0019752	34	202	1.23	0.00095755	0.015878735
cell activation	biological process	GO:0001775	33	194	1.25	0.00096461	0.015885565
endosome membrane	cellular component	GO:0010008	19	87	1.6	0.00096485	0.015885565
negative regulation of catalytic activity	biological process	GO:0043086	31	178	1.28	0.0009685	0.015888867
positive regulation of protein modification process	biological process	GO:0031401	40	252	1.16	0.00099109	0.016087721
circulatory system development	biological process	GO:0072359	40	252	1.16	0.00099109	0.016087721
cardiovascular system development	biological process	GO:0072358	40	252	1.16	0.00099109	0.016087721
small molecule biosynthetic process	biological process	GO:0044283	24	124	1.42	0.0010042	0.016243449
energy derivation by oxidation of organic compounds	biological process	GO:0015980	15	60	1.83	0.00101556	0.016322595
positive regulation of protein phosphorylation	biological process	GO:0001934	35	211	1.22	0.00101935	0.016322595
cellular respiration	biological process	GO:0045333	12	41	2.15	0.00101972	0.016322595
defense response	biological process	GO:0006952	41	261	1.15	0.00103104	0.016381014
oxoacid metabolic process	biological process	GO:0043436	34	203	1.23	0.00103152	0.016381014
NADH dehydrogenase activity	molecular function	GO:0003954	7	14	3.67	0.00103758	0.016381014

oxidoreductase activity, acting on NAD(P)H, quinone or similar compound as acceptor	molecular function	GO:0016655	7	14	3.67	0.00103758	0.016381014
vacuolar part	cellular component	GO:0044437	25	132	1.39	0.00104423	0.016429667
regulation of G-protein coupled receptor protein signaling pathway	biological process	GO:0008277	9	24	2.75	0.00105253	0.016498005
negative regulation of cellular protein metabolic process	biological process	GO:0032269	37	228	1.19	0.00105913	0.016498005
nucleus	cellular component	GO:0005634	194	1716	0.83	0.00105931	0.016498005
kinase activity	molecular function	GO:0016301	38	237	1.18	0.00111094	0.017243865
regulation of response to external stimulus	biological process	GO:0032101	29	164	1.3	0.00112887	0.017463427
MAPK cascade	biological process	GO:0000165	30	172	1.28	0.00113424	0.017487802
respiratory chain complex	cellular component	GO:0098803	10	30	2.45	0.00117659	0.01802512
protein heterodimerization activity	molecular function	GO:0046982	26	141	1.35	0.00117691	0.01802512
hydrolase activity, acting on carbon-nitrogen (but not peptide) bonds	molecular function	GO:0016810	12	42	2.1	0.00121442	0.018538029
regulation of cellular biosynthetic process	biological process	GO:0031326	116	954	0.89	0.00126791	0.019290634
positive regulation of transferase activity	biological process	GO:0051347	23	119	1.42	0.00128508	0.019487576
chromatin silencing	biological process	GO:0006342	9	25	2.64	0.00132824	0.020075998
mitochondrial inner membrane	cellular component	GO:0005743	22	112	1.44	0.00135251	0.020376008
phosphotransferase activity, alcohol group as acceptor	molecular function	GO:0016773	34	207	1.21	0.00137972	0.02067074
regulation of multicellular organismal process	biological process	GO:0051239	76	578	0.96	0.00138104	0.02067074
cellular response to nitrogen compound	biological process	GO:1901699	18	83	1.59	0.00138713	0.020694671

chromatin assembly	biological process	GO:0031497	12	43	2.05	0.00143891	0.021397972
protein dimerization activity	molecular function	GO:0046983	47	318	1.08	0.00144955	0.021486831
respiratory chain	cellular component	GO:0070469	11	37	2.18	0.00147098	0.021734739
protein complex subunit	Undef	GO:0071822	49	336	1.07	0.00149472	0.022014958
neuron projection development	biological process	GO:0031175	32	192	1.22	0.00152605	0.022404751
anatomical structure formation involved in morphogenesis	biological process	GO:0048646	44	293	1.1	0.00153333	0.022414887
regulation of lipid metabolic process	biological process	GO:0019216	15	63	1.75	0.00154133	0.022414887
coenzyme metabolic process	biological process	GO:0006732	15	63	1.75	0.00154133	0.022414887
leukocyte activation	biological process	GO:0045321	28	160	1.28	0.00155678	0.022568484
chromatin assembly or disassembly	biological process	GO:0006333	13	50	1.91	0.00159647	0.023071301
cellular nitrogen compound biosynthetic process	biological process	GO:0044271	137	1166	0.86	0.00160736	0.023156075
regulation of reactive oxygen species metabolic process	biological process	GO:2000377	9	26	2.54	0.00165923	0.023754869
negative regulation of gene expression, epigenetic	biological process	GO:0045814	9	26	2.54	0.00165923	0.023754869
positive regulation of	biological process	GO:0042327	35	218	1.18	0.00166839	0.02381199
cellular component morphogenesis	biological process	GO:0032989	50	347	1.06	0.00168507	0.023975907
nucleoside metabolic process	biological process	GO:0009116	18	85	1.55	0.00174506	0.024753028
purine ribonucleoside metabolic process	biological process	GO:0046128	16	71	1.65	0.00178285	0.025211471
NAD+ binding	molecular function	GO:0070403	5	7	5.24	0.00180521	0.025449667
cellular response to oxygen-containing compound	biological process	GO:1901701	27	154	1.29	0.00182456	0.025644035
dioxygenase activity	molecular function	GO:0051213	8	21	2.79	0.00183286	0.02568231
negative regulation of macromolecule metabolic process	biological process	GO:0010605	72	548	0.96	0.00184705	0.025802744
GTPase activator activity	molecular function	GO:0005096	13	51	1.87	0.00185524	0.025838843

single-organism catabolic process	Undef	GO:0044712	28	163	1.26	0.00197688	0.027387648
regulation of MAPK cascade	biological process	GO:0043408	27	155	1.28	0.00197833	0.027387648
nucleosome organization	biological process	GO:0034728	12	45	1.96	0.00199089	0.027399861
envelope	cellular component	GO:0031975	40	263	1.12	0.0019911	0.027399861
purine nucleoside metabolic process	biological process	GO:0042278	16	72	1.63	0.00201597	0.027659575
regulation of response to stress	biological process	GO:0080134	38	247	1.13	0.00212781	0.029021357
microtubule cytoskeleton	cellular component	GO:0015630	38	247	1.13	0.00212781	0.029021357
response to organic cyclic	biological process	GO:0014070	27	156	1.27	0.00214306	0.029119522
GTPase regulator activity	molecular function	GO:0030695	13	52	1.83	0.00214764	0.029119522
cell morphogenesis	biological process	GO:0000902	46	317	1.06	0.00222659	0.030013357
positive regulation of cellular protein metabolic process	biological process	GO:0032270	46	317	1.06	0.00222659	0.030013357
nucleoside-triphosphatase regulator activity	molecular function	GO:0060589	14	59	1.74	0.00223926	0.030096161
iron ion homeostasis	biological process	GO:0055072	6	12	3.67	0.00238721	0.031991399
nuclear chromatin	cellular component	GO:0000790	18	88	1.5	0.002426	0.03232335
glycosyl compound metabolic	biological process	GO:1901657	18	88	1.5	0.002426	0.03232335
nucleosome assembly	biological process	GO:0006334	11	40	2.02	0.00246917	0.032709455
carbohydrate binding	molecular function	GO:0030246	11	40	2.02	0.00246917	0.032709455
response to alcohol	biological process	GO:0097305	7	17	3.02	0.00248193	0.032784176
ribonucleoside metabolic process	biological process	GO:0009119	17	81	1.54	0.00250462	0.032989474
cellular aromatic compound metabolic process	biological process	GO:0006725	161	1421	0.83	0.0025503	0.033486142
protein localization to cell periphery	biological process	GO:1990778	14	60	1.71	0.00255686	0.033486142
endosomal part	cellular component	GO:0044440	19	96	1.45	0.00258095	0.03370583
adenyl ribonucleotide binding	molecular function	GO:0032559	59	437	0.99	0.00265558	0.034582576
cell projection organization	biological process	GO:0030030	43	294	1.07	0.00266653	0.034627369
response to nitrogen compound	biological process	GO:1901698	24	135	1.3	0.00270731	0.035058098
ATP binding	molecular function	GO:0005524	58	429	0.99	0.00279259	0.036061128
adenyl nucleotide binding	molecular function	GO:0030554	59	439	0.99	0.00290519	0.037016549

oxidoreductase activity, acting on NAD(P)H	molecular function	GO:0016651	8	23	2.55	0.00290672	0.037016549
pigment granule	cellular component	GO:0048770	8	23	2.55	0.00290672	0.037016549
melanosome	cellular component	GO:0042470	8	23	2.55	0.00290672	0.037016549
electron transport chain	biological process	GO:0022900	8	23	2.55	0.00290672	0.037016549
regulation of cell adhesion	biological process	GO:0030155	24	136	1.29	0.00294258	0.037290073
response to hormone	biological process	GO:0009725	26	152	1.25	0.00294438	0.037290073
organic cyclic compound metabolic process	biological process	GO:1901360	164	1457	0.83	0.00295403	0.037309779
oxidoreductase complex	cellular component	GO:1990204	10	35	2.1	0.00303848	0.038271551
vacuole	cellular component	GO:0005773	38	253	1.1	0.00306374	0.038388744
generation of neurons	biological process	GO:0048699	46	323	1.04	0.00306444	0.038388744
DNA packaging	biological process	GO:0006323	12	48	1.83	0.0031333	0.039144982
positive regulation of protein kinase activity	biological process	GO:0045860	19	98	1.42	0.00315045	0.039252925
cellular response to endogenous stimulus	biological process	GO:0071495	41	280	1.07	0.00323673	0.040219208
regulation of cell-substrate adhesion	biological process	GO:0010810	11	42	1.92	0.00339642	0.041977146
myeloid leukocyte activation	biological process	GO:0002274	11	42	1.92	0.00339642	0.041977146
lymphocyte activation	biological process	GO:0046649	24	138	1.28	0.00346516	0.042630839
regulation of vesicle-mediated transport	biological process	GO:0060627	17	84	1.48	0.0034678	0.042630839
regulation of cellular component movement	biological process	GO:0051270	28	171	1.2	0.00359991	0.043621421
positive regulation of angiogenesis	biological process	GO:0045766	8	24	2.45	0.00360062	0.043621421
signal transducer activity	molecular function	GO:0004871	51	371	1.01	0.00360349	0.043621421
growth factor activity	molecular function	GO:0008083	12	49	1.8	0.00361462	0.043621421
lytic vacuole membrane	cellular component	GO:0098852	12	49	1.8	0.00361462	0.043621421

response to molecule of bacterial origin	biological process	GO:0002237	12	49	1.8	0.00361462	0.043621421
lysosomal membrane	cellular component	GO:0005765	12	49	1.8	0.00361462	0.043621421
animal organ development	biological process	GO:0048513	90	736	0.9	0.00364287	0.043847642
single-organism carbohydrate metabolic process	Undef	GO:0044723	25	147	1.25	0.00373812	0.044876943
metal ion homeostasis	biological process	GO:0055065	19	100	1.39	0.003821	0.045752787
neuron differentiation	biological process	GO:0030182	42	292	1.06	0.00386011	0.046071277
regulation of immune system	biological process	GO:0002682	38	257	1.08	0.00386759	0.046071277
endocytosis	biological process	GO:0006897	20	108	1.36	0.00393342	0.046734754
iron ion transport	biological process	GO:0006826	5	9	4.08	0.00397034	0.047052043
non-membrane-bounded organelle	cellular component	GO:0043228	120	1031	0.85	0.00399719	0.047128003
intracellular non-membrane-bounded organelle	cellular component	GO:0043232	120	1031	0.85	0.00399719	0.047128003
cellular response to cytokine	biological process	GO:0071345	21	116	1.33	0.00400945	0.047151946
single-organism transport	Undef	GO:0044765	77	615	0.92	0.00404633	0.047464626
macromolecule biosynthetic process	biological process	GO:0009059	137	1200	0.84	0.00407052	0.047627102
DNA binding	molecular function	GO:0003677	74	587	0.92	0.00410389	0.047896069
macromolecule localization	biological process	GO:0033036	76	606	0.92	0.0041167	0.047924226
positive regulation of cell	biological process	GO:0008284	28	173	1.19	0.00414794	0.048112288
glycosaminoglycan binding	molecular function	GO:0005539	12	50	1.76	0.00415373	0.048112288
epithelial cell migration	biological process	GO:0010631	13	57	1.67	0.00423219	0.048775997
protein localization to plasma membrane	biological process	GO:0072659	13	57	1.67	0.00423219	0.048775997
cell-cell adhesion via plasma-membrane adhesion molecules	biological process	GO:0098742	10	37	1.98	0.00424436	0.048794223
cellular macromolecule biosynthetic process	biological process	GO:0034645	134	1172	0.84	0.00426956	0.048961916

regulation of multicellular organismal development	biological process	GO:2000026	52	384	0.99	0.00433087	0.04954168
cation homeostasis	biological process	GO:0055080	21	117	1.32	0.00437169	0.049884866

Table S6 The significantly enriched gene ontology (GO) in the comparison of shRIP2+APEC vs. APEC with adjusted p -value ≤ 0.05

Term	Database	ID	Input number	Background number	Rich factor	p value	Corrected p value
intracellular	cellular component	GO:0005622	1097	3785	1.18	2.96E-66	1.59E-62
intracellular part	cellular component	GO:0044424	1063	3636	1.19	1.76E-65	4.71E-62
cell	cellular component	GO:0005623	1189	4280	1.13	5.27E-64	6.92E-61
cell part	cellular component	GO:0044464	1185	4261	1.13	5.77E-64	6.92E-61
cellular process	biological process	GO:0009987	1132	4001	1.15	6.46E-64	6.92E-61
organelle	cellular component	GO:0043226	994	3422	1.18	2.22E-59	1.98E-56
membrane-bounded organelle	cellular component	GO:0043227	916	3072	1.21	2.63E-58	2.01E-55
intracellular organelle	cellular component	GO:0043229	904	3106	1.19	1.50E-53	1.00E-50
intracellular membrane-bounded organelle	cellular component	GO:0043231	809	2742	1.2	3.50E-49	2.08E-46
cytoplasm	cellular component	GO:0005737	782	2632	1.21	2.51E-48	1.35E-45
single-organism process	Undef	GO:0044699	926	3374	1.12	8.92E-46	4.34E-43
single-organism cellular process	Undef	GO:0044763	823	2902	1.16	1.57E-44	7.01E-42
binding	molecular function	GO:0005488	884	3243	1.11	1.92E-42	7.92E-40
metabolic process	biological process	GO:0008152	833	3005	1.13	5.64E-42	2.16E-39
organic substance metabolic process	biological process	GO:0071704	777	2817	1.12	3.11E-38	1.11E-35
primary metabolic process	biological process	GO:0044238	742	2671	1.13	3.31E-37	1.11E-34
cellular metabolic process	biological process	GO:0044237	745	2694	1.13	7.99E-37	2.52E-34
organelle part	cellular component	GO:0044422	586	1970	1.21	1.52E-35	4.53E-33
intracellular organelle part	cellular component	GO:0044446	576	1925	1.22	1.72E-35	4.86E-33
biological regulation	biological process	GO:0065007	760	2799	1.11	2.06E-35	5.53E-33
regulation of biological process	biological process	GO:0050789	714	2619	1.11	1.52E-33	3.88E-31
regulation of cellular process	biological process	GO:0050794	681	2483	1.12	1.59E-32	3.87E-30
macromolecule metabolic process	biological process	GO:0043170	655	2359	1.13	1.76E-32	4.09E-30
nucleus	cellular component	GO:0005634	513	1716	1.22	2.17E-31	4.84E-29
cellular macromolecule metabolic process	biological process	GO:0044260	611	2184	1.14	6.45E-31	1.38E-28
catalytic activity	molecular function	GO:0003824	513	1736	1.2	2.20E-30	4.53E-28
membrane	cellular component	GO:0016020	634	2310	1.12	3.24E-30	6.42E-28
cellular component organization or biogenesis	biological process	GO:0071840	462	1523	1.24	2.33E-29	4.46E-27
cellular component organization	biological process	GO:0016043	444	1462	1.24	2.74E-28	5.07E-26

protein complex	Undef	GO:0043234	372	1159	1.31	2.63E-27	4.69E-25
cytoplasmic part	cellular component	GO:0044444	506	1785	1.15	1.67E-26	2.89E-24
macromolecular complex	cellular component	GO:0032991	418	1391	1.22	7.12E-26	1.19E-23
non-membrane-bounded organelle	cellular component	GO:0043228	330	1031	1.3	4.18E-24	6.59E-22
intracellular non-membrane-bounded organelle	cellular component	GO:0043232	330	1031	1.3	4.18E-24	6.59E-22
protein binding	molecular function	GO:0005515	438	1528	1.17	1.07E-23	1.64E-21
extracellular region part	cellular component	GO:0044421	306	949	1.31	8.46E-23	1.26E-20
extracellular vesicle	cellular component	GO:1903561	239	670	1.45	1.83E-22	2.64E-20
extracellular organelle	cellular component	GO:0043230	239	671	1.45	2.14E-22	2.99E-20
extracellular exosome	cellular component	GO:0070062	238	667	1.45	2.18E-22	2.99E-20
heterocyclic compound binding	molecular function	GO:1901363	457	1652	1.13	3.69E-22	4.94E-20
organic cyclic compound binding	molecular function	GO:0097159	461	1672	1.12	4.07E-22	5.28E-20
response to stimulus	biological process	GO:0050896	500	1859	1.1	4.14E-22	5.28E-20
membrane-bounded vesicle	Undef	GO:0031988	268	822	1.33	1.34E-20	1.66E-18
vesicle	cellular component	GO:0031982	273	847	1.31	2.08E-20	2.53E-18
extracellular region	cellular component	GO:0005576	324	1081	1.22	5.20E-20	6.18E-18
negative regulation of biological process	biological process	GO:0048519	326	1099	1.21	1.43E-19	1.67E-17
organelle organization	biological process	GO:0006996	284	913	1.27	1.98E-19	2.26E-17
nitrogen compound metabolic process	biological process	GO:0006807	447	1683	1.08	6.08E-19	6.78E-17
cellular component biogenesis	biological process	GO:0044085	231	693	1.36	8.04E-19	8.79E-17
protein metabolic process	biological process	GO:0019538	391	1418	1.12	8.26E-19	8.85E-17
cellular nitrogen compound metabolic process	biological process	GO:0034641	424	1580	1.09	1.25E-18	1.31E-16
regulation of primary metabolic process	biological process	GO:0080090	377	1375	1.12	7.84E-18	8.08E-16
cellular response to stimulus	biological process	GO:0051716	414	1553	1.09	8.50E-18	8.59E-16
localization	biological process	GO:0051179	384	1413	1.11	1.22E-17	1.21E-15
negative regulation of cellular process	biological process	GO:0048523	299	1016	1.2	1.25E-17	1.22E-15
organic cyclic compound metabolic process	biological process	GO:1901360	393	1457	1.1	1.33E-17	1.28E-15
heterocycle metabolic process	biological process	GO:0046483	382	1405	1.11	1.41E-17	1.32E-15
cellular component assembly	biological process	GO:0022607	210	625	1.37	1.61E-17	1.49E-15
nucleobase-containing compound metabolic process	biological process	GO:0006139	375	1374	1.11	1.76E-17	1.60E-15
regulation of metabolic process	biological process	GO:0019222	394	1465	1.1	1.79E-17	1.60E-15

small molecule binding	molecular function	GO:0036094	244	773	1.29	1.88E-17	1.65E-15
cellular protein metabolic process	biological process	GO:0044267	354	1276	1.13	2.03E-17	1.75E-15
nuclear part	cellular component	GO:0044428	275	912	1.23	2.15E-17	1.83E-15
cellular aromatic compound metabolic process	biological process	GO:0006725	383	1421	1.1	3.98E-17	3.34E-15
hydrolase activity	molecular function	GO:0016787	228	710	1.31	4.56E-17	3.76E-15
single-organism metabolic	Undef	GO:0044710	296	1017	1.19	6.25E-17	5.07E-15
regulation of cellular metabolic process	biological process	GO:0031323	375	1388	1.1	6.48E-17	5.18E-15
regulation of macromolecule metabolic process	biological process	GO:0060255	374	1385	1.1	7.64E-17	6.02E-15
biosynthetic process	biological process	GO:0009058	400	1511	1.08	8.29E-17	6.43E-15
nucleotide binding	molecular function	GO:0000166	227	720	1.28	2.87E-16	2.17E-14
nucleoside phosphate binding	molecular function	GO:1901265	227	720	1.28	2.87E-16	2.17E-14
macromolecular complex subunit organization	biological process	GO:0043933	190	562	1.38	3.50E-16	2.60E-14
organic substance biosynthetic process	biological process	GO:1901576	390	1482	1.07	4.52E-16	3.32E-14
membrane part	cellular component	GO:0044425	443	1743	1.04	5.05E-16	3.66E-14
positive regulation of biological process	biological process	GO:0048518	325	1179	1.12	9.64E-16	6.89E-14
chromosomal part	cellular component	GO:0044427	105	232	1.84	1.05E-15	7.42E-14
macromolecule modification	biological process	GO:0043412	288	1010	1.16	1.46E-15	1.02E-13
carbohydrate derivative binding	molecular function	GO:0097367	211	663	1.3	1.50E-15	1.03E-13
signaling	biological process	GO:0023052	363	1367	1.08	1.76E-15	1.20E-13
chromosome	cellular component	GO:0005694	109	249	1.78	1.80E-15	1.21E-13
single organism signaling	Undef	GO:0044700	361	1364	1.08	3.17E-15	2.09E-13
purine nucleoside binding	molecular function	GO:0001883	189	574	1.34	3.37E-15	2.20E-13
cellular biosynthetic process	biological process	GO:0044249	378	1449	1.06	3.95E-15	2.55E-13
ribonucleotide binding	molecular function	GO:0032553	191	585	1.33	4.68E-15	2.95E-13
purine nucleotide binding	molecular function	GO:0017076	191	585	1.33	4.68E-15	2.95E-13
nucleoside binding	molecular function	GO:0001882	189	577	1.33	5.07E-15	3.16E-13
purine ribonucleoside binding	molecular function	GO:0032550	188	573	1.34	5.27E-15	3.24E-13
purine ribonucleotide binding	molecular function	GO:0032555	190	582	1.33	5.57E-15	3.39E-13
ribonucleoside binding	molecular function	GO:0032549	188	575	1.33	6.90E-15	4.15E-13
purine ribonucleoside triphosphate binding	molecular function	GO:0035639	187	571	1.33	7.18E-15	4.27E-13
nucleic acid metabolic process	biological process	GO:0090304	328	1215	1.1	7.40E-15	4.35E-13

macromolecular complex assembly	biological process	GO:0065003	144	393	1.49	8.98E-15	5.23E-13
intracellular organelle lumen	cellular component	GO:0070013	257	887	1.18	1.19E-14	6.71E-13
membrane-enclosed lumen	cellular component	GO:0031974	257	887	1.18	1.19E-14	6.71E-13
organelle lumen	cellular component	GO:0043233	257	887	1.18	1.19E-14	6.71E-13
cell communication	biological process	GO:0007154	360	1375	1.07	1.24E-14	6.94E-13
protein modification process	biological process	GO:0036211	273	963	1.15	1.48E-14	8.09E-13
cellular protein modification process	biological process	GO:0006464	273	963	1.15	1.48E-14	8.09E-13
positive regulation of cellular process	biological process	GO:0048522	294	1067	1.12	2.59E-14	1.40E-12
cell periphery	cellular component	GO:0071944	280	1005	1.14	3.75E-14	2.01E-12
phosphorus metabolic process	biological process	GO:0006793	231	783	1.2	5.88E-14	3.12E-12
phosphate-containing compound metabolic process	biological process	GO:0006796	230	782	1.2	8.68E-14	4.56E-12
protein complex assembly	Undef	GO:0006461	119	308	1.57	1.08E-13	5.55E-12
protein complex biogenesis	biological process	GO:0070271	119	308	1.57	1.08E-13	5.55E-12
developmental process	biological process	GO:0032502	356	1383	1.05	1.23E-13	6.24E-12
plasma membrane	cellular component	GO:0005886	271	975	1.13	1.24E-13	6.24E-12
single-organism developmental process	Undef	GO:0044767	353	1369	1.05	1.30E-13	6.51E-12
gene expression	biological process	GO:0010467	337	1296	1.06	1.98E-13	9.81E-12
signal transduction	biological process	GO:0007165	332	1272	1.06	2.03E-13	9.98E-12
nuclear lumen	cellular component	GO:0031981	227	782	1.18	3.81E-13	1.86E-11
response to stress	biological process	GO:0006950	213	723	1.2	6.45E-13	3.11E-11
regulation of response to stimulus	biological process	GO:0048583	223	769	1.18	6.72E-13	3.21E-11
protein complex subunit organization	Undef	GO:0071822	123	336	1.49	8.40E-13	3.98E-11
single-multicellular organism process	Undef	GO:0044707	348	1369	1.04	9.35E-13	4.39E-11
establishment of localization	biological process	GO:0051234	301	1141	1.07	1.03E-12	4.80E-11
microtubule cytoskeleton	cellular component	GO:0015630	100	247	1.65	1.10E-12	5.08E-11
cell cycle	biological process	GO:0007049	126	353	1.45	1.81E-12	8.28E-11
protein dimerization activity	molecular function	GO:0046983	117	318	1.5	2.32E-12	1.05E-10
anatomical structure development	biological process	GO:0048856	328	1285	1.04	2.91E-12	1.31E-10
cellular macromolecular complex assembly	biological process	GO:0034622	102	261	1.59	3.53E-12	1.58E-10
endomembrane system	cellular component	GO:0012505	236	848	1.13	4.53E-12	2.00E-10
regulation of protein metabolic process	biological process	GO:0051246	186	622	1.22	6.76E-12	2.97E-10

macromolecule biosynthetic process	biological process	GO:0009059	308	1200	1.05	8.26E-12	3.60E-10
adenyl nucleotide binding	molecular function	GO:0030554	144	439	1.34	8.38E-12	3.62E-10
adenyl ribonucleotide binding	molecular function	GO:0032559	143	437	1.33	1.14E-11	4.88E-10
ATP binding	molecular function	GO:0005524	141	429	1.34	1.23E-11	5.23E-10
regulation of molecular function	biological process	GO:0065009	181	606	1.22	1.41E-11	5.95E-10
multicellular organismal process	biological process	GO:0032501	367	1500	1	1.44E-11	6.01E-10
regulation of biological quality	biological process	GO:0065008	224	806	1.13	1.79E-11	7.44E-10
cytoskeleton	cellular component	GO:0005856	158	506	1.27	1.87E-11	7.70E-10
cellular macromolecule biosynthetic process	biological process	GO:0034645	299	1172	1.04	2.98E-11	1.22E-09
organelle membrane	cellular component	GO:0031090	158	510	1.26	3.04E-11	1.24E-09
chromosome organization	biological process	GO:0051276	103	278	1.51	3.39E-11	1.37E-09
chromosomal region	cellular component	GO:0098687	53	95	2.27	4.01E-11	1.60E-09
cell cycle process	biological process	GO:0022402	101	273	1.51	5.60E-11	2.22E-09
RNA metabolic process	biological process	GO:0016070	278	1078	1.05	6.00E-11	2.36E-09
ion binding	molecular function	GO:0043167	253	962	1.07	9.26E-11	3.62E-09
transport	biological process	GO:0006810	283	1108	1.04	9.37E-11	3.64E-09
nucleic acid binding	molecular function	GO:0003676	271	1051	1.05	1.07E-10	4.12E-09
regulation of cellular protein metabolic process	biological process	GO:0032268	170	575	1.2	1.13E-10	4.32E-09
cell death	biological process	GO:0008219	131	406	1.31	1.75E-10	6.66E-09
regulation of catalytic activity	biological process	GO:0050790	151	495	1.24	2.06E-10	7.73E-09
intrinsic component of membrane	cellular component	GO:0031224	356	1483	0.98	2.06E-10	7.73E-09
metal ion binding	molecular function	GO:0046872	238	901	1.08	2.42E-10	8.95E-09
integral component of membrane	cellular component	GO:0016021	350	1455	0.98	2.42E-10	8.95E-09
phosphorylation	biological process	GO:0016310	166	564	1.2	2.47E-10	9.05E-09
cation binding	molecular function	GO:0043169	242	921	1.07	2.58E-10	9.39E-09
cellular nitrogen compound biosynthetic process	biological process	GO:0044271	292	1166	1.02	2.69E-10	9.75E-09
protein heterodimerization activity	molecular function	GO:0046982	64	141	1.85	3.46E-10	1.25E-08
cellular developmental process	biological process	GO:0048869	249	959	1.06	3.64E-10	1.30E-08
multicellular organism development	biological process	GO:0007275	285	1136	1.02	3.82E-10	1.36E-08
programmed cell death	biological process	GO:0012501	124	383	1.32	4.50E-10	1.59E-08

intracellular signal transduction	biological process	GO:0035556	175	613	1.16	5.66E-10	1.98E-08
cell proliferation	biological process	GO:0008283	131	416	1.28	6.29E-10	2.19E-08
apoptotic process	biological process	GO:0006915	122	377	1.32	6.35E-10	2.19E-08
pyrophosphatase activity	molecular function	GO:0016462	88	236	1.52	6.89E-10	2.37E-08
mitotic cell cycle	biological process	GO:0000278	77	193	1.63	7.11E-10	2.43E-08
system development	biological process	GO:0048731	257	1007	1.04	7.53E-10	2.55E-08
immune system process	biological process	GO:0002376	144	475	1.24	7.59E-10	2.56E-08
nucleoside-triphosphatase activity	molecular function	GO:0017111	84	221	1.55	8.03E-10	2.69E-08
transferase activity	molecular function	GO:0016740	190	687	1.13	8.62E-10	2.87E-08
regulation of signaling	biological process	GO:0023051	185	665	1.13	9.87E-10	3.26E-08
hydrolase activity, acting on acid anhydrides, in phosphorus-containing anhydrides	molecular function	GO:0016818	88	239	1.5	1.14E-09	3.71E-08
hydrolase activity, acting on acid anhydrides	molecular function	GO:0016817	88	239	1.5	1.14E-09	3.71E-08
DNA packaging complex	cellular component	GO:0044815	34	48	2.89	1.51E-09	4.91E-08
regulation of signal transduction	biological process	GO:0009966	169	598	1.15	2.05E-09	6.62E-08
regulation of nitrogen compound metabolic process	biological process	GO:0051171	247	971	1.04	2.09E-09	6.70E-08
nucleobase-containing compound biosynthetic process	biological process	GO:0034654	243	953	1.04	2.37E-09	7.55E-08
mitotic cell cycle process	biological process	GO:1903047	70	173	1.65	2.60E-09	8.24E-08
organic cyclic compound biosynthetic process	biological process	GO:1901362	252	999	1.03	2.72E-09	8.58E-08
chromosome, centromeric region	cellular component	GO:0000775	36	56	2.62	3.34E-09	1.04E-07
negative regulation of macromolecule metabolic	biological process	GO:0010605	157	548	1.17	3.55E-09	1.10E-07
heterocycle biosynthetic process	biological process	GO:0018130	246	973	1.03	3.57E-09	1.11E-07
cytoskeletal protein binding	molecular function	GO:0008092	80	214	1.52	3.67E-09	1.13E-07
regulation of cell communication	biological process	GO:0010646	180	657	1.12	4.29E-09	1.31E-07
cell differentiation	biological process	GO:0030154	224	868	1.05	4.38E-09	1.33E-07
macromolecule localization	biological process	GO:0033036	169	606	1.14	4.52E-09	1.37E-07
cellular localization	biological process	GO:0051641	153	534	1.17	5.58E-09	1.68E-07
nucleosome	cellular component	GO:0000786	32	46	2.83	6.22E-09	1.86E-07
aromatic compound biosynthetic process	biological process	GO:0019438	245	976	1.02	6.53E-09	1.94E-07
cytoskeletal part	cellular component	GO:0044430	122	397	1.25	7.77E-09	2.30E-07
negative regulation of metabolic process	biological process	GO:0009892	165	594	1.13	8.62E-09	2.54E-07

regulation of cell proliferation	biological process	GO:0042127	107	333	1.31	9.53E-09	2.79E-07
regulation of cellular component organization	biological process	GO:0051128	149	523	1.16	1.19E-08	3.47E-07
whole membrane	cellular component	GO:0098805	93	275	1.38	1.21E-08	3.49E-07
regulation of cell death	biological process	GO:0010941	107	335	1.3	1.23E-08	3.55E-07
regulation of nucleobase-containing compound metabolic process	biological process	GO:0019219	230	913	1.03	1.50E-08	4.29E-07
regulation of biosynthetic process	biological process	GO:0009889	240	963	1.02	1.57E-08	4.45E-07
protein-DNA complex	cellular component	GO:0032993	38	67	2.31	1.57E-08	4.45E-07
negative regulation of cellular metabolic process	biological process	GO:0031324	154	549	1.14	1.59E-08	4.47E-07
DNA conformation change	biological process	GO:0071103	39	71	2.24	1.97E-08	5.54E-07
chromatin	cellular component	GO:0000785	58	138	1.71	2.01E-08	5.62E-07
regulation of hydrolase activity	biological process	GO:0051336	89	262	1.38	2.08E-08	5.77E-07
protein localization	biological process	GO:0008104	147	520	1.15	2.24E-08	6.16E-07
bounding membrane of organelle	cellular component	GO:0098588	105	331	1.29	2.24E-08	6.16E-07
regulation of cellular biosynthetic process	biological process	GO:0031326	237	954	1.01	2.41E-08	6.60E-07
identical protein binding	molecular function	GO:0042802	94	285	1.34	2.69E-08	7.32E-07
regulation of macromolecule biosynthetic process	biological process	GO:0010556	228	911	1.02	2.71E-08	7.32E-07
catabolic process	biological process	GO:0009056	125	422	1.21	2.94E-08	7.91E-07
hydrolase activity, acting on ester bonds	molecular function	GO:0016788	77	216	1.45	3.63E-08	9.73E-07
regulation of gene expression	biological process	GO:0010468	240	976	1	4.03E-08	1.07E-06
regulation of programmed cell death	biological process	GO:0043067	102	323	1.29	4.25E-08	1.13E-06
regulation of cellular macromolecule biosynthetic process	biological process	GO:2000112	222	888	1.02	4.44E-08	1.17E-06
organic substance catabolic process	biological process	GO:1901575	123	418	1.2	5.12E-08	1.34E-06
positive regulation of response to stimulus	biological process	GO:0048584	117	391	1.22	5.15E-08	1.34E-06
positive regulation of metabolic process	biological process	GO:0009893	183	700	1.07	5.42E-08	1.41E-06
immune response	biological process	GO:0006955	79	227	1.42	5.55E-08	1.44E-06
transcription, DNA-templated	biological process	GO:0006351	209	827	1.03	5.60E-08	1.44E-06
protein phosphorylation	biological process	GO:0006468	133	465	1.17	5.91E-08	1.52E-06
regulation of apoptotic process	biological process	GO:0042981	100	317	1.29	5.99E-08	1.53E-06
regulation of immune system process	biological process	GO:0002682	86	257	1.36	6.14E-08	1.56E-06

DNA binding	molecular function	GO:0003677	159	587	1.1	6.29E-08	1.59E-06
vacuole	cellular component	GO:0005773	85	253	1.37	6.33E-08	1.59E-06
single-organism organelle organization	Undef	GO:1902589	130	452	1.17	6.40E-08	1.60E-06
small molecule metabolic process	biological process	GO:0044281	130	452	1.17	6.40E-08	1.60E-06
regulation of multicellular organismal process	biological process	GO:0051239	157	578	1.11	6.56E-08	1.63E-06
RNA biosynthetic process	biological process	GO:0032774	211	839	1.02	6.60E-08	1.63E-06
nucleoplasm	cellular component	GO:0005654	154	564	1.11	6.65E-08	1.63E-06
mitochondrion	cellular component	GO:0005739	130	453	1.17	7.10E-08	1.74E-06
protein-DNA complex assembly	biological process	GO:0065004	36	66	2.22	7.79E-08	1.89E-06
DNA metabolic process	biological process	GO:0006259	77	221	1.42	7.81E-08	1.89E-06
nucleic acid-templated transcription	biological process	GO:0097659	210	837	1.02	8.23E-08	1.99E-06
regulation of developmental process	biological process	GO:0050793	143	517	1.13	1.03E-07	2.48E-06
protein-DNA complex subunit organization	biological process	GO:0071824	37	71	2.12	1.25E-07	2.99E-06
nuclear chromosome part	cellular component	GO:0044454	55	136	1.65	1.27E-07	3.02E-06
nuclear chromosome	cellular component	GO:0000228	56	140	1.63	1.31E-07	3.10E-06
regulation of localization	biological process	GO:0032879	143	520	1.12	1.37E-07	3.23E-06
cellular catabolic process	biological process	GO:0044248	106	352	1.23	1.66E-07	3.89E-06
macromolecule catabolic process	biological process	GO:0009057	90	282	1.3	1.76E-07	4.12E-06
regulation of protein modification process	biological process	GO:0031399	116	400	1.18	2.26E-07	5.27E-06
cellular response to stress	biological process	GO:0033554	114	391	1.19	2.29E-07	5.31E-06
cellular macromolecule catabolic process	biological process	GO:0044265	79	237	1.36	2.35E-07	5.43E-06
positive regulation of macromolecule metabolic	biological process	GO:0010604	171	660	1.06	2.40E-07	5.51E-06
single-organism localization	Undef	GO:1902578	173	671	1.05	2.66E-07	6.10E-06
proteolysis	biological process	GO:0006508	117	407	1.17	2.92E-07	6.65E-06
positive regulation of cellular metabolic process	biological process	GO:0031325	167	645	1.05	3.43E-07	7.78E-06
positive regulation of molecular function	biological process	GO:0044093	103	346	1.21	3.86E-07	8.69E-06
regulation of cell cycle	biological process	GO:0051726	72	211	1.39	3.86E-07	8.69E-06
organelle fission	biological process	GO:0048285	51	127	1.64	4.23E-07	9.48E-06
animal organ development	biological process	GO:0048513	185	736	1.02	4.36E-07	9.74E-06
organic substance transport	biological process	GO:0071702	151	571	1.08	4.44E-07	9.86E-06
response to chemical	biological process	GO:0042221	189	756	1.02	4.46E-07	9.87E-06

negative regulation of protein metabolic process	biological process	GO:0051248	80	246	1.32	4.48E-07	9.88E-06
single-organism biosynthetic process	Undef	GO:0044711	98	326	1.22	5.08E-07	1.11E-05
establishment of localization in cell	biological process	GO:0051649	115	406	1.15	6.76E-07	1.48E-05
envelope	cellular component	GO:0031975	83	263	1.29	7.54E-07	1.64E-05
cytosol	cellular component	GO:0005829	124	449	1.13	7.56E-07	1.64E-05
positive regulation of catalytic activity	biological process	GO:0043085	88	285	1.26	7.60E-07	1.64E-05
positive regulation of immune system process	biological process	GO:0002684	57	154	1.51	7.79E-07	1.67E-05
movement of cell or subcellular component	biological process	GO:0006928	111	389	1.16	7.82E-07	1.67E-05
chromatin organization	biological process	GO:0006325	65	187	1.42	8.26E-07	1.76E-05
regulation of response to stress	biological process	GO:0080134	79	247	1.3	9.00E-07	1.91E-05
cellular protein complex	Undef	GO:0043623	52	135	1.57	9.00E-07	1.91E-05
nuclear division	biological process	GO:0000280	48	120	1.63	9.95E-07	2.10E-05
organelle envelope	cellular component	GO:0031967	82	261	1.28	1.01E-06	2.13E-05
catalytic complex	cellular component	GO:1902494	98	333	1.2	1.11E-06	2.32E-05
negative regulation of cellular protein metabolic process	biological process	GO:0032269	74	228	1.32	1.26E-06	2.62E-05
membrane organization	biological process	GO:0061024	69	207	1.36	1.32E-06	2.74E-05
regulation of phosphorus metabolic process	biological process	GO:0051174	112	399	1.14	1.34E-06	2.77E-05
regulation of phosphate metabolic process	biological process	GO:0019220	112	399	1.14	1.34E-06	2.77E-05
positive regulation of hydrolase activity	biological process	GO:0051345	57	158	1.47	1.50E-06	3.07E-05
oxidation-reduction process	biological process	GO:0055114	86	282	1.24	1.53E-06	3.12E-05
regulation of cell differentiation	biological process	GO:0045595	103	359	1.17	1.57E-06	3.19E-05
endosome	cellular component	GO:0005768	65	192	1.38	1.72E-06	3.49E-05
lysosome	cellular component	GO:0005764	40	93	1.75	1.89E-06	3.81E-05
lytic vacuole	cellular component	GO:0000323	40	93	1.75	1.89E-06	3.81E-05
cell adhesion	biological process	GO:0007155	94	320	1.2	1.94E-06	3.88E-05
biological adhesion	biological process	GO:0022610	94	320	1.2	1.94E-06	3.88E-05
cell motility	biological process	GO:0048870	90	303	1.21	2.18E-06	4.35E-05
negative regulation of response to stimulus	biological process	GO:0048585	93	317	1.2	2.29E-06	4.55E-05
cell division	biological process	GO:0051301	45	113	1.62	2.34E-06	4.63E-05
kinetochore	cellular component	GO:0000776	23	36	2.6	2.38E-06	4.68E-05
localization of cell	biological process	GO:0051674	90	304	1.21	2.44E-06	4.79E-05

ATPase activity	molecular function	GO:0016887	49	129	1.55	2.51E-06	4.89E-05
protein catabolic process	biological process	GO:0030163	70	216	1.32	2.51E-06	4.89E-05
mitotic nuclear division	Undef	GO:0007067	40	95	1.72	2.86E-06	5.56E-05
cellular response to DNA damage stimulus	biological process	GO:0006974	61	179	1.39	2.97E-06	5.74E-05
actin binding	molecular function	GO:0003779	41	99	1.69	3.00E-06	5.78E-05
response to organic substance	biological process	GO:0010033	144	561	1.05	3.18E-06	6.11E-05
cellular response to chemical stimulus	biological process	GO:0070887	138	532	1.06	3.21E-06	6.14E-05
inflammatory response	biological process	GO:0006954	43	107	1.64	3.23E-06	6.16E-05
anatomical structure morphogenesis	biological process	GO:0009653	164	660	1.01	3.40E-06	6.46E-05
DNA repair	biological process	GO:0006281	46	119	1.57	3.45E-06	6.53E-05
regulation of transcription, DNA-templated	biological process	GO:0006355	191	796	0.98	3.68E-06	6.95E-05
cytoskeleton organization	biological process	GO:0007010	83	277	1.22	4.03E-06	7.58E-05
regulation of RNA metabolic process	biological process	GO:0051252	199	838	0.97	4.08E-06	7.64E-05
regulation of cellular component biogenesis	biological process	GO:0044087	66	203	1.32	4.38E-06	8.17E-05
regulation of RNA biosynthetic process	biological process	GO:2001141	193	809	0.97	4.46E-06	8.29E-05
cell migration	biological process	GO:0016477	84	283	1.21	4.78E-06	8.85E-05
transferase activity, transferring phosphorus-containing groups	molecular function	GO:0016772	83	279	1.21	5.05E-06	9.33E-05
regulation of phosphorylation	biological process	GO:0042325	97	343	1.15	5.13E-06	9.44E-05
DNA packaging	biological process	GO:0006323	26	48	2.21	5.26E-06	9.65E-05
positive regulation of apoptotic process	biological process	GO:0043065	44	114	1.57	5.74E-06	0.000104948
regulation of nucleic acid-templated transcription	biological process	GO:1903506	192	808	0.97	5.80E-06	0.000105724
microtubule	cellular component	GO:0005874	35	80	1.78	6.03E-06	0.000109462
establishment of protein localization	biological process	GO:0045184	111	411	1.1	6.52E-06	0.000117923
single-organism transport	Undef	GO:0044765	153	615	1.01	6.78E-06	0.000122248
positive regulation of programmed cell death	biological process	GO:0043068	44	115	1.56	6.86E-06	0.000123241
regulation of multicellular organismal development	biological process	GO:2000026	105	384	1.11	7.35E-06	0.000131615
spindle	cellular component	GO:0005819	28	56	2.04	7.45E-06	0.000132385
negative regulation of signaling	biological process	GO:0023057	82	278	1.2	7.46E-06	0.000132385
negative regulation of cell communication	biological process	GO:0010648	82	278	1.2	7.46E-06	0.000132385
microtubule organizing center	cellular component	GO:0005815	52	148	1.43	7.71E-06	0.000136337
positive regulation of cell death	biological process	GO:0010942	44	116	1.55	8.17E-06	0.000143947

cellular protein catabolic process	biological process	GO:0044257	60	182	1.34	8.22E-06	0.000144456
nucleolus	cellular component	GO:0005730	66	208	1.29	8.40E-06	0.000147122
vesicle-mediated transport	biological process	GO:0016192	81	275	1.2	8.82E-06	0.000153956
microtubule associated complex	cellular component	GO:0005875	21	34	2.52	9.49E-06	0.000165003
phosphoric ester hydrolase activity	molecular function	GO:0042578	42	109	1.57	9.55E-06	0.000165605
lipid metabolic process	biological process	GO:0006629	83	285	1.19	9.76E-06	0.00016868
regulation of protein phosphorylation	biological process	GO:0001932	91	322	1.15	1.01E-05	0.000173766
cellular macromolecule localization	biological process	GO:0070727	100	364	1.12	1.02E-05	0.000174821
regulation of cellular component movement	biological process	GO:0051270	57	171	1.36	1.05E-05	0.000179359
cellular protein localization	biological process	GO:0034613	99	360	1.12	1.09E-05	0.000185385
molecular function regulator	molecular function	GO:0098772	81	277	1.19	1.10E-05	0.000186659
protein complex binding	Undef	GO:0032403	59	180	1.34	1.12E-05	0.000189991
positive regulation of protein metabolic process	biological process	GO:0051247	95	342	1.13	1.16E-05	0.000196362
regulation of intracellular signal transduction	biological process	GO:1902531	99	361	1.12	1.19E-05	0.000201155
locomotion	biological process	GO:0040011	96	347	1.13	1.20E-05	0.000201483
small GTPase mediated signal transduction	biological process	GO:0007264	52	151	1.4	1.21E-05	0.000202462
chromosome segregation	biological process	GO:0007059	30	65	1.88	1.21E-05	0.000202462
regulation of response to external stimulus	biological process	GO:0032101	55	164	1.37	1.29E-05	0.000214168
defense response	biological process	GO:0006952	77	261	1.2	1.38E-05	0.000229374
negative regulation of signal transduction	biological process	GO:0009968	76	257	1.2	1.46E-05	0.00024208
vacuolar part	cellular component	GO:0044437	47	132	1.45	1.56E-05	0.000257245
vacuolar membrane	cellular component	GO:0005774	45	124	1.48	1.60E-05	0.000262593
protein homodimerization activity	molecular function	GO:0042803	58	179	1.32	1.74E-05	0.00028559
protein dephosphorylation	biological process	GO:0006470	32	74	1.76	1.78E-05	0.000290505
regulation of protein complex assembly	biological process	GO:0043254	38	97	1.6	1.85E-05	0.000300829
protein transport	biological process	GO:0015031	99	366	1.1	1.90E-05	0.000307902
homeostatic process	biological process	GO:0042592	96	352	1.11	1.92E-05	0.000311315
enzyme binding	molecular function	GO:0019899	97	357	1.11	1.98E-05	0.000318758
negative regulation of biosynthetic process	biological process	GO:0009890	94	343	1.12	2.00E-05	0.00032131
regulation of organelle organization	biological process	GO:0033043	75	256	1.19	2.16E-05	0.000345979
positive regulation of nitrogen compound metabolic process	biological process	GO:0051173	102	382	1.09	2.22E-05	0.000355457

chromatin assembly or disassembly	biological process	GO:0006333	25	50	2.04	2.25E-05	0.000358988
nucleosome assembly	biological process	GO:0006334	22	40	2.24	2.28E-05	0.000363205
signal transduction by protein phosphorylation	biological process	GO:0023014	60	190	1.29	2.36E-05	0.000373858
single-organism membrane organization	Undef	GO:0044802	57	177	1.31	2.37E-05	0.000373858
microtubule-based process	biological process	GO:0007017	51	152	1.37	2.58E-05	0.000404762
regulation of cell migration	biological process	GO:0030334	51	152	1.37	2.58E-05	0.000404762
mitochondrial envelope	cellular component	GO:0005740	54	165	1.33	2.67E-05	0.000417491
negative regulation of cellular biosynthetic process	biological process	GO:0031327	93	342	1.11	2.82E-05	0.000440509
response to oxygen-containing compound	biological process	GO:1901700	70	236	1.21	2.87E-05	0.000447541
organonitrogen compound metabolic process	biological process	GO:1901564	133	536	1.01	2.91E-05	0.000452187
proteolysis involved in cellular protein catabolic process	biological process	GO:0051603	55	170	1.32	2.93E-05	0.00045379
endoplasmic reticulum	cellular component	GO:0005783	90	329	1.11	3.13E-05	0.000480738
positive regulation of GTPase activity	biological process	GO:0043547	37	96	1.57	3.13E-05	0.000480738
ATPase activity, coupled	molecular function	GO:0042623	37	96	1.57	3.13E-05	0.000480738
regulation of immune response	biological process	GO:0050776	41	112	1.49	3.17E-05	0.00048456
kinase activity	molecular function	GO:0016301	70	237	1.2	3.21E-05	0.000490323
cell junction	cellular component	GO:0030054	80	283	1.15	3.32E-05	0.000505304
negative regulation of macromolecule biosynthetic process	biological process	GO:0010558	89	325	1.12	3.34E-05	0.000507202
phosphatase activity	molecular function	GO:0016791	34	85	1.63	3.55E-05	0.000537032
nucleosome organization	biological process	GO:0034728	23	45	2.08	3.63E-05	0.000548259
intracellular transport	biological process	GO:0046907	89	327	1.11	4.03E-05	0.000606069
centrosome	cellular component	GO:0005813	44	126	1.42	4.17E-05	0.000625552
negative regulation of cellular macromolecule biosynthetic process	biological process	GO:2000113	87	318	1.11	4.18E-05	0.000626211
positive regulation of immune response	biological process	GO:0050778	34	86	1.61	4.28E-05	0.000639095
dephosphorylation	biological process	GO:0016311	42	118	1.45	4.32E-05	0.000643285
cellular response to organic substance	biological process	GO:0071310	115	453	1.03	4.41E-05	0.000654882
regulation of cell motility	biological process	GO:2000145	51	156	1.33	4.49E-05	0.000663489
single-organism cellular localization	Undef	GO:1902580	68	231	1.2	4.50E-05	0.000663489
carbohydrate derivative metabolic process	biological process	GO:1901135	80	287	1.14	4.94E-05	0.00072737

growth	biological process	GO:0040007	68	232	1.19	5.02E-05	0.00073645
phosphoprotein phosphatase activity	molecular function	GO:0004721	26	57	1.86	5.25E-05	0.000768607
chromatin assembly	biological process	GO:0031497	22	43	2.08	5.27E-05	0.000768607
macromolecular complex binding	molecular function	GO:0044877	83	302	1.12	5.44E-05	0.000791333
anchoring junction	cellular component	GO:0070161	45	132	1.39	5.48E-05	0.000796141
cell surface receptor signaling pathway	biological process	GO:0007166	143	596	0.98	5.89E-05	0.000852698
establishment of organelle localization	biological process	GO:0051656	33	84	1.6	6.08E-05	0.000877538
negative regulation of nitrogen compound metabolic process	biological process	GO:0051172	92	346	1.08	6.20E-05	0.00089215
oxidoreductase activity	molecular function	GO:0016491	68	235	1.18	6.94E-05	0.000996169
regulation of GTPase activity	biological process	GO:0043087	40	113	1.44	7.15E-05	0.00102393
negative regulation of gene expression	biological process	GO:0010629	93	353	1.07	7.51E-05	0.001072877
regulation of locomotion	biological process	GO:0040012	52	165	1.28	8.28E-05	0.001180355
mitochondrial membrane	cellular component	GO:0031966	49	152	1.31	8.31E-05	0.00118041
modification-dependent macromolecule catabolic process	biological process	GO:0043632	48	148	1.32	8.65E-05	0.001226511
adherens junction	cellular component	GO:0005912	44	131	1.37	8.72E-05	0.001232098
enzyme regulator activity	molecular function	GO:0030234	64	219	1.19	8.80E-05	0.00124027
organelle assembly	biological process	GO:0070925	53	170	1.27	8.96E-05	0.001259449
positive regulation of cellular protein metabolic process	biological process	GO:0032270	85	317	1.09	9.08E-05	0.001273321
actin cytoskeleton	cellular component	GO:0015629	41	119	1.4	9.50E-05	0.001328656
positive regulation of nucleobase-containing compound metabolic process	biological process	GO:0045935	95	366	1.06	9.98E-05	0.001392648
regulation of mitotic cell cycle	biological process	GO:0007346	36	99	1.48	0.000103724	0.001443247
regulation of endopeptidase activity	biological process	GO:0052548	33	87	1.55	0.000104279	0.001447208
phosphotransferase activity, alcohol group as acceptor	molecular function	GO:0016773	61	207	1.2	0.000104792	0.001450568
regulation of defense response	biological process	GO:0031347	41	120	1.39	0.000110178	0.001521188
MAPK cascade	biological process	GO:0000165	53	172	1.26	0.00011452	0.001577086
organophosphate metabolic process	biological process	GO:0019637	68	240	1.15	0.000116785	0.001604145
GTP binding	molecular function	GO:0005525	47	146	1.31	0.000117676	0.001612247
membrane protein complex	cellular component	GO:0098796	82	306	1.09	0.000121461	0.001659866
organelle localization	biological process	GO:0051640	36	100	1.47	0.000122012	0.001663149
organic cyclic compound catabolic process	biological process	GO:1901361	32	84	1.55	0.000123498	0.001676911

lipid biosynthetic process	biological process	GO:0008610	42	125	1.37	0.000123648	0.001676911
nuclear chromatin	cellular component	GO:0000790	33	88	1.53	0.000124063	0.001678292
positive regulation of protein complex assembly	biological process	GO:0031334	23	50	1.87	0.000125943	0.001699432
guanyl ribonucleotide binding	molecular function	GO:0032561	48	151	1.3	0.0001284	0.001728234
cellular lipid metabolic process	biological process	GO:0044255	61	209	1.19	0.000130532	0.001752528
mitochondrial part	cellular component	GO:0044429	62	214	1.18	0.000136993	0.00183011
negative regulation of cell death	biological process	GO:0060548	62	214	1.18	0.000136993	0.00183011
negative regulation of protein modification process	biological process	GO:0031400	43	130	1.35	0.00013762	0.001833905
organonitrogen compound biosynthetic process	biological process	GO:1901566	95	370	1.05	0.000138368	0.001839299
response to external stimulus	biological process	GO:0009605	106	424	1.02	0.000140492	0.001862081
protein modification by small protein conjugation or removal	biological process	GO:0070647	65	228	1.16	0.000140777	0.001862081
extracellular space	cellular component	GO:0005615	82	308	1.08	0.000145104	0.001914582
regulation of protein kinase activity	biological process	GO:0045859	53	174	1.24	0.000145633	0.001916759
guanyl nucleotide binding	molecular function	GO:0019001	48	152	1.29	0.000145984	0.001916759
regulation of kinase activity	biological process	GO:0043549	56	188	1.21	0.000155945	0.002042537
negative regulation of multicellular organismal process	biological process	GO:0051241	66	234	1.15	0.000162233	0.002119715
positive regulation of cellular component organization	biological process	GO:0051130	69	248	1.13	0.000163451	0.002130429
protein oligomerization	biological process	GO:0051259	35	98	1.46	0.000170345	0.002214892
regulation of transferase activity	biological process	GO:0051338	60	207	1.18	0.000171963	0.00223052
regulation of peptidase activity	biological process	GO:0052547	33	90	1.49	0.000174028	0.00225185
transition metal ion binding	molecular function	GO:0046914	96	378	1.03	0.000177417	0.00229018
regulation of proteolysis	biological process	GO:0030162	46	145	1.29	0.000181851	0.002341768
small molecule biosynthetic process	biological process	GO:0044283	41	124	1.35	0.000195362	0.002509726
positive regulation of cell proliferation	biological process	GO:0008284	52	173	1.22	0.000218588	0.002801372
negative regulation of programmed cell death	biological process	GO:0043069	59	205	1.17	0.000226097	0.002890697
cell cycle phase transition	biological process	GO:0044770	34	96	1.44	0.000237546	0.003022651
endosomal part	cellular component	GO:0044440	34	96	1.44	0.000237546	0.003022651
vacuolar transport	biological process	GO:0007034	32	88	1.48	0.000244088	0.003098526
positive regulation of biosynthetic process	biological process	GO:0009891	98	392	1.02	0.000244749	0.00309958
aromatic compound catabolic process	biological process	GO:0019439	30	80	1.53	0.000246152	0.00310396

regulation of inflammatory response	biological process	GO:0050727	23	53	1.77	0.000246254	0.00310396
protein tetramerization	biological process	GO:0051262	17	32	2.16	0.000259273	0.003260386
negative regulation of nucleobase-containing compound metabolic process	biological process	GO:0045934	82	316	1.06	0.000287869	0.003611511
RNA binding	molecular function	GO:0003723	108	444	0.99	0.000291201	0.003644775
multi-organism process	biological process	GO:0051704	84	326	1.05	0.000295639	0.003691694
regulation of cellular component size	biological process	GO:0032535	36	106	1.38	0.000306659	0.003820405
transcription from RNA polymerase II promoter	biological process	GO:0006366	114	475	0.98	0.000310281	0.003856551
negative regulation of transcription, DNA-templated	biological process	GO:0045892	72	269	1.09	0.000311673	0.003864892
negative regulation of RNA biosynthetic process	biological process	GO:1902679	73	274	1.09	0.000318473	0.003934236
cell-substrate adherens junction	cellular component	GO:0005924	39	119	1.34	0.000318734	0.003934236
cellular response to oxygen-containing compound	biological process	GO:1901701	47	154	1.24	0.00032217	0.003967504
negative regulation of cell cycle	biological process	GO:0045786	34	98	1.41	0.000323587	0.00397582
positive regulation of defense response	biological process	GO:0031349	21	47	1.82	0.000331535	0.004064149
tissue development	biological process	GO:0009888	105	431	0.99	0.000334299	0.004088679
heterocycle catabolic process	biological process	GO:0046700	29	78	1.51	0.000347095	0.004225878
cellular nitrogen compound catabolic process	biological process	GO:0044270	29	78	1.51	0.000347095	0.004225878
cell-cell adhesion	biological process	GO:0098609	57	200	1.16	0.000350325	0.004245905
negative regulation of apoptotic process	biological process	GO:0043066	57	200	1.16	0.000350325	0.004245905
transporter activity	molecular function	GO:0005215	97	392	1.01	0.000353028	0.004269011
cell-substrate junction	cellular component	GO:0030055	39	120	1.32	0.000365279	0.004407203
immune system development	biological process	GO:0002520	61	219	1.13	0.000367946	0.004429404
modification-dependent protein catabolic process	biological process	GO:0019941	44	142	1.26	0.000376099	0.004517401
regulation of transcription from RNA polymerase II promoter	biological process	GO:0006357	110	458	0.98	0.000382329	0.004581957
nucleobase-containing small molecule metabolic process	biological process	GO:0055086	50	169	1.21	0.000386445	0.004620954
endosome membrane	cellular component	GO:0010008	31	87	1.45	0.0004016	0.004791476
negative regulation of nucleic acid-templated transcription	biological process	GO:1903507	72	272	1.08	0.000406583	0.004829535
negative regulation of immune system process	biological process	GO:0002683	30	83	1.47	0.000407229	0.004829535
DNA replication	biological process	GO:0006260	26	67	1.58	0.000407495	0.004829535
GTPase activity	molecular function	GO:0003924	27	71	1.55	0.000411454	0.004865697
negative regulation of molecular function	biological process	GO:0044092	62	225	1.12	0.000418927	0.004943154

focal adhesion	cellular component	GO:0005925	38	117	1.32	0.00043556	0.005128121
DNA geometric change	biological process	GO:0032392	14	24	2.38	0.000442365	0.005185449
condensed chromosome, centromeric region	cellular component	GO:0000779	14	24	2.38	0.000442365	0.005185449
positive regulation of signaling	biological process	GO:0023056	86	341	1.03	0.000448557	0.005246552
positive regulation of cellular component biogenesis	biological process	GO:0044089	37	113	1.33	0.0004533	0.005290481
mitotic cell cycle phase transition	biological process	GO:0044772	31	88	1.44	0.000470221	0.005476033
protein kinase activity	molecular function	GO:0004672	52	180	1.18	0.000476972	0.005542603
cell activation	biological process	GO:0001775	55	194	1.16	0.000488337	0.005662379
tubulin binding	molecular function	GO:0015631	27	72	1.53	0.000490198	0.005671689
positive regulation of signal transduction	biological process	GO:0009967	79	308	1.05	0.000491636	0.005676068
positive regulation of cellular biosynthetic process	biological process	GO:0031328	94	382	1	0.000503066	0.005795532
single organism cell adhesion	Undef	GO:0098602	51	176	1.18	0.000506883	0.005826979
positive regulation of cell communication	biological process	GO:0010647	85	338	1.02	0.0005194	0.005958081
mRNA metabolic process	biological process	GO:0016071	42	136	1.26	0.000532592	0.006096362
negative regulation of RNA metabolic process	biological process	GO:0051253	73	280	1.06	0.000535284	0.006114109
double-strand break repair	biological process	GO:0006302	22	53	1.69	0.000538262	0.006135044
ubiquitin-dependent protein catabolic process	biological process	GO:0006511	43	141	1.24	0.000570796	0.006492052
endosomal transport	biological process	GO:0016197	28	77	1.48	0.000577686	0.006556492
cell growth	biological process	GO:0016049	37	115	1.31	0.000594108	0.006728617
microtubule binding	molecular function	GO:0008017	20	46	1.77	0.000597931	0.006757627
spindle pole	cellular component	GO:0000922	14	25	2.28	0.000600638	0.006773928
regulation of MAPK cascade	biological process	GO:0043408	46	155	1.21	0.000610191	0.006867212
nuclear chromosome segregation	biological process	GO:0098813	21	50	1.71	0.000631641	0.007093713
endomembrane system organization	biological process	GO:0010256	41	133	1.26	0.000633923	0.007104452
negative regulation of cellular component organization	biological process	GO:0051129	43	142	1.23	0.000643073	0.007191947
protein ubiquitination	biological process	GO:0016567	52	183	1.16	0.000654228	0.007301462
regulation of protein polymerization	biological process	GO:0032271	22	54	1.66	0.000657229	0.007319695
cell development	biological process	GO:0048468	114	487	0.95	0.000673999	0.007490901
regulation of cytoskeleton organization	biological process	GO:0051493	37	116	1.3	0.000678046	0.007513867
lipid binding	molecular function	GO:0008289	42	138	1.24	0.000678871	0.007513867
regulation of cell development	biological process	GO:0060284	53	188	1.15	0.000680367	0.007514899

protein polymerization	biological process	GO:0051258	25	66	1.54	0.000692191	0.007629765
kinase binding	molecular function	GO:0019900	36	112	1.31	0.000708738	0.007796114
nucleotide metabolic process	biological process	GO:0009117	45	152	1.21	0.000724133	0.007949142
endocytosis	biological process	GO:0006897	35	108	1.32	0.000739589	0.008089882
histone binding	molecular function	GO:0042393	20	47	1.73	0.000739974	0.008089882
apoptotic signaling pathway	biological process	GO:0097190	40	130	1.25	0.000754652	0.00823355
cell surface	cellular component	GO:0009986	46	157	1.19	0.00076366	0.008303974
positive regulation of macromolecule biosynthetic process	biological process	GO:0010557	88	358	1	0.000764207	0.008303974
intracellular protein transport	biological process	GO:0006886	60	222	1.1	0.000767153	0.008319108
late endosome	cellular component	GO:0005770	22	55	1.63	0.000798335	0.008639764
negative regulation of gene expression, epigenetic	biological process	GO:0045814	14	26	2.19	0.0008044	0.008687843
nervous system development	biological process	GO:0007399	114	490	0.95	0.00081122	0.008743875
regulation of cell cycle phase transition	biological process	GO:1901987	24	63	1.55	0.000822427	0.008846875
calcium ion binding	molecular function	GO:0005509	54	195	1.13	0.000860308	0.009235808
circulatory system development	biological process	GO:0072359	66	252	1.07	0.00086781	0.009279158
cardiovascular system development	biological process	GO:0072358	66	252	1.07	0.00086781	0.009279158
RNA processing	biological process	GO:0006396	58	214	1.1	0.000877796	0.00934933
regulation of cell cycle process	biological process	GO:0010564	37	118	1.28	0.000877863	0.00934933
regulation of protein serine/threonine kinase activity	biological process	GO:0071900	34	105	1.32	0.000882847	0.009383757
transferase complex	cellular component	GO:1990234	59	219	1.1	0.000899978	0.009539839
Golgi apparatus	cellular component	GO:0005794	76	301	1.03	0.000901094	0.009539839
nucleoside phosphate metabolic process	biological process	GO:0006753	45	154	1.19	0.000905426	0.009566802
regulation of cell adhesion	biological process	GO:0030155	41	136	1.23	0.00090909	0.009586603
ion transport	biological process	GO:0006811	85	346	1	0.00094228	0.009917083
activation of immune response	biological process	GO:0002253	23	60	1.56	0.000977037	0.010262715
myelin sheath	cellular component	GO:0043209	24	64	1.53	0.000980747	0.010281535
substrate-specific transporter activity	Undef	GO:0022892	81	327	1.01	0.001001093	0.010474328
carbohydrate derivative biosynthetic process	biological process	GO:1901137	53	192	1.12	0.00101313	0.010571209
positive regulation of organelle organization	biological process	GO:0010638	39	128	1.24	0.001014299	0.010571209
Golgi apparatus part	cellular component	GO:0044431	48	169	1.16	0.001036703	0.010783722

protein modification by small protein conjugation	biological process	GO:0032446	54	197	1.12	0.001044179	0.010819474
hemopoiesis	biological process	GO:0030097	54	197	1.12	0.001044179	0.010819474
leukocyte activation	biological process	GO:0045321	46	160	1.17	0.001058064	0.010942184
regulation of gene expression, epigenetic	biological process	GO:0040029	19	45	1.72	0.001068722	0.011031108
carboxylic acid metabolic process	biological process	GO:0019752	55	202	1.11	0.001073978	0.011064042
ncRNA metabolic process	biological process	GO:0034660	40	133	1.23	0.001081067	0.011115691
regulation of neuron projection development	biological process	GO:0010975	26	73	1.45	0.001137562	0.011674171
actin polymerization or depolymerization	biological process	GO:0008154	21	53	1.61	0.001142917	0.011706706
lymphocyte activation	biological process	GO:0046649	41	138	1.21	0.001146536	0.011721365
response to abiotic stimulus	biological process	GO:0009628	58	217	1.09	0.001155755	0.011793106
positive regulation of phosphate metabolic process	biological process	GO:0045937	63	241	1.07	0.001163449	0.011826559
positive regulation of phosphorus metabolic process	biological process	GO:0010562	63	241	1.07	0.001163449	0.011826559
oxoacid metabolic process	biological process	GO:0043436	55	203	1.1	0.001179593	0.011967952
plasma membrane part	cellular component	GO:0044459	123	543	0.92	0.001200982	0.012161933
protein kinase binding	molecular function	GO:0019901	33	103	1.31	0.001205306	0.012182691
sphingolipid metabolic process	biological process	GO:0006665	15	31	1.97	0.001225074	0.012359173
regulation of catabolic process	biological process	GO:0009894	35	112	1.27	0.001248427	0.012565106
positive regulation of response to external stimulus	biological process	GO:0032103	18	42	1.75	0.001252523	0.012565106
condensed chromosome	cellular component	GO:0000793	18	42	1.75	0.001252523	0.012565106
ceramide metabolic process	biological process	GO:0006672	12	21	2.33	0.001280089	0.01281764
positive regulation of protein modification process	biological process	GO:0031401	65	252	1.05	0.001302485	0.013017559
actin filament polymerization	biological process	GO:0030041	19	46	1.68	0.001311855	0.013086789
positive regulation of gene expression	biological process	GO:0010628	93	391	0.97	0.001322574	0.013169195
actin cytoskeleton organization	biological process	GO:0030036	44	153	1.17	0.001333256	0.013250934
single organismal cell-cell adhesion	Undef	GO:0016337	47	167	1.15	0.001357802	0.013382678
membrane region	cellular component	GO:0098589	25	70	1.46	0.001358642	0.013382678
nucleobase-containing compound catabolic process	biological process	GO:0034655	25	70	1.46	0.001358642	0.013382678
erythrocyte homeostasis	biological process	GO:0034101	16	35	1.86	0.001358654	0.013382678
lysosomal transport	biological process	GO:0007041	11	18	2.49	0.001359003	0.013382678
DNA recombination	biological process	GO:0006310	24	66	1.48	0.001377697	0.013520643

immune response-activating signal transduction	biological process	GO:0002757	21	54	1.58	0.001378061	0.013520643
positive regulation of cell activation	biological process	GO:0050867	22	58	1.55	0.001389201	0.013605031
transmembrane transporter activity	molecular function	GO:0022857	74	297	1.02	0.001408618	0.01377001
peptidyl-amino acid modification	biological process	GO:0018193	76	307	1.01	0.001421323	0.013868902
cell morphogenesis	biological process	GO:0000902	78	317	1	0.001430575	0.013933801
negative regulation of cell proliferation	biological process	GO:0008285	39	131	1.21	0.001443837	0.01403745
actin filament-based process	biological process	GO:0030029	46	163	1.15	0.001448255	0.014054895
regulation of transport	biological process	GO:0051049	86	358	0.98	0.001539073	0.014905523
negative regulation of cell migration	biological process	GO:0030336	18	43	1.71	0.001544253	0.014905523
endopeptidase regulator activity	molecular function	GO:0061135	18	43	1.71	0.001544253	0.014905523
chromatin silencing	biological process	GO:0006342	13	25	2.12	0.001558904	0.015019869
erythrocyte differentiation	biological process	GO:0030218	15	32	1.91	0.001566019	0.01503434
DNA-dependent DNA replication	biological process	GO:0006261	15	32	1.91	0.001566019	0.01503434
organic acid metabolic process	biological process	GO:0006082	57	216	1.08	0.001617072	0.0154967
regulation of actin filament length	biological process	GO:0030832	20	51	1.6	0.001635873	0.015565491
regulation of cysteine-type endopeptidase activity	biological process	GO:2000116	20	51	1.6	0.001635873	0.015565491
regulation of cysteine-type endopeptidase activity involved in apoptotic process	biological process	GO:0043281	20	51	1.6	0.001635873	0.015565491
regulation of actin polymerization or depolymerization	biological process	GO:0008064	20	51	1.6	0.001635873	0.015565491
transmembrane transport	biological process	GO:0055085	86	359	0.98	0.001646842	0.015642079
nucleoside-triphosphatase regulator activity	molecular function	GO:0060589	22	59	1.52	0.001655383	0.015695378
hematopoietic or lymphoid organ development	biological process	GO:0048534	56	212	1.08	0.001733014	0.016402395
microtubule cytoskeleton organization	biological process	GO:0000226	33	106	1.27	0.001777316	0.016792036
regulation of anatomical structure size	biological process	GO:0090066	41	142	1.18	0.001789185	0.016874412
negative regulation of cell adhesion	biological process	GO:0007162	17	40	1.73	0.001814651	0.01708451
substrate-specific transmembrane transporter activity	Undef	GO:0022891	67	266	1.03	0.001831737	0.017215111
secretion by cell	biological process	GO:0032940	51	189	1.1	0.001864083	0.017457859
protein localization to organelle	biological process	GO:0033365	51	189	1.1	0.001864083	0.017457859
regulation of actin filament-based process	biological process	GO:0032970	29	89	1.33	0.001876929	0.017547488
regulation of actin filament polymerization	biological process	GO:0030833	18	44	1.67	0.00189139	0.017651719

zinc ion binding	molecular function	GO:0008270	74	301	1	0.001894668	0.017651719
response to wounding	biological process	GO:0009611	34	111	1.25	0.001909875	0.017762498
positive regulation of phosphorylation	biological process	GO:0042327	57	218	1.07	0.001923773	0.01786075
Ras protein signal transduction	biological process	GO:0007265	23	64	1.46	0.001940109	0.017981249
negative regulation of protein phosphorylation	biological process	GO:0001933	31	98	1.29	0.001961639	0.018143525
GTPase regulator activity	molecular function	GO:0030695	20	52	1.57	0.001967395	0.018143525
regulation of mitotic cell cycle phase transition	biological process	GO:1901990	21	56	1.53	0.001973895	0.018143525
nuclease activity	molecular function	GO:0004518	21	56	1.53	0.001973895	0.018143525
microtubule motor activity	molecular function	GO:0003777	10	16	2.55	0.001977939	0.018143525
lipid particle	cellular component	GO:0005811	10	16	2.55	0.001977939	0.018143525
response to biotic stimulus	biological process	GO:0009607	50	185	1.1	0.001995639	0.018274594
cellular component morphogenesis	biological process	GO:0032989	83	347	0.97	0.002029471	0.018552689
response to endogenous stimulus	biological process	GO:0009719	79	327	0.98	0.002042488	0.01863988
wound healing	biological process	GO:0042060	30	94	1.3	0.002055988	0.018699367
regulation of cell activation	biological process	GO:0050865	30	94	1.3	0.002055988	0.018699367
positive regulation of apoptotic signaling pathway	biological process	GO:2001235	16	37	1.76	0.002127706	0.019318849
positive regulation of cellular component movement	biological process	GO:0051272	31	99	1.28	0.002231824	0.020066231
innate immune response	biological process	GO:0045087	31	99	1.28	0.002231824	0.020066231
positive regulation of locomotion	biological process	GO:0040017	31	99	1.28	0.002231824	0.020066231
reactive oxygen species metabolic process	biological process	GO:0072593	17	41	1.69	0.002232495	0.020066231
sister chromatid segregation	biological process	GO:0000819	17	41	1.69	0.002232495	0.020066231
regulation of innate immune response	biological process	GO:0045088	17	41	1.69	0.002232495	0.020066231
positive regulation of intracellular signal transduction	biological process	GO:1902533	52	196	1.08	0.002286499	0.020516502
regulation of DNA replication	biological process	GO:0006275	12	23	2.13	0.00229025	0.020516502
negative regulation of cell motility	biological process	GO:2000146	18	45	1.63	0.002301895	0.020586399
lysosomal membrane	cellular component	GO:0005765	19	49	1.58	0.002340815	0.020735284
vacuole organization	biological process	GO:0007033	19	49	1.58	0.002340815	0.020735284
peptidase regulator activity	molecular function	GO:0061134	19	49	1.58	0.002340815	0.020735284
lytic vacuole membrane	cellular component	GO:0098852	19	49	1.58	0.002340815	0.020735284
contractile fiber	cellular component	GO:0043292	21	57	1.5	0.002345638	0.020735284

negative regulation of endopeptidase activity	biological process	GO:0010951	21	57	1.5	0.002345638	0.020735284
immune response-regulating signaling pathway	biological process	GO:0002764	21	57	1.5	0.002345638	0.020735284
poly(A) RNA binding	Undef	GO:0044822	74	304	0.99	0.002350721	0.020740368
microtubule-based movement	biological process	GO:0007018	20	53	1.54	0.002353956	0.020740368
positive regulation of protein phosphorylation	biological process	GO:0001934	55	211	1.06	0.002410407	0.021202875
vesicle organization	biological process	GO:0016050	25	74	1.38	0.002516004	0.022095468
regulation of cytokine production	biological process	GO:0001817	31	100	1.26	0.002533334	0.022211242
regulation of anatomical structure morphogenesis	biological process	GO:0022603	54	207	1.06	0.002585428	0.022630943
myeloid cell homeostasis	biological process	GO:0002262	16	38	1.72	0.002631039	0.022963644
activation of innate immune response	biological process	GO:0002218	13	27	1.96	0.00263201	0.022963644
regulation of actin cytoskeleton organization	biological process	GO:0032956	27	83	1.33	0.002687935	0.023413448
vasculature development	biological process	GO:0001944	44	160	1.12	0.002725045	0.02369816
protein heterooligomerization	biological process	GO:0051291	10	17	2.4	0.002745843	0.023840326
developmental growth involved in morphogenesis	biological process	GO:0060560	18	46	1.59	0.002784441	0.024136329
magnesium ion binding	molecular function	GO:0000287	20	54	1.51	0.002802489	0.024179124
protein localization to membrane	biological process	GO:0072657	29	92	1.28	0.002802851	0.024179124
gliogenesis	biological process	GO:0042063	19	50	1.55	0.002807432	0.024179124
tRNA metabolic process	biological process	GO:0006399	19	50	1.55	0.002807432	0.024179124
endosome to lysosome transport	biological process	GO:0008333	9	14	2.62	0.002879338	0.024718936
myelination	biological process	GO:0042552	9	14	2.62	0.002879338	0.024718936
negative regulation of intracellular signal transduction	biological process	GO:1902532	33	110	1.22	0.002894547	0.024809742
ligase activity	molecular function	GO:0016874	25	75	1.36	0.002910404	0.024905808
single-organism carbohydrate metabolic process	Undef	GO:0044723	41	147	1.14	0.003015955	0.025767892
positive regulation of cell motility	biological process	GO:2000147	30	97	1.26	0.003026045	0.025812936
purine-containing compound metabolic process	biological process	GO:0072521	36	124	1.18	0.00303573	0.025854383
guanyl-nucleotide exchange factor activity	molecular function	GO:0005085	15	35	1.75	0.00309454	0.026230142
peptidyl-tyrosine dephosphorylation	biological process	GO:0035335	15	35	1.75	0.00309454	0.026230142
positive regulation of innate immune response	biological process	GO:0045089	15	35	1.75	0.00309454	0.026230142
blood vessel development	biological process	GO:0001568	42	152	1.13	0.003117196	0.026380441
regulation of protein localization	biological process	GO:0032880	51	195	1.07	0.003191452	0.026966258
developmental growth	biological process	GO:0048589	40	143	1.14	0.003222395	0.027184839

cation transport	biological process	GO:0006812	60	239	1.02	0.003247884	0.027356786
negative regulation of peptidase activity	biological process	GO:0010466	21	59	1.45	0.003267899	0.02748216
cellular amide metabolic process	biological process	GO:0043603	63	254	1.01	0.003301156	0.027718327
positive regulation of leukocyte activation	biological process	GO:0002696	20	55	1.48	0.003320463	0.02783681
regulation of protein localization to nucleus	biological process	GO:1900180	18	47	1.56	0.00334841	0.027990811
GTPase activator activity	molecular function	GO:0005096	19	51	1.52	0.003349283	0.027990811
regulation of cellular catabolic process	biological process	GO:0031329	25	76	1.34	0.003355845	0.027993826
positive regulation of protein polymerization	biological process	GO:0032273	13	28	1.89	0.003360253	0.027993826
nucleoplasm part	cellular component	GO:0044451	49	186	1.07	0.003365321	0.027993826
DNA-dependent ATPase activity	molecular function	GO:0008094	11	21	2.13	0.003373525	0.028018561
cytoplasmic vesicle	cellular component	GO:0031410	57	225	1.03	0.003433895	0.028431803
intracellular vesicle	cellular component	GO:0097708	57	225	1.03	0.003433895	0.028431803
cytokine production	biological process	GO:0001816	32	107	1.22	0.003449772	0.028475239
negative regulation of phosphorylation	biological process	GO:0042326	32	107	1.22	0.003449772	0.028475239
neurogenesis	biological process	GO:0022008	81	345	0.96	0.003456867	0.0284899
positive regulation of multicellular organismal process	biological process	GO:0051240	73	305	0.98	0.003568107	0.029360737
cytoplasmic, membrane-bounded vesicle	Undef	GO:0016023	53	206	1.05	0.003573493	0.029360737
regulation of DNA metabolic process	biological process	GO:0051052	23	68	1.38	0.003614273	0.029650319
response to lipid	biological process	GO:0033993	41	149	1.12	0.003679086	0.030135879
condensed chromosome kinetochore	cellular component	GO:0000777	10	18	2.26	0.003734365	0.030541972
cell-cell signaling	biological process	GO:0007267	76	321	0.96	0.00377734	0.030846354
single-organism intracellular transport	Undef	GO:1902582	39	140	1.13	0.00381036	0.031068645
protein tyrosine phosphatase activity	molecular function	GO:0004725	15	36	1.7	0.003817826	0.031082207
leukocyte migration	biological process	GO:0050900	21	60	1.43	0.00383228	0.031152538
anatomical structure homeostasis	biological process	GO:0060249	25	77	1.32	0.003857362	0.031308919
motor activity	molecular function	GO:0003774	12	25	1.96	0.00387103	0.031332133
negative regulation of catalytic activity	biological process	GO:0043086	47	178	1.08	0.003871919	0.031332133
secretion	biological process	GO:0046903	53	207	1.04	0.00388114	0.031359378
DNA-templated transcription, initiation	biological process	GO:0006352	16	40	1.63	0.003935073	0.031699527
endopeptidase inhibitor activity	molecular function	GO:0004866	16	40	1.63	0.003935073	0.031699527

negative regulation of mitotic cell cycle	biological process	GO:0045930	18	48	1.53	0.004003891	0.032054443
regulation of cell growth	biological process	GO:0001558	27	86	1.28	0.00401167	0.032054443
proteasome-mediated ubiquitin-dependent protein catabolic process	biological process	GO:0043161	27	86	1.28	0.00401167	0.032054443
regulation of lymphocyte activation	biological process	GO:0051249	24	73	1.34	0.004023254	0.032054443
regulation of cell morphogenesis involved in differentiation	biological process	GO:0010769	24	73	1.34	0.004023254	0.032054443
intercellular bridge	cellular component	GO:0045171	9	15	2.44	0.004032984	0.032054443
phosphatidylinositol-3-phosphate binding	molecular function	GO:0032266	9	15	2.44	0.004032984	0.032054443
axon ensheathment	biological process	GO:0008366	9	15	2.44	0.004032984	0.032054443
ensheathment of neurons	biological process	GO:0007272	9	15	2.44	0.004032984	0.032054443
positive regulation of cell migration	biological process	GO:0030335	29	95	1.24	0.004092098	0.032476103
homeostasis of number of cells	biological process	GO:0048872	23	69	1.36	0.004183924	0.033104589
negative regulation of proteolysis	biological process	GO:0045861	23	69	1.36	0.004183924	0.033104589
spindle localization	biological process	GO:0051653	8	12	2.72	0.004189829	0.033104589
regulation of growth	biological process	GO:0040008	39	141	1.13	0.004211418	0.033226169
negative regulation of transcription from RNA polymerase II promoter	biological process	GO:0000122	47	179	1.07	0.00422918	0.033317229
midbody	cellular component	GO:0030496	13	29	1.83	0.004243877	0.03338392
protein serine/threonine kinase activity	molecular function	GO:0004674	34	118	1.17	0.004284245	0.033602782
autophagy	biological process	GO:0006914	34	118	1.17	0.004284245	0.033602782
myeloid cell differentiation	biological process	GO:0030099	30	100	1.22	0.004360831	0.034120539
endoplasmic reticulum part	cellular component	GO:0044432	49	189	1.06	0.004362996	0.034120539
basement membrane	cellular component	GO:0005604	11	22	2.04	0.004420242	0.03451784
regulation of wound healing	biological process	GO:0061041	14	33	1.73	0.004505919	0.035135672
regulation of leukocyte activation	biological process	GO:0002694	27	87	1.26	0.004559766	0.035503873
regulation of neurogenesis	biological process	GO:0050767	39	142	1.12	0.004647838	0.036137106
endoplasmic reticulum membrane	cellular component	GO:0005789	43	161	1.09	0.004682187	0.036351413
chromosome, telomeric region	cellular component	GO:0000781	16	41	1.59	0.004762554	0.036921858
nuclear outer membrane-endoplasmic reticulum membrane network	cellular component	GO:0042175	44	166	1.08	0.004779938	0.037003078
regulation of hemopoiesis	biological process	GO:1903706	26	83	1.28	0.004802884	0.037089923
positive regulation of cell differentiation	biological process	GO:0045597	50	195	1.04	0.004805004	0.037089923

regulation of body fluid levels	biological process	GO:0050878	23	70	1.34	0.004826539	0.037202548
side of membrane	cellular component	GO:0098552	28	92	1.24	0.004889749	0.03763561
phagocytosis	biological process	GO:0006909	12	26	1.88	0.004938165	0.037845138
pattern recognition receptor signaling pathway	biological process	GO:0002221	12	26	1.88	0.004938165	0.037845138
innate immune response-activating signal transduction	biological process	GO:0002758	12	26	1.88	0.004938165	0.037845138
carbohydrate metabolic process	biological process	GO:0005975	47	181	1.06	0.005028877	0.038485278
cellular response to endogenous stimulus	biological process	GO:0071495	67	280	0.97	0.005085782	0.038842635
regulation of cell morphogenesis	biological process	GO:0022604	33	115	1.17	0.005090075	0.038842635
nitrogen compound transport	biological process	GO:0071705	43	162	1.08	0.005128149	0.039077519
organophosphate biosynthetic process	biological process	GO:0090407	36	129	1.14	0.005153048	0.039211476
extrinsic component of membrane	cellular component	GO:0019898	24	75	1.3	0.005299861	0.040095356
organic acid biosynthetic process	biological process	GO:0016053	24	75	1.3	0.005299861	0.040095356
nuclear-transcribed mRNA catabolic process	biological process	GO:0000956	13	30	1.77	0.005305568	0.040095356
neuron projection extension	biological process	GO:1990138	13	30	1.77	0.005305568	0.040095356
ribose phosphate metabolic process	biological process	GO:0019693	34	120	1.15	0.005306628	0.040095356
ERK1 and ERK2 cascade	biological process	GO:0070371	20	58	1.4	0.005373712	0.040545034
negative regulation of cell differentiation	biological process	GO:0045596	41	153	1.09	0.005385026	0.040573252
positive regulation of developmental process	biological process	GO:0051094	64	266	0.98	0.00548045	0.041234225
proton-transporting two-sector ATPase complex	cellular component	GO:0016469	9	16	2.29	0.005518037	0.041291725
regulation of ERK1 and ERK2 cascade	biological process	GO:0070372	19	54	1.43	0.005518924	0.041291725
neuron death	biological process	GO:0070997	19	54	1.43	0.005518924	0.041291725
myofibril	cellular component	GO:0030016	19	54	1.43	0.005518924	0.041291725
actin filament binding	molecular function	GO:0051015	14	34	1.68	0.005544856	0.041370188
T cell proliferation	biological process	GO:0042098	14	34	1.68	0.005544856	0.041370188
hemostasis	biological process	GO:0007599	18	50	1.47	0.005633166	0.041912319
contractile fiber part	cellular component	GO:0044449	18	50	1.47	0.005633166	0.041912319
negative regulation of developmental process	biological process	GO:0051093	50	197	1.03	0.005657896	0.041913917
regulation of neuron differentiation	biological process	GO:0045664	33	116	1.16	0.005666028	0.041913917
regulation of response to wounding	biological process	GO:1903034	15	38	1.61	0.005678377	0.041913917
meiotic cell cycle	biological process	GO:0051321	15	38	1.61	0.005678377	0.041913917

blood vessel morphogenesis	biological process	GO:0048514	36	130	1.13	0.005699966	0.041913917
peptidase activity	molecular function	GO:0008233	44	168	1.07	0.005708315	0.041913917
negative regulation of inflammatory response	biological process	GO:0050728	11	23	1.95	0.005709104	0.041913917
alpha-amino acid biosynthetic process	biological process	GO:1901607	11	23	1.95	0.005709104	0.041913917
proton-transporting V-type ATPase complex	cellular component	GO:0033176	6	7	3.49	0.005711622	0.041913917
hydrogen-exporting ATPase activity	molecular function	GO:0036442	6	7	3.49	0.005711622	0.041913917
peptidase inhibitor activity	molecular function	GO:0030414	16	42	1.55	0.005726387	0.04196478
angiogenesis	biological process	GO:0001525	31	107	1.18	0.005783387	0.042324592
regulation of homeostatic process	Undef	GO:0032844	27	89	1.24	0.005845182	0.042660276
regulation of apoptotic signaling pathway	biological process	GO:2001233	27	89	1.24	0.005845182	0.042660276
positive regulation of RNA metabolic process	biological process	GO:0051254	76	328	0.94	0.005900921	0.043008482
early endosome	cellular component	GO:0005769	21	63	1.36	0.006031393	0.043899688
fatty acid metabolic process	biological process	GO:0006631	24	76	1.29	0.006054426	0.044007546
production of molecular mediator involved in inflammatory response	biological process	GO:0002532	7	10	2.85	0.006086909	0.044064287
negative regulation of cell-substrate adhesion	biological process	GO:0010812	7	10	2.85	0.006086909	0.044064287
protein heterotetramerization	biological process	GO:0051290	7	10	2.85	0.006086909	0.044064287
organelle inner membrane	cellular component	GO:0019866	35	126	1.13	0.006099826	0.044098201
cellular homeostasis	biological process	GO:0019725	43	164	1.07	0.006128826	0.044248139
anatomical structure formation involved in morphogenesis	biological process	GO:0048646	69	293	0.96	0.006164446	0.044445403
actin filament organization	biological process	GO:0007015	28	94	1.21	0.006212289	0.044730149
protein maturation	biological process	GO:0051604	20	59	1.38	0.006254719	0.044975206
response to other organism	biological process	GO:0051707	45	174	1.05	0.006315969	0.045294038
response to external biotic stimulus	biological process	GO:0043207	45	174	1.05	0.006315969	0.045294038
single organism reproductive process	Undef	GO:0044702	46	179	1.05	0.006396117	0.045807482
regulation of macroautophagy	biological process	GO:0016241	10	20	2.04	0.006540261	0.046590663
spindle assembly	biological process	GO:0051225	10	20	2.04	0.006540261	0.046590663
DNA duplex unwinding	biological process	GO:0032508	10	20	2.04	0.006540261	0.046590663
myeloid cell development	biological process	GO:0061515	10	20	2.04	0.006540261	0.046590663
cell projection organization	biological process	GO:0030030	69	294	0.96	0.006577203	0.046791604
cell projection	cellular component	GO:0042995	77	335	0.94	0.006614597	0.046995218

purine ribonucleotide metabolic process	biological process	GO:0009150	32	113	1.15	0.006727237	0.047732197
regulation of small GTPase mediated signal transduction	biological process	GO:0051056	17	47	1.47	0.006763276	0.047766169
myeloid leukocyte migration	biological process	GO:0097529	14	35	1.63	0.006767691	0.047766169
mitotic sister chromatid segregation	biological process	GO:0000070	14	35	1.63	0.006767691	0.047766169
mRNA catabolic process	biological process	GO:0006402	14	35	1.63	0.006767691	0.047766169
positive regulation of transcription from RNA polymerase II promoter	biological process	GO:0045944	60	249	0.98	0.00679278	0.047880162
tissue homeostasis	biological process	GO:0001894	16	43	1.52	0.006842161	0.048164855
ribonucleotide metabolic process	biological process	GO:0009259	33	118	1.14	0.006982652	0.049089324
neuron projection	cellular component	GO:0043005	41	156	1.07	0.007066812	0.049615874

Table S7 The significantly enriched pathways in the comparison of shRIP2 vs. WT with adjusted *p* _value ≤0.05

Term	Database	ID	Input number	Background number	Rich factor	*p* value	Corrected *p* value
Metabolic pathways	KEGG	gga01100	168	1092	1.13	2.06E-10	3.03E-08
Phagosome	KEGG	gga04145	32	130	1.81	2.73E-06	0.000200941
Lysosome	KEGG	gga04142	28	109	1.88	5.68E-06	0.000278113
AGE-RAGE signaling pathway in diabetic complications	KEGG	gga04933	22	89	1.81	8.89E-05	0.002795131
Purine metabolism	KEGG	gga00230	31	154	1.48	0.00011	0.002795131
MAPK signaling	KEGG	gga04010	39	223	1.32	0.000114	0.002795131
Cytokine-cytokine receptor interaction	KEGG	gga04060	33	171	1.42	0.000135	0.002839434
Apoptosis	KEGG	gga04210	26	125	1.53	0.000244	0.004489116
VEGF signaling [illegible]	KEGG	gga04370	15	57	1.93	0.00065	0.010614899
ECM-receptor [illegible]	KEGG	gga04512	17	74	1.69	0.001087	0.015984861
Metabolism of xenobiotics by cytochrome P450	KEGG	gga00980	10	32	2.29	0.001756	0.023472017
Focal adhesion	KEGG	gga04510	31	187	1.22	0.001926	0.023592666
Pyruvate metabolism	KEGG	gga00620	10	34	2.16	0.002548	0.02880937
Fatty acid biosynthesis	KEGG	gga00061	6	13	3.39	0.003249	0.034112348
Herpes simplex infection	KEGG	gga05168	25	150	1.22	0.004694	0.046004837

Table S8 The significantly enriched pathways in the comparison of shRIP2+APEC vs. APEC with adjusted *p* -value ≤ 0.05

Term	Database	ID	Input number	Background number	Rich factor	*p* value	Corrected *p* value
Metabolic pathways	KEGG	gga01100	328	1092	1.22	2.29E-20	3.48E-18
Phagosome	KEGG	gga04145	65	130	2.04	1.06E-11	8.07E-10
Focal adhesion	KEGG	gga04510	66	187	1.44	4.41E-07	2.23E-05
Oocyte meiosis	KEGG	gga04114	41	92	1.82	6.93E-07	2.42E-05
AGE-RAGE signaling pathway in diabetic	KEGG	gga04933	40	89	1.83	7.96E-07	2.42E-05
Apoptosis	KEGG	gga04210	48	125	1.56	2.48E-06	6.29E-05
ECM-receptor interaction	KEGG	gga04512	33	74	1.82	8.03E-06	0.0001743
Insulin signaling pathway	KEGG	gga04910	45	121	1.52	9.73E-06	0.0001848
Regulation of actin	KEGG	gga04810	60	185	1.32	1.23E-05	0.0002083
Herpes simplex infection	KEGG	gga05168	51	150	1.39	1.93E-05	0.0002939
Phosphatidylinositol signaling system	KEGG	gga04070	36	91	1.61	2.56E-05	0.0003542
MAPK signaling pathway	KEGG	gga04010	67	223	1.22	3.03E-05	0.0003839
Purine metabolism	KEGG	gga00230	51	154	1.35	3.41E-05	0.0003988
Influenza A	KEGG	gga05164	47	138	1.39	3.87E-05	0.0004204
Salmonella infection	KEGG	gga05132	29	68	1.74	5.25E-05	0.0005321
Fatty acid metabolism	KEGG	gga01212	21	42	2.04	9.93E-05	0.0009335
Pyrimidine metabolism	KEGG	gga00240	35	95	1.5	0.000104	0.0009335
Inositol phosphate metabolism	KEGG	gga00562	28	69	1.65	0.000138	0.0011553
Progesterone-mediated oocyte maturation	KEGG	gga04914	30	77	1.59	0.000144	0.0011553
Adrenergic signaling in cardiomyocytes	KEGG	gga04261	41	123	1.36	0.00017	0.0012905
p53 signaling pathway	KEGG	gga04115	26	64	1.66	0.000231	0.0016736
Steroid biosynthesis	KEGG	gga00100	12	17	2.88	0.000324	0.0021871
Cell cycle	KEGG	gga04110	38	115	1.35	0.000331	0.0021871
Calcium signaling pathway	KEGG	gga04020	48	161	1.21	0.000433	0.0027443
Lysosome	KEGG	gga04142	36	109	1.35	0.000471	0.0027881
Tight junction	KEGG	gga04530	39	122	1.3	0.000477	0.0027881
Endocytosis	KEGG	gga04144	64	238	1.1	0.000586	0.003297
Toll-like receptor signaling pathway	KEGG	gga04620	30	87	1.4	0.000763	0.0041425
Terpenoid backbone biosynthesis	KEGG	gga00900	12	20	2.44	0.000934	0.0048966
RNA degradation	KEGG	gga03018	24	67	1.46	0.001623	0.0082245
One carbon pool by folate	KEGG	gga00670	10	16	2.55	0.001978	0.0096391
GnRH signaling pathway	KEGG	gga04912	27	81	1.36	0.002029	0.0096391
VEGF signaling pathway	KEGG	gga04370	21	57	1.5	0.002346	0.0108042
FoxO signaling pathway	KEGG	gga04068	37	127	1.19	0.002556	0.0114253
Aminoacyl-tRNA biosynthesis	KEGG	gga00970	17	42	1.65	0.002728	0.0118462
Vascular smooth muscle contraction	KEGG	gga04270	31	103	1.23	0.003655	0.0154335
Glycerophospholipid	KEGG	gga00564	28	91	1.25	0.004322	0.0177571
mTOR signaling pathway	KEGG	gga04150	39	142	1.12	0.004648	0.0185914
RNA transport	KEGG	gga03013	37	133	1.13	0.004819	0.0187799
Gap junction	KEGG	gga04540	25	81	1.26	0.006535	0.0248343
NOD-like receptor signaling pathway	KEGG	gga04621	16	43	1.52	0.006842	0.0253661
Spliceosome	KEGG	gga03040	31	109	1.16	0.007186	0.026008

Caffeine metabolism	KEGG	gga00232	5	5	4.07	0.007783	0.0275121
N-Glycan biosynthesis	KEGG	gga00510	17	48	1.44	0.007973	0.0275421
Biosynthesis of amino acids	KEGG	gga01230	20	61	1.34	0.008371	0.027634
Adherens junction	KEGG	gga04520	22	70	1.28	0.008719	0.027634
Synthesis and degradation of ketone bodies	KEGG	gga00072	7	11	2.59	0.008755	0.027634
TGF-beta signaling pathway	KEGG	gga04350	24	79	1.24	0.008866	0.027634
Protein processing in endoplasmic reticulum	KEGG	gga04141	39	149	1.07	0.008908	0.027634
Pantothenate and CoA biosynthesis	KEGG	gga00770	8	15	2.17	0.011012	0.0334773
Glycosaminoglycan biosynthesis - chondroitin sulfate / dermatan sulfate	KEGG	gga00532	9	19	1.93	0.012549	0.0373995
Proteasome	KEGG	gga03050	13	35	1.51	0.014168	0.0406342
Intestinal immune network for IgA production	KEGG	gga04672	13	35	1.51	0.014168	0.0406342
Notch signaling pathway	KEGG	gga04330	15	44	1.39	0.015899	0.0447515
Fatty acid biosynthesis	KEGG	gga00061	7	13	2.19	0.016497	0.0455923

Table S9 The differentially expressed genes involved in the MAPK signaling pathway in the comparison of shRIP2 vs. WT

Gene Name	Fold change	*p* value	FDR	Ensembl ID	uniprot ID	uniprot Description
PDGFA	4.67947149	5.2399E-12	3.0329E-10	-	A0A226NJI1	Platelet derived growth factor subunit A
IL1R2	3.53545031	3.0106E-17	3.8095E-14	ENSGALG00000046326	A0A1L1RVR1	Interleukin 1 receptor type 2
DUSP1	2.97839881	1.9846E-11	8.8042E-10	ENSGALG00000040360	A0A1D5P463	Dual specificity protein phosphatase 1
ARR3	2.95823754	6.209E-09	9.4391E-08	ENSGALG00000004251	A0A1D5PEW5	Arrestin 3
IL1B	2.8744811	7.5469E-08	7.6121E-07	-	A3DTN2	Interleukin-1
ENSGALG00000026584	2.40106536	6.6966E-05	0.00025059	-	A0A1V4KGL3	Phospholipase A2
DUSP4	2.10119474	7.7314E-05	0.00028425	ENSGALG00000011419	F1NCC5	Dual specificity protein phosphatase 4
FGF2	2.08945573	0.00013889	0.0004743	-	A0A1V4JUL8	Fibroblast growth factor
ENSGALG00000031572	2.036432	0.00962049	0.01921357	ENSGALG00000031572	A0A1D5PQS5	Uncharacterized protein
DUSP3	1.99136506	2.4778E-09	4.3911E-08	-	A0A226NWY8	Dual specificity protein phosphatase 3
MAP3K12	1.87845795	0.00142203	0.00361611	-	A0A1U8DN12	Mitogen-activated protein kinase kinase kinase
DUSP16	1.86173419	2.3655E-07	1.9979E-06	ENSGALG00000028155	R4GM14	Dual specificity protein phosphatase 16
ENSGALG00000031518	1.85821408	1.9983E-12	1.4567E-10	ENSGALG00000031518	A0A0A7U2X8	DNA damage inducible transcript 3
FLNB	1.7305421	1.6407E-12	1.3136E-10	ENSGALG00000005678	A0A1D5NYG3	Filamin B
PLA2G4A	1.70460489	1.3166E-09	2.5325E-08	ENSGALG00000005065	A0A1I7Q3Z2	Phospholipase A2
JUN	1.61177091	3.5141E-08	3.9875E-07	-	A0A226PR05	Jun proto-oncogene, AP-1 transcription factor subunit
MAP3K5	1.59205507	2.284E-09	4.1053E-08	ENSGALG00000013892	A0A1D5PTJ8	Mitogen-activated protein kinase kinase kinase 5

FASLG	1.56854851	0.02083292	0.03780123	ENSGALG00000003076	Q5CAQ0	Fas ligand
ENSGALG00000050166	1.56515325	0.0004958	0.00145016	ENSACAG00000007736	H9GD19	Uncharacterized protein
GADD45A	1.54048086	5.6761E-07	4.1591E-06	ENSGALG00000025977	Q2HZD6	Growth arrest and DNA damage inducible alpha
MAPK13	1.53238479	6.505E-07	4.6321E-06	-	A0A226NYB9	Mitogen-activated protein kinase
RASGRP1	1.50480923	7.0922E-09	1.0491E-07	ENSGALG00000009740	Q5F3V4	RAS guanyl releasing protein 1
RAC2	-1.5054949	2.1489E-06	1.2796E-05	-	A0A218UZY4	Ras-related C3 botulinum toxin substrate 2
CASP3	-1.5125361	6.1107E-07	4.406E-06	ENSGALG00000010638	O93417	Caspase-3
MAPKAPK3	-1.5435219	0.03281314	0.05583766	-	A0A1V4KAD3	MAP kinase-activated protein kinase 3 isoform A
ENSGALG00000008727	-1.5531046	2.9151E-06	1.6629E-05	ENSGALG00000008727	E1BWG9	Phospholipase A2
RASGRP3	-1.6338055	4.3817E-11	1.6261E-09	ENSGALG00000010435	Q5ZID9	RAS guanyl releasing protein 3
TNIK	-1.6809526	1.6017E-08	2.0455E-07	ENSPSIG00000018253	K7GK27	TRAF2 and NCK interacting kinase
FGF1	-1.7369471	0.00755392	0.0155617	-	A0A226PB16	Fibroblast growth factor 1
TGFBR2	-1.7496572	8.773E-07	5.9934E-06	ENSGALG00000011442	A0A1I7Q417	Transforming growth factor beta receptor 2
RPS6KA1	-2.0006536	1.7781E-09	3.2849E-08	ENSGALG00000037136	A0A1D5PCA5	Ribosomal protein S6 kinase
NRK	-2.0543877	3.4372E-12	2.2242E-10	-	Q5F454	Nik related kinase
HSPB1	-2.488179	0.0133596	0.02566557	ENSGALG00000001926	F1P593	Heat shock protein beta-1
CACNG1	-2.493277	3.1186E-05	0.0001278	ENSGALG00000027771	F1NC88	Voltage-dependent calcium channel gamma-1 subunit

RASGRF1	-2.5590592	2.8416E-11	1.1656E-09	ENSGALG00000008262	F1P3U7	Ras protein specific guanine nucleotide releasing factor 1
CACNB2	-2.565841	4.5625E-06	2.4581E-05	ENSGALG00000008591	E1BZF6	Calcium voltage-gated channel auxiliary subunit beta 2
RPS6KA2	-2.7104926	7.5163E-10	1.5886E-08	-	A0A1V4JKY4	Ribosomal protein S6 kinase
ENSGALG00000051520	-3.4475671	0.00962512	0.01921978	ENSGALG00000001926	F1P593	Heat shock protein beta-1
PTPN5	-9.8061023	1.2279E-13	1.684E-11	-	A0A226P264	Tyrosine-protein phosphatase non-receptor type 5

Table S10 The differentially expressed genes involved in the MAPK signaling pathway in the comparison of shRIP2+APEC vs. APEC

Gene Name	Fold change	*p* value	FDR	Ensembl ID	uniprot ID	uniprot Description
ENSGALG00000031518	12.37337026	3.83771E-38	1.64024E-34	ENSGALG00000031518	A0A0A7U2X8	DNA damage inducible transcript 3
NTRK2	5.501087934	5.11174E-09	1.56876E-08	-	A0A0Q3VXM5	Tyrosine-protein kinase receptor
FGF14	5.197322306	1.60325E-08	4.62887E-08	-	Q9IAI5	Fibroblast growth factor
CACNG1	4.708790586	1.00653E-05	2.12299E-05	ENSGALG00000027771	F1NC88	Voltage-dependent calcium channel gamma-1 subunit
ENSGALG00000008727	4.175309609	4.69377E-18	5.07022E-17	ENSGALG00000008727	E1BWG9	Phospholipase A2
RASGRP3	3.725789816	8.54018E-29	9.12518E-27	ENSGALG00000010435	Q5ZID9	RAS guanyl releasing protein 3
PTPRR	3.640601171	1.66105E-10	6.12186E-10	-	A0A1V4K7G7	Receptor-type tyrosine-protein phosphatase R isoform A
CASP3	3.301637291	3.76383E-22	8.80653E-21	ENSGALG00000010638	O93417	Caspase-3
ENSGALG00000026584	2.832679552	8.27831E-06	1.76203E-05	-	A0A1V4KGL3	Phospholipase A2
DUSP5	2.615849366	7.7952E-19	9.61983E-18	ENSGALG00000032618	A0A1D5P5S2	Dual specificity protein phosphatase 5
DUSP3	2.465741047	4.13567E-17	3.83147E-16	-	A0A226NWY8	Dual specificity protein phosphatase 3
RRAS	2.454206353	4.04611E-22	9.36447E-21	-	A0A093EV15	Ras-related protein R-Ras2
GADD45B	2.407377645	4.93972E-18	5.30906E-17	ENSGALG00000029968	A0A1D5NXN3	Growth arrest and DNA damage inducible beta
FGF1	2.216620967	0.000781061	0.001304856	-	A0A226PB16	Fibroblast growth factor 1
IL1B	2.208361358	2.52015E-07	6.38477E-07	-	A3DTN2	Interleukin-1
STMN1	2.046275086	2.04187E-23	6.23354E-22	ENSGALG00000001475	A0A1L1RQ04	Stathmin
DUSP6	1.95574035	1.21905E-16	1.04204E-15	-	Q7T2L9	Dual specificity protein phosphatase 6
DUSP4	1.897087153	3.89761E-05	7.64264E-05	ENSGALG00000011419	F1NCC5	Dual specificity protein phosphatase 4
MKNK1	1.880179081	5.51792E-14	3.26492E-13	ENSGALG00000010440	A0A1D5NT96	MAPK interacting serine/threonine kinase

LOC420419	1.850223512	7.7978E-23	2.10935E-21	ENSGALG00000005884	Q5ZLW4	Mitogen-activated protein kinase kinase kinase 3-like
ATF4	1.819309537	7.3788E-23	2.013E-21	ENSGALG00000012135	Q9W610	ATF4
PPM1A	1.802315003	2.06435E-22	5.05136E-21	ENSMGAG00000013576	G1NLF7	Protein phosphatase, Mg2+/Mn2+ dependent 1A
PTPN5	1.789226918	1.78547E-05	3.65648E-05	-	A0A226P264	Protein tyrosine phosphatase, non-receptor type 5
MAPK9	1.766861738	1.36925E-10	5.08567E-10	-	R0JIP5	Mitogen-activated protein kinase
CACNB3	1.753894566	0.010750142	0.015573192	-	A0A218U974	Voltage-dependent L-type calcium channel subunit beta-3
DUSP8	1.753544064	2.567E-12	1.19211E-11	ENSGALG00000006647	E1C2M2	Dual specificity phosphatase 8
JUN	1.731001898	1.21913E-18	1.43938E-17	-	A0A226PR05	Jun proto-oncogene, AP-1 transcription factor subunit
RAP1B	1.717870529	9.36851E-19	1.13969E-17	ENSAMXG00000000051	W5JXI9	RAP1B, member of RAS oncogene family
FGF18	1.699926064	0.009408516	0.013742993	ENSGALG00000002203	Q9I950	Fibroblast growth factor
CACNA1C	1.676685089	0.000255143	0.000453549	-	A0A1V4K9J4	Voltage-dependent L-type calcium channel subunit alpha
FGF2	1.662740393	0.000639244	0.00108032	-	A0A1V4JUL8	Fibroblast growth factor
KRAS	1.62426655	9.26685E-12	3.98322E-11	ENSMGAG00000013673	G1NLP3	GTPase KRas
H-RAS	1.619695863	1.08948E-15	8.14535E-15	-	A0A226MNN0	HRas proto-oncogene, GTPase
PPP3R1	1.608067246	3.56971E-14	2.16206E-13	-	A0A0S7HHU0	Protein phosphatase 3 regulatory subunit B, alpha
DUSP10	1.564726566	1.80464E-07	4.63987E-07	ENSGALG00000009450	F1NXH3	Dual specificity phosphatase 10
ECSIT	1.524525959	8.3499E-09	2.49214E-08	ENSGALG00000043823	A0A1D5PDN2	ECSIT signalling integrator

MAP2K3	1.515816898	2.03239E-13	1.10565E-12	ENSGALG00000041285	A0A1D5PD64	Mitogen-activated protein kinase kinase 3
RPS6KA3	1.508546931	2.21747E-17	2.14909E-16	-	A0A1V4JC25	Ribosomal protein S6 kinase
NFATC3	-1.49543345	6.04102E-13	3.07862E-12	ENSGALG00000003396	A0A1D5P8S9	Nuclear factor of activated T-cells 3
MAPK12	-1.51782371	4.47278E-12	2.01369E-11	ENSGALG00000019384	F1NLU9	Mitogen-activated protein kinase
ENSGALG00000011502	-1.51789054	1.81496E-09	5.8796E-09	-	A0A226PNH6	Uncharacterized protein
MKNK2	-1.52828404	9.44853E-12	4.05588E-11	ENSGALG00000003845	F1NCK3	MAP kinase interacting serine/threonine kinase 2
MAP3K14	-1.5493441	7.19936E-14	4.1864E-13	ENSMGAG00000010881	G1NEI6	Mitogen-activated protein kinase kinase kinase 14
ENSGALG00000031572	-1.56468859	0.033470482	0.045288996	ENSGALG00000031572	A0A1D5PQS5	Uncharacterized protein
FGFR1	-1.57373148	0.000121925	0.00022549	ENSGALG00000039786	A0A1D5P1S1	Fibroblast growth factor receptor
PDGFB	-1.59190951	1.13185E-10	4.2659E-10	ENSGALG00000012178	F1NE37	Platelet derived growth factor subunit B
GADD45G	-1.60999861	2.72886E-10	9.78177E-10	ENSGALG00000028005	Q2HZD5	Growth arrest and DNA damage inducible gamma
FGFR4	-1.65886886	1.85058E-06	4.26689E-06	ENSGALG00000028543	F1NCL8	Fibroblast growth factor receptor
MAPT	-1.69049824	2.32869E-12	1.08814E-11	-	A0A226MZQ6	Microtubule-associated protein
TAOK3	-1.70004669	5.42971E-20	8.31777E-19	ENSGALG00000007396	F1N9U1	Serine/threonine-protein kinase TAO3
MAPK11	-1.77968715	2.66633E-16	2.15968E-15	ENSGALG00000040023	A0A1D5PRV8	Mitogen-activated protein kinase
IL1R1	-1.85259011	1.23274E-06	2.8949E-06	ENSGALG00000043044	F1P4T7	Interleukin-1 receptor type 1
TNFRSF1A	-1.90456938	9.75777E-18	1.00574E-16	ENSGALG00000039461	Q5ZJG1	TNF receptor superfamily member 1A
CACNA2D2	-1.9318991	1.58675E-11	6.61636E-11	ENSGALG00000038242	A0A1D5PIN9	Calcium voltage-gated channel auxiliary subunit alpha2delta 2

NFATC1	-1.9748422	0.000143923	0.000263739	ENSGALG00000042534	A0A1D5P3J9	Nuclear factor of activated T-cells 1
FOS	-2.06037927	2.98122E-20	4.77219E-19	ENSGALG00000028037	R4GH11	Proto-oncogene c-Fos
TGFBR2	-2.07729972	3.52429E-14	2.13657E-13	ENSGALG00000011442	A0A1I7Q417	Transforming growth factor beta receptor 2
PAK1	-2.21821035	2.50209E-08	7.11192E-08	ENSGALG00000000681	E1C3P6	p21 (RAC1) activated kinase 1
NRK	-2.23769931	2.47519E-27	1.84517E-25	-	Q5F454	Nik related kinase
ENSGALG00000045796	-2.33169592	0.00197033	0.003132883	ENSGALG00000045796	F1N8Q4	Phospholipase A2
RPS6KA1	-2.35716227	2.19714E-21	4.45051E-20	ENSGALG00000037136	A0A1D5PCA5	Ribosomal protein S6 kinase
MAPK14	-2.68054888	1.19252E-22	3.13972E-21	-	A0A218UJ94	Mitogen-activated protein kinase
PDGFRB	-2.69033744	1.03805E-07	2.74092E-07	ENSGALG00000030613	A0A1D5NU66	Platelet derived growth factor receptor beta
HSPB1	-3.85797478	2.19585E-11	8.97805E-11	ENSGALG00000001926	F1P593	Heat shock protein beta-1
PRKCB	-4.78170949	5.44317E-33	3.32344E-30	ENSGALG00000037943	A0A1D5PUY5	Protein kinase C
ENSGALG00000051520	-5.03327933	5.19875E-11	2.04035E-10	ENSGALG00000001926	F1P593	Heat shock protein beta-1
MAP2K6	-7.48693104	1.20682E-09	3.99842E-09	ENSGALG00000004370	F1NVF2	Mitogen-activated protein kinase kinase 6

Table S11 The differentially expressed genes involved in the apoptosis pathway in the comparison of shRIP2 vs. WT

Gene Name	Fold change	*p* value	FDR	Ensembl_ID	uniprot_ID	uniprot_Description
CTSV	2.605609109	3.35172E-15	1.14303E-12	ENSGALG00000012610	F1NYJ1	Cathepsin V
MCL1	2.597399519	2.42014E-15	9.25375E-13	ENSGALG00000044383	A0A1L1RUS7	BCL2 family apoptosis regulator
CTSB	2.008477901	2.78677E-14	5.7423E-12	ENSGALG00000030016	A0A1D5PUR5	Cathepsin B
BCL2L11	1.936629681	0.000188377	0.000620772	-	A0A093GNE7	Bcl-2-like 11
CTSC	1.890922659	2.11408E-11	9.1066E-10	ENSGALG00000017239	F1NWG2	Cathepsin C
ENSGALG00000031518	1.858214081	1.99825E-12	1.45667E-10	ENSGALG00000031518	A0A0A7U2X8	DNA damage inducible transcript 3
PIK3CB	1.79702389	3.20244E-10	7.87689E-09	ENSGALG00000005505	Q5F4A2	Phosphatidylinositol-4,5-bisphosphate 3-kinase catalytic subunit beta
LOC420160	1.710459021	0.002805189	0.006565734	ENSGALG00000004252	F1NZ37	Cathepsin L1-like
TUBA8A	1.684552886	0.000366772	0.001119481	ENSGALG00000013056	F1NMP5	Tubulin alpha 8A
TUB4A	1.674782397	5.04435E-06	2.67998E-05	ENSGALG00000000433	A0A1L1RJ25	Tubulin alpha 4A
PIK3R5	1.633879925	1.12899E-10	3.50876E-09	ENSGALG00000026167	F1NUD8	Phosphoinositide 3-kinase regulatory subunit 5
ENSMGAG00000011055	1.633261749	0.001978513	0.004847119	ENSMGAG00000011055	G1NEY3	Tubulin alpha-3 chain-like
JUN	1.611770906	3.51407E-08	3.98745E-07	-	A0A226PR05	Jun proto-oncogene, AP-1 transcription factor subunit
MAP3K5	1.592055074	2.28395E-09	4.10525E-08	ENSGALG00000013892	A0A1D5PTJ8	Mitogen-activated protein kinase kinase kinase 5
FASLG	1.56854851	0.020832919	0.037801233	ENSGALG00000003076	Q5CAQ0	Fas ligand
TUBA3E	1.558199607	1.45659E-10	4.23485E-09	ENSGALG00000040586	A0A1D5PAR5	Tubulin alpha chain
GADD45A	1.540480862	5.67606E-07	4.15915E-06	ENSGALG00000025977	Q2HZD6	Growth arrest and DNA damage inducible alpha
BCL2L1	1.523653207	8.16708E-10	1.68386E-08	-	A0A226PFT5	Bcl-2-like protein 1
CASP3	-1.512536141	6.11073E-07	4.40601E-06	ENSGALG00000010638	O93417	Caspase-3
SEPTIN4	-1.554203233	0.004589205	0.01006546	-	A0A226MHN0	Septin 4

CASP8	-1.582709453	1.29627E-10	3.86825E-09	ENSGALG00000008355	F1NXP6	Caspase 8
PIK3R1	-1.638611186	1.36641E-09	2.61232E-08	-	A0A226N6Y2	Phosphoinositide-3-kinase regulatory subunit 1
ITPR1	-1.682559179	1.60701E-11	7.45486E-10	-	A0A218URN8	Inositol 1,4,5-trisphosphate receptor type 1
BCL2	-3.561099808	2.09826E-11	9.1066E-10	ENSGALG00000012885	A0A1L1RSE7	Apoptosis regulator Bcl-2
PTPN13	-3.583576225	0.000350327	0.00107266	-	A0A1V4JV72	Tyrosine-protein phosphatase non-receptor type 13 isoform A

Table S12 The differentially expressed genes involved in the apoptosis pathway in the comparison of shRIP2+APEC vs. APEC

Gene Name	Fold change	*p* value	FDR	Ensembl_ID	uniprot ID	uniprot Description
ENSGALG00000031518	12.37337026	3.83771E-38	1.64024E-34	ENSGALG00000031518	A0A0A7U2X8	DNA damage inducible transcript 3
SEPTIN4	4.281612275	7.33422E-08	1.97562E-07	-	A0A226MHN0	Septin 4
CTSV	3.782205648	2.6621E-34	3.10304E-31	ENSGALG00000012610	F1NYJ1	Cathepsin V
ENSGALG00000013056	3.718557464	1.35187E-10	5.03261E-10	ENSGALG00000013056	F1NMP5	Tubulin alpha chain
CASP3	3.301637291	3.76383E-22	8.80653E-21	ENSGALG00000010638	O93417	Caspase-3
CTSK	2.666896457	1.63659E-26	1.02363E-24	ENSGALG00000028147	A0A1D5P0X4	Cathepsin K
TUBA3E	2.444073212	1.72467E-24	6.85963E-23	ENSGALG00000040586	A0A1D5PAR5	Tubulin alpha chain
GADD45B	2.407377645	4.93972E-18	5.30906E-17	ENSGALG00000029968	A0A1D5NXN3	Growth arrest and DNA damage inducible beta
BIRC5	2.302433625	2.40042E-12	1.12002E-11	ENSGALG00000008713	F1NN48	Baculoviral IAP repeat containing 5
CASP7	2.097790406	2.66144E-12	1.23106E-11	ENSGALG00000008933	F1NV61	Caspase 7
CTSD	2.000181803	1.79952E-26	1.11466E-24	-	A0A226N8F2	Cathepsin D
BIRC3	1.993423071	5.69809E-20	8.66677E-19	ENSGALG00000017186	A0A140T8H6	Baculoviral IAP repeat containing 3
PMAIP1	1.984730346	1.20242E-07	3.15155E-07	ENSGALG00000035325	A0A1D5PAR2	Phorbol-12-myristate-13-acetate-induced protein 1
PIK3R3	1.867281863	6.69599E-14	3.91322E-13	ENSGALG00000010335	E1C8M6	Phosphoinositide-3-kinase regulatory subunit 3
DIABLO	1.857182614	9.10419E-14	5.21367E-13	ENSGALG00000004412	A0A1L1RZ66	Diablo IAP-binding mitochondrial protein
ATF4	1.819309537	7.3788E-23	2.013E-21	ENSGALG00000012135	Q9W610	ATF4
CTSC	1.787015342	2.1643E-19	2.95219E-18	ENSGALG00000017239	F1NWG2	Cathepsin C
BIRC8	1.780424882	2.7752E-13	1.47834E-12	ENSGALG00000008491	Q8UVF8	Inhibitor of apoptosis protein 3
MAPK9	1.766861738	1.36925E-10	5.08567E-10	-	R0JIP5	Mitogen-activated protein kinase
JUN	1.731001898	1.21913E-18	1.43938E-17	-	A0A226PR05	Jun proto-oncogene, AP-1 transcription factor subunit
MCL1	1.645503256	1.0607E-19	1.54199E-18	ENSGALG00000044383	A0A1L1RUS7	BCL2 family apoptosis regulator

CTSB	1.627188736	4.1044E-20	6.40227E-19	ENSGALG00000030016	A0A1D5PUR5	Cathepsin B
KRAS	1.62426655	9.26685E-12	3.98322E-11	ENSMGAG00000013673	G1NLP3	GTPase KRas
H-RAS	1.619695863	1.08948E-15	8.14535E-15	-	A0A226MNN0	HRas proto-oncogene, GTPase
CFLAR	1.614494494	1.28028E-10	4.78313E-10	ENSGALG00000008240	E1C4U6	CASP8 and FADD like apoptosis regulator
PIK3CB	1.601271712	8.09993E-16	6.15998E-15	ENSGALG00000005505	Q5F4A2	Phosphatidylinositol-4,5-bisphosphate 3-kinase catalytic subunit beta
EIF2S1	1.559144402	4.23954E-19	5.50727E-18	-	A0A226NH66	Eukaryotic translation initiation factor 2 subunit alpha
FADD	1.531228568	1.18889E-17	1.2108E-16	ENSGALG00000007625	F1NKQ7	Fas associated via death domain
CYCS	1.503944506	1.67843E-15	1.22208E-14	-	A0A226P4Y8	Cytochrome c, somatic
CASP9	-1.499263422	1.49796E-05	3.09895E-05	ENSGALG00000001366	F1NL59	Caspase 9
MAP3K14	-1.549344095	7.19936E-14	4.1864E-13	ENSMGAG00000010881	G1NEI6	Mitogen-activated protein kinase kinase kinase 14
ENSGALG00000054561	-1.579335583	1.54903E-06	3.604E-06	ENSGALG00000040729	A0A1D5NZA7	p53-induced death domain protein 1
CTSH	-1.593932084	2.1589E-18	2.46495E-17	ENSGALG00000033557	A0A1D5PG47	Cathepsin H
GADD45G	-1.609998614	2.72886E-10	9.78177E-10	ENSGALG00000028005	Q2HZD5	Growth arrest and DNA damage inducible gamma
PIK3CD	-1.633225512	1.31586E-15	9.70209E-15	ENSGALG00000002583	F1NHX1	Phosphatidylinositol-4,5-bisphosphate 3-kinase catalytic subunit delta
DFFA	-1.635622439	5.71564E-15	3.84501E-14	ENSGALG00000002820	A0A1L1RU42	DNA fragmentation factor subunit alpha
gga-mir-3533	-1.654219577	2.16191E-15	1.54947E-14	-	G8DV45	Beta-actin
SPTAN1	-1.673168254	2.30422E-21	4.66006E-20	ENSGALG00000004719	A0A1D5PVG1	Spectrin alpha chain, non-erythrocytic 1
ACTB	-1.718350098	7.49688E-23	2.03545E-21	-	A0A218UWT5	Actin, cytoplasmic 1
TNFRSF1A	-1.904569379	9.75777E-18	1.00574E-16	ENSGALG00000039461	Q5ZJG1	TNF receptor superfamily member 1A
BID	-1.948847228	5.27662E-20	8.09293E-19	ENSGALG00000013039	A0A140T8G9	BH3-interacting domain death agonist
CAPN2	-1.958345731	6.2821E-08	1.70474E-07	ENSGALG00000009360	A0A1D5P8A6	Calpain-2 catalytic subunit
BCL2A1	-1.975594581	1.60529E-15	1.17148E-14	ENSGALG00000006511	Q9W6F2	BCL2 related protein A1

CTSO	-2.025606803	2.72825E-06	6.14467E-06	ENSGALG00000009373	Q5ZMK0	Cathepsin O
FOS	-2.060379269	2.98122E-20	4.77219E-19	ENSGALG00000028037	R4GH11	Proto-oncogene c-Fos
PIK3R5	-2.477521357	1.87004E-24	7.2902E-23	ENSGALG00000026167	F1NUD8	Phosphoinositide 3-kinase regulatory subunit 5
ERN2	-2.512369494	2.25685E-20	3.69569E-19	ENSGALG00000027592	R4GFM3	Endoplasmic reticulum to nucleus signaling 2
ENSGALG00000050441	-4.108902994	3.02721E-21	5.98072E-20	ENSGALG00000025881	R4GJW7	Cytokine receptor common subunit beta-like
