# Supplementary material for: Gene expression profiling of RIP2-knockdown in HD11 macrophages — elucidation of potential pathways (gene network) when challenged with avian pathogenic E.coli (APEC)
Source: BMC Genomics. 2022 May 2;23:341. doi: 10.1186/s12864-022-08595-5 (PMC9063279; doi:10.1186/s12864-022-08595-5)
Supplement: Supplementary file 2 — Additional file 2: Figure S1. Veen diagram for the MAPK signaling pathway in the contrast of knockdown of RIP2 HD11 cells (shRIP2) vs. wild type HD11 cells (WT) and knockdown of RIP2 combined with avian pathogenic E. coli infection HD11 cells (shRIP2 + APEC) vs. avian pathogenic E. coli infection HD11 cells (APEC), as well as the apoptosis pathway in the contrast of shRIP2 vs. WT and shRIP2 + APEC vs. APEC. Figure S2. Full-length blot of GAPDH protein expression level for Fig. 1B. Figure S3. Full-length blot of RIP2 protein expression level for Fig. 1B. Figure S4. Full-length blot of BID protein expression level for Fig. 10D. Figure S5. Full-length blot of CASP9 protein expression level for Fig. 10D. Figure S6. Full-length blot of HSP90AB1 protein expression level for Fig. 10D. Figure S7. Full-length blot of GAPDH protein expression level for Fig. 10D. [file 12864_2022_8595_MOESM2_ESM.pdf]

Legends for the supplementary figures

Figure S1 Veen diagram for the MAPK signaling pathway in the contrast of knockdown of *RIP2* HD11 cells (shRIP2) vs. wild type HD11 cells (WT) and knockdown of *RIP2* combined with avian pathogenic *E. coli* infection HD11 cells (shRIP2+APEC) vs. avian pathogenic *E. coli* infection HD11 cells (APEC), as well as the apoptosis pathway in the contrast of shRIP2 vs. WT and shRIP2+APEC vs. APEC.

Figure S2 Full-length blot of GAPDH protein expression level for figure 1B.

Figure S3 Full-length blot of RIP2 protein expression level for figure 1B.

Figure S4 Full-length blot of BID protein expression level for figure 10D.

Figure S5 Full-length blot of CASP9 protein expression level for figure 10D.

Figure S6 Full-length blot of HSP90AB1 protein expression level for figure 10D.

Figure S7 Full-length blot of GAPDH protein expression level for figure 10D.

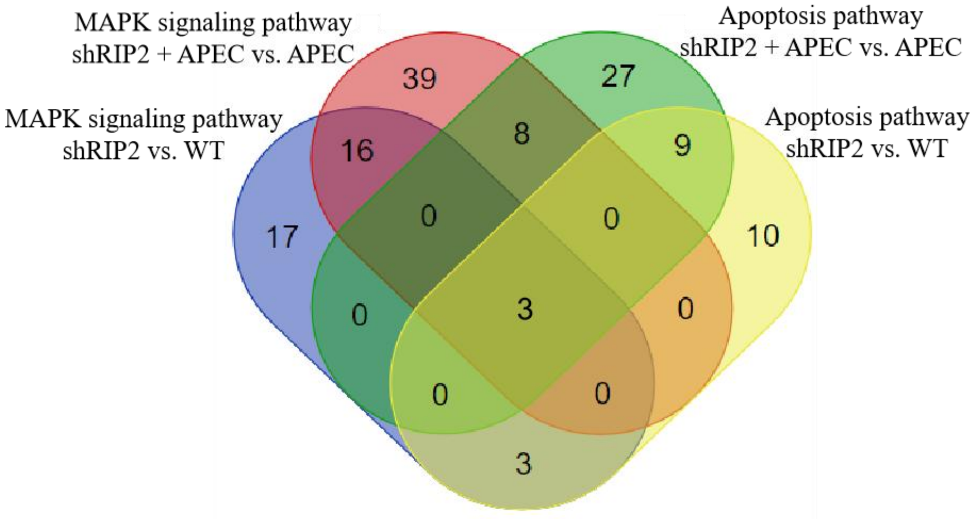

Figure S1 Veen diagram for the MAPK signaling pathway in the contrast of knockdown of *RIP2* HD11 cells (shRIP2) vs. wild type HD11 cells (WT) and knockdown of *RIP2* combined with avian pathogenic *E. coli* infection HD11 cells (shRIP2+APEC) vs. avian pathogenic *E. coli* infection HD11 cells (APEC), as well as the apoptosis pathway in the contrast of shRIP2 vs. WT and shRIP2+APEC vs. APEC

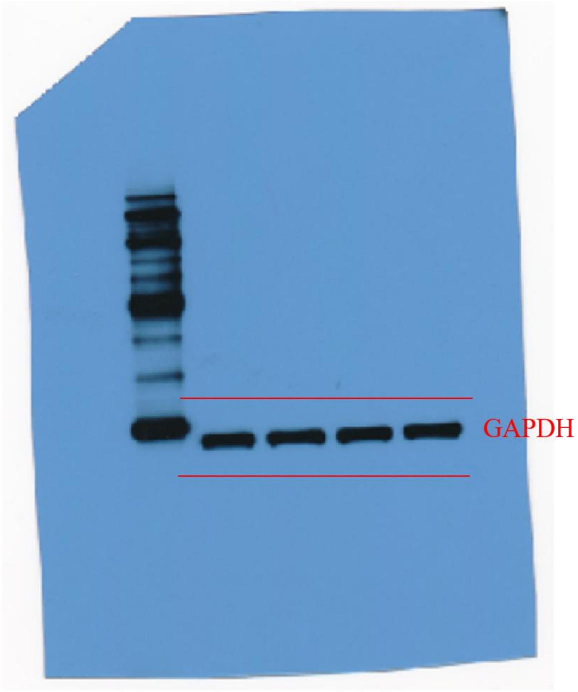

Figure S2 Full-length blot of GAPDH protein expression level for figure 1B.

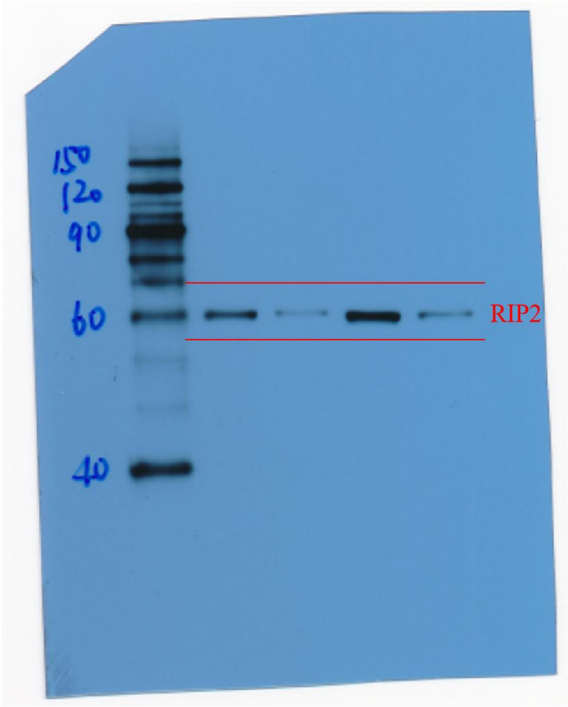

Figure S3 Full-length blot of RIP2 protein expression level for figure 1B.

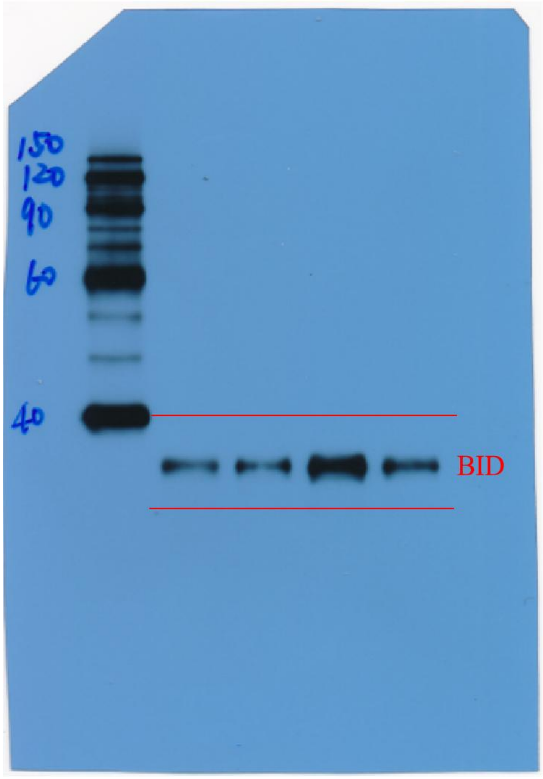

Figure S4 Full-length blot of BID protein expression level for figure 10D.

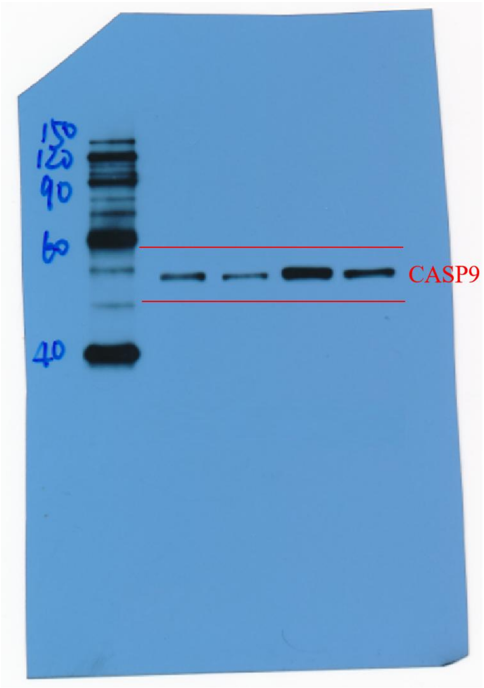

Figure S5 Full-length blot of CASP9 protein expression level for figure 10D.

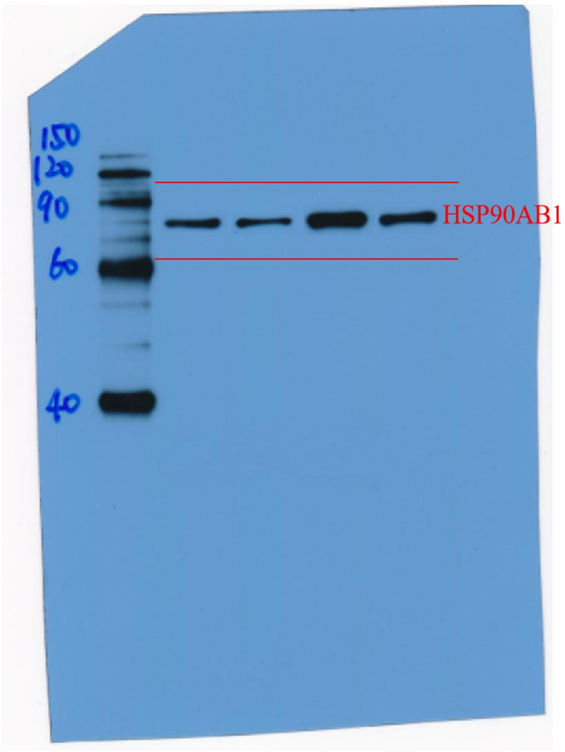

Figure S6 Full-length blot of HSP90AB1 protein expression level for figure 10D.

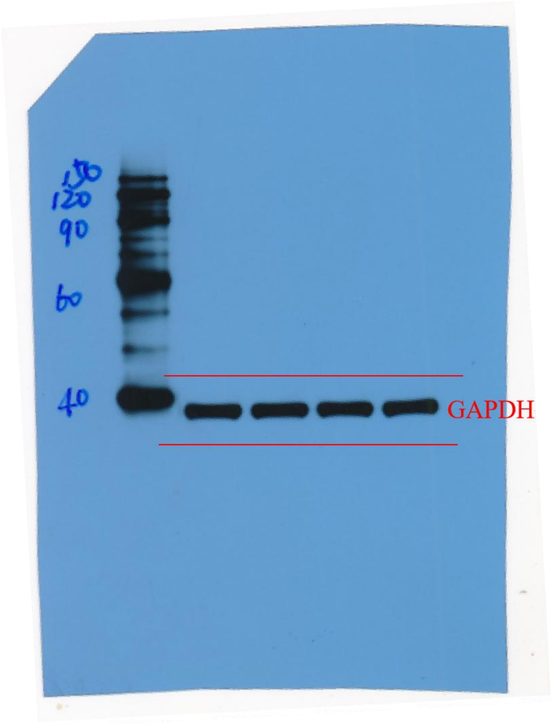

Figure S7 Full-length blot of GAPDH protein expression level for figure 10D.
